# An Efficient Peptidomics Screening for Exogenous Substrates and Inhibitory Peptides of the Dipeptidase ACE from Milk Hydrolysate

**DOI:** 10.3390/pharmaceutics15020425

**Published:** 2023-01-27

**Authors:** Ju-Hsuan Huang, Nhung Thi Phuong Nong, Jue-Liang Hsu

**Affiliations:** 1 Department of Biological Science and Technology, National Pingtung University of Science and Technology, 1 Shuefu Road, Neipu, Pingtung 91201, Taiwan; 2 Department of Basic Science, Thainguyen University of Agriculture and Forestry, Quyetthang Ward, Thai Nguyen 250000, Vietnam; 3 Department of Tropical Agriculture and International Cooperation, National Pingtung University of Science and Technology, Pingtung 91201, Taiwan; 4 Institute of Food Safety Management, National Pingtung University of Science and Technology, Pingtung 91201, Taiwan; 5 Research Center for Animal Biologics, National Pingtung University of Science and Technology, Pingtung 91201, Taiwan

**Keywords:** peptidomics, peptidase substrate, angiotensin-I-converting enzyme (ACE), stable-isotope labeling, LC-MS/MS

## Abstract

The dipeptidase angiotensin-I-converting enzyme (ACE) pre-incubation, liquid chromatography- mass spectrometry (LC-MS), and stable-isotope labeling were integrated for an efficient screening of ACE’s exogenous substrates from milk hydrolysate. Using this approach, 31 substrates were readily identified from 478 identified peptides and their activities were confirmed using synthetic peptides. Their reactivity is highly correlated with the decreased isotope ratio observed in LC-MS. Among these substrates, the most frequently observed residue at the P1′ position was Leu/Ser. It also revealed that ACE would not cleave the peptide when P1′ is Pro, P2′ is Asp/Glu, or P1 position is Ile. Interestingly, the sequential two-stage hydrolysis was also found. Moreover, their protective effects against ACE-mediated hydrolysis of angiotensin I (Ang-I) were also examined. The result indicated that AYFYPELFR and HLPLPLLQSW can significantly retard the hydrolysis of Ang-I and act as substrate-type inhibitors.

## 1. Introduction

More than 500 human genes (approximately 2%) are encoded as proteases or peptidases. However, the biological roles played by these enzymes are still largely unclear [1], since the physiological substrates are typically unknown and are difficult to identify on a large scale using traditional analytical methods [2]. Peptidases play an important role in regulating physiological mechanisms by cleaving endogenous peptide substrates. For example, dipeptidyl peptidase 4 (DPP4) is mainly responsible for degrading incretin hormones, glucagon-like peptide-1 (GLP-1) and glucose-dependent insulinotropic polypeptide (GIP). Both hormones are released after meal ingestion and promote insulin secretion from pancreatic β cells, thereby regulating postprandial blood glucose [3]; angiotensin-I-converting enzyme (ACE) plays dual roles in regulating blood pressure by catalyzing the formation of the strong vasoconstrictor angiotensin II from angiotensin I as well as triggering the inactivation of bradykinin, an important vasodilator. Angiotensin II can bind to the AT1R receptor, promote the secretion of aldosterone, stimulate the contraction of vascular smooth muscle, and increase blood pressure [4].

Peptidomics is the comprehensive study of all peptides in a biological system with plenty of qualitative and quantitative information [5]. It can be classified as a branch of proteomics that targets endogenous peptides within the body. Therefore, peptidomics is ideally suited to a large-scale screening of endogenous peptidase substrates. Typically, the stable-isotope labeling, or label-free approach coupled with liquid chromatography-tandem mass spectrometry (LC-MS/MS) were used for peptidomics analysis [6,7,8,9]. For example, Tinoco et al. utilized an LC-MS-based peptidomics platform to measure the changes in the peptidome as a function of peptidase activity, and by doing so, they identified over 70 renal DPP4 substrates from the tissues of DPP4-/- mice [10]. Yates et al. used a label-free approach coupled with FTMS differential mass spectrometry to successfully uncover 70 DPP4 substrate candidates from the peptide library derived from human plasma [2].

**Figure 1:**
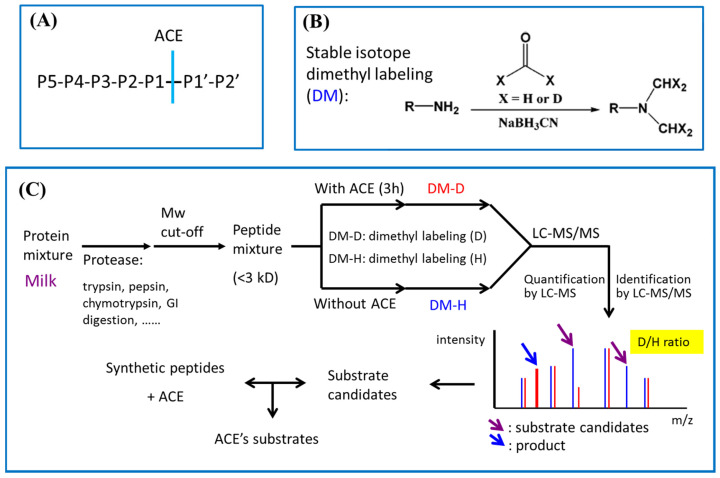
The concept of LC-MS/MS based exogenous substrate screening. (**A**) The representation of peptide’s typical cleavage site towards the dipeptidase ACE. (**B**) The reaction scheme of stable isotope dimethyl labeling (DM). (**C**) The workflow for the screening of ACE’s substrate using stable-isotope labeling and LC-MS/MS analysis.

ACE has been described as a peptidyldipeptidase or dipeptidyl carboxypeptidase. It is a well-known pharmaceutical target for treatment of cardiovascular diseases, in particular hypertension [11]. ACE plays a critical role in the renin–angiotensin system (RAS), which controls blood pressure by regulating the conversion of the hormone Ang-I to the active vasoconstrictor angiotensin II (Ang-II). In addition to Ang-I, ACE can also degrade bradykinin [12], substance P (Sub P) [13], and amyloid beta-protein [14]. ACE cleaves substrates other than Ang-I or bradykinin by peptidyl dipeptidase action, which cuts P1-P1′ amide bond and releases a C-terminal dipeptide, as shown in Figure 1A. Due to its broad activity on a variety of endogenous peptides, the substrate specificity of ACE has aroused the interest of physiologists. In addition to substrates in humans, exogenous ACE substrates derived from food protein hydrolysates have been attractive targets for the pharmaceutical and nutraceutical industries, because these substrates can also exert ACE inhibitory (ACEI) activity, and act as substrate-type or prodrug-type inhibitors [15]. To the best of our knowledge, the studies of ACE’s substrate specificity are primarily based on a limited number of synthetic peptides rather than a peptidomics approach [16,17].

In this study, we developed a novel and efficient approach to screen for exogenous substrates of ACE. The substrate specificity of ACE was also explored and confirmed using synthetic peptides. We believe this approach is also promising in the discovery of substrates for other peptidases, and may even benefit the development of related drugs. This peptidomics approach includes ACE pre-incubation, stable-isotope dimethyl labeling (Figure 1B), and LC-MS/MS analysis (The workflow was shown in Figure 1C). Four peptide libraries were prepared from milk proteins hydrolysates generated by pepsin, trypsin, α-chymotrypsin, and combinations of these three proteases, respectively. The small peptides of each hydrolysate, obtained using a molecular weight cut-off (3 kDa) membrane, were divided into two aliquots. One aliquot was directly modified using stable-isotope dimethyl labeling [18] with a hydrogen atom (DM-H); while, the other aliquot was incubated with ACE for 3h followed by dimethyl labeling with a deuteron atom (DM-D) (Figure 1B). The samples with and without ACE treatment were combined and analyzed using LC-MS/MS to give identification and quantification information from MS/MS and MS spectra, respectively. The decreased D/H ratio (<1) was due to the cleavage of ACE, suggesting that the peptides with decreased or disappeared DM-D labeled form can be regarded as ACE’s substrate candidates. Meanwhile the substrate candidates were accompanied by their corresponding products with DM-D labeling, as shown in Figure 1C. Notably, the products, which were labeled with DM-D form, only existed in the ACE-treated group. The reactivity of identified substrate candidates against ACE were further validated using the synthetic peptides. The sequence conservation of identified substrates was analyzed using WebLogo (https://weblogo.berkeley.edu, accessed on 9 November 2020) [19] to conclude the specificity of exogenous substrates towards ACE. In addition, these substrates’ inhibitory activities against ACE were also examined using ACEI assay and a pre-incubation experiment to characterize these substrates as real substrates or pro-drug type inhibitors. Moreover, these ACEI peptides were co-incubated with Ang-I to evaluate their protection effects on ACE-mediated hydrolysis of Ang-I.

## 2. Materials and Methods

### 2.1. Materials

Milk powder was purchased from Fonterra Co-operative Group, Taiwan. Pepsin (from porcine gastric mucosa, ≥250 units/mg), trypsin (from bovine pancreas, ≥10,000 BAEE units/mg), α-chymotrypsin (from bovine pancreas, ≥40 units/mg), ACE (EC 3.4.15.1, 1 unit) from rabbit lungs, ammonium bicarbonate (ABC), Captopril, hipppuryl-L-histidyl-L-Leucine (HHL), ferulic acid, formaldehyde-D2 (20% solution in D2O), hydrogen chloride (HCl), sodium chloride (NaCl), sodium hydroxide (NaOH), ethyl ether, triisopropylsilane (TIS) and N,N′-diisopropylacarbodiimide (DIC) were purchased from Sigma Chemical Co. (St. Louis, MO, USA). Trifluoroacetic acid (TFA), N,N-Diisopropylethylamine (DIPEA), 4-Methylmorpholine (NMM), and O-(1H-benzotriazol-1-yl)-N,N,N′,N′-tetramethyl-hexafluorophosphate (HOBt) were obtained from Alfa Aesar (Royston, Hertfordshire, UK). Acetic acid, acetonitrile (ACN), formic acid (FA), boric acid, potassium phosphate, piperidine, tris hydrochloride (Tris-HCl), formaldehyde (37% solution in H_2_O), and HPLC-grade methanol (MeOH) were purchased from J.T.Baker (Phillipsburg, NJ, USA). Oxyma pure and Fmoc-protected amino acids were obtained from Novabiochem^®^ (Billerica, MA, USA). Methylene chloride (DCM) and N,N-Dimethylformamide (DMF) were purchased from Duksan (Ansan, Republic of Korea). Wang resin was purchased from Cleo Salus (Louisville, KY, USA). The deionized water was generated using a PURELAB^®^ water purification system from ELGA LabWater (Lane End, High Wycombe, UK).

### 2.2. Peptide Libraries Generated by Enzymatic Hydrolysis of Skimmed Milk Powders

Individually, three kinds of proteases, trypsin, α-chymotrypsin, and pepsin, were added to 10 mg of skimmed milk powders in an enzyme-to-substrate ratio of 1:50 (*w*/*w*). The hydrolysis conditions for both trypsin (37 °C) and α-chymotrypsin (37 °C) were kept at pH 8.5 adjusted with 50 mM ABC, while the reaction of pepsin (37 °C), dissolved in 20 mM NaCl, was maintained at pH 1.3 adjusted using 4M HCl. After 16 h of incubation, all the hydrolysis reactions were quenched by heating at 95 °C for 10 min. The hydrolysates were filtered through the 3 kDa MWCO ultrafiltration membrane (Amicon^®^ Ultra-0.5) (Merck Millipore, Darmstadt, Germany) by centrifugation at 14,000 rpm/4 °C for 15 min (Hitachi Koko Co, Japan) to obtain short-chain peptides (<3 kDa) as the peptide libraries. For the peptide library generated by simulated gastrointestinal digestion, the skimmed milk powder (10 mg) was hydrolyzed using pepsin at 37 °C for 16 h with an enzyme to protein ratio of 1:100 (*w*/*w*), and dissolved in 35 mM NaCl adjusted pH using HCl to pH 1.9. In the second stage of hydrolysis, the hydrolysate mixture was adjusted until the pH was 8.3 using NaOH, then further hydrolyzed using trypsin and α-chymotrypsin with enzyme–substrate ratio (E/S = 1:100 *w*/*w*) for 16 h at 37 °C. Afterwards, the short-chain peptides were obtained by the filtration using 3 kDa MWCO ultrafiltration membrane. The resulting hydrolysates were desalted using the PepClean™C18 spin column (Thermo Scientific, Rockwood, TN, USA). The peptide libraries generated using trypsin, α-chymotrypsin, pepsin, and simulated gastrointestinal digestion were lyophilized and kept at −20 °C prior to further screening assays.

### 2.3. ACE Incubation and Stable-Isotope Dimethyl Labeling

The peptide libraries (200 μg) dissolved in borate buffer were equally divided into two parts, one was without any treatment (control group); the other part (experimental group) was incubated with 0.05 mU/μL ACE at 37℃ for 1.5 h statically, followed by gentle shaking at 200 rpm for another 1.5 h, and then the ACE reaction was quenched using 60 μL of 1N HCl. After lyophilization, both samples were redissolved in 100 μL of 0.1M sodium acetate and modified using stable-isotope dimethyl labeling according to the previous study [18] with slight modifications. Briefly, the control group (without ACE incubation) had 4 μL of formaldehyde (37% solution in H_2_O) and 1 M NaBH_3_CN (10 μL) added to it; the experimental group had 20% formaldehyde-d2 (8 μL) and 1M NaBH_3_CN (10 μL) added to it. After 1h, 5 μL of ammonia (28% solution in H_2_O) were added individually to both groups to stop the reaction. The experimental group and the control group were combined and desalted using the PepClean™C18 spin column. The resulting peptide mixture was lyophilized and kept at −20 °C prior to LC-MS/MS analysis. Formaldehyde is known to the state of California to cause cancer; special caution was taken including the use of surgical gloves and a fume hood when formaldehyde was handled.

### 2.4. Identification of Substrate Candidates Using LC-MS/MS

The combined mixture re-dissolved in 5% ACN containing 0.1% formic acid was separated using an Ultimate 3000 RSLC system (Dionex, Sunnyvale, CA, USA) coupled with a C18 column (Acclaim PepMap RSLC, 75 μm × 150 mm, Thermo Scientific, Waltham, Middlesex, MA, USA) and analyzed with a Thermo Q-ExactiveTM mass spectrometer (Thermo Scientific Inc., Waltham, Middlesex, MA, USA). The elution solution was composed of mobile phase A (0.1% formic acid in water) and B (0.1% formic acid in 95% acetonitrile). The elution gradient was arranged as follows: (i) isocratic elution using 1% B in the first 5.5 min for sample loading, (ii) linear gradient from 1% to 60% B in the next 39.5 min, (iii) linear gradient from 60% to 80% B in the following 10 min, and finally (iv) isocratic elution using 1% solution B for 10 min. The flow rate applied in the LC separation was maintained at 250 µL/min. The separated peptides were analyzed and quantified using Q-ExactiveTM mass spectrometry which was operated in the data-dependent mode and switched automatically between MS and HCD-MS2. A full MS scan was carried out in a 100 and 1500 *m*/*z* range, and the 14 ions of the highest intensities were selected for MS/MS scans. The RAW data were converted to the MGF file format followed by database-assisted sequencing with PEAKS DB using PEAKS Studio X (Bioinformatics Solutions Inc., Waterloo, ON, Canada). The parameters used for peptide identification were listed as follows: (1) Database: UniProt_SwissProt (Bos taurus); (2) Enzyme: pepsin, trypsin, α-chymotrypsin, or GI (the combination of these three proteases); (3) Type of enzyme: Unspecific; (4) Instrument: Orbitrap; (5) Parent mass error tolerance: 20 ppm; (6) Fragment mass error tolerance: 0.6 Da; (7) Variable modifications: Dimethyl (K), Dimethyl (N-term), Dimethyl:2H(4) (K), and Dimethyl:2H(4) (N-term) were used for identification and quantitative analysis. The sequence identified through the database was validated by manually matching with the MS/MS spectrum in the raw data. In addition to MS/MS analysis coupled with PEAKS studio, the ACE’s substrate candidates were further confirmed using the synthetic peptides with corresponding sequences by comparison with their LC retention times, *m*/*z* values, and MS/MS spectra. The relative abundances of hydrogen (H) and deuterium (D) labeled peptides were quantified based on the peak areas in their corresponding selective ion chromatograms (SIC). The D/H ratio of identified peptide with the same sequence was further examined by their relative signal intensities in MS spectrum. Theoretically, the D/H ratio of the isobaric pair should stay at 1 if the peptide will not react with ACE. The decreased D/H ratio (<1) was due to the cleavage by ACE, which suggested that the peptides with decreased or disappeared DM-D labeled form can be regarded as ACE’s substrate candidates. Once the substrate candidates were identified, their corresponding products with DM-D labeling should also be characterized.

### 2.5. Synthesis of Substrate Candidates and Confirmation of Their Reactivities towards ACE

To confirm the reactivities of the identified substrate candidates, peptide synthesis was performed according to their sequences identified by LC-MS/MS. The peptides were synthesized starting from C-terminal to the N-terminal on the solid support of Wang resins in the CEM Discover reactor (CEM Microwave Technology Ltd., Buckingham, UK), according to our previous report [20]. The synthetic peptides were purified using RP-HPLC and their identities were validated by LC-MS/MS.

### 2.6. Monitoring the ACE Hydrolysis of Substrate Candidates at Different Incubation Times

To confirm the reactivity of the identified peptide candidates towards ACE, the synthetic peptides were individually mixed with ACE and incubated at 37 °C for 3 h. The remaining peptide substrate and the resulting product were analyzed using LC-MS/MS. Meanwhile, the degradation rate of each peptide was monitored every 30 min using LC-MS. The percentage of the remaining substrate (RS%) at each time point was calculated as *A_s_*/(*A_s_* + *A_p_*) 100%. In which, *A_s_* is the selective ion chromatogram (SIC) peak area of remaining substrate and *A_p_* is the peak area of the resulting product. The RS% was further compared with the D/H ratio obtained in Section 2.4.

### 2.7. ACE Inhibitory Assay of Identified Peptide Substrates

The ACE inhibitory assay was performed based on the previous report [20] with slight modification. Briefly, 30 µL of 2.5 mM hipppuryl-L-histidyl-L-Leucine (HHL) was preincubated with peptide substrate at 37 °C for 5 min. Afterwards, 20 µL of ACE (0.05 mU/µL) in borate buffer was added. The mixture was first incubated at 37 °C statically for 30 min, and then shaken at 200 rpm for another 30 min. The reaction was terminated using 60 µL of HCl (1 M). The hydrolyzed product of HHL, HA, was analyzed by HPLC (Hitachi Chromaster, Tokyo, Japan) equipped with a NUCLEODUR^®^ C_18_ HTec column (4.6 mm × 250 mm; particle size 5 µm, Macherey-Nagel, Düren, Germany) and quantified by its chromatographic peak area. The mixture was separated using an isocratic elution with the solution containing 24% ACN and 0.1% TFA at a constant flow rate of 1 mL/min. The substrate and product were monitored using a UV-Vis detector at a wavelength of 228 nm. The ACEI activity (%) was calculated as shown below:

ACEI activity (%) = [1 – (*A_inhibitor_*/*A_blank_*)] × 100%

where *A_inhibitor_* and *A_blank_* were the resulting HA peak areas in the mixtures with and without inhibitors, respectively. Along with the peptide substrate’s ACEI activity, its IC_50_ value was also determined using a nonlinear regression between ACE inhibition (%), and the logarithm of inhibitor concentrations could be carried out with GraphPad Prism 5.0 (GraphPad Software, Inc., La Jolla, CA, USA).

### 2.8. ACE Hydrolysis towards the Angiotensin I Co-Incubated with Peptide Substrates

The Ang-I and Ang-II were synthesized, purified, and identified according to the method mentioned in Section 2.5. To a mixture of Ang-I (1 μg) and peptide substrate (1 μg) in 50 µL of borate buffe (200 mM borate buffer, 300 mM NaCl, pH 8.3), 20 µL of ACE (0.05 mU/µL) was added. The mixture was incubated at 37 °C and monitored using LC-MS/MS at 1, 2, 3, 4, 6, and 10 h. The LC separation was performed on Syncronis C18 column (150 mm × 2.1 mm, 5 µm, Thermo Scientific) and eluted with the mobile phase A (5% ACN and 0.1% FA) and B (95% ACN and 0.1% FA) at a flow rate of 350 µL/min. The elution gradient was programmed as (i) 0–5 min, isocratic elution with 5% B; (ii) 5–20 min, linear gradient from 5% to 55% B; (iii) 20–22 min, linear gradient from 55% to 80% solution B; and finally (iv) 22–30 min, isocratic elution maintained at 80% solution B. The degradation of Ang-I and the formation of Ang-II were analyzed using selective ion chromatogram (SIC) on a triple quadrupole mass analyzer (TSQ, Thermo Scientific). The percentage of the remaining angiotensin I (RA%) at each time point was calculated as *A_I_*/(*A_I_* + *A_II_*) × 100%, where *A_I_* is the SIC peak area of remaining Ang-I and *A_II_* is the peak area of the resulting Ang-II.

### 2.9. Statistical Analysis

The inhibitory assays of peptide substrates were performed in triplicate and the data were expressed as the mean ± standard deviation. Significant differences between means were determined by analysis of variance (ANOVA) and Dunnett’s multiple comparison at the resulting p-value lower than 0.05 (*p* < 0.05).

## 3. Results and Discussion

### 3.1. Identification of ACE Substrate Candidates from Milk Hydrolysates

**Figure 2:**
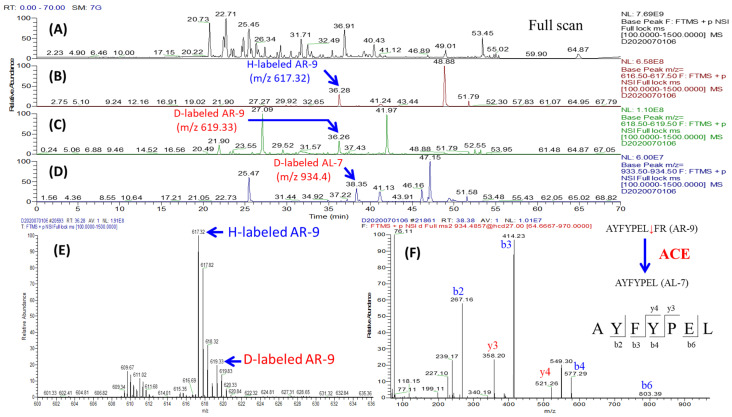
The identification of the substrate candidate AYFYPELFR (AR-9). (**A**) The full LC-MS chromatogram of the combined sample in Figure 1. (**B**) The selective ion chromatogram (SIC) of H-labeled AR-9 (without ACE treatment). (**C**) The SIC of D-labeled AR-9 (after ACE treatment). (**D**) The SIC of AYFYPEL (AL-7), the hydrolysis product of AR-9 after ACE treatment. (**E**) The MS spectrum of isobaric pair of AR-9 at retention time (tR) of 36.2 min. (**F**) The MS/MS spectrum of D-labeled AL-7.

**Figure 3:**
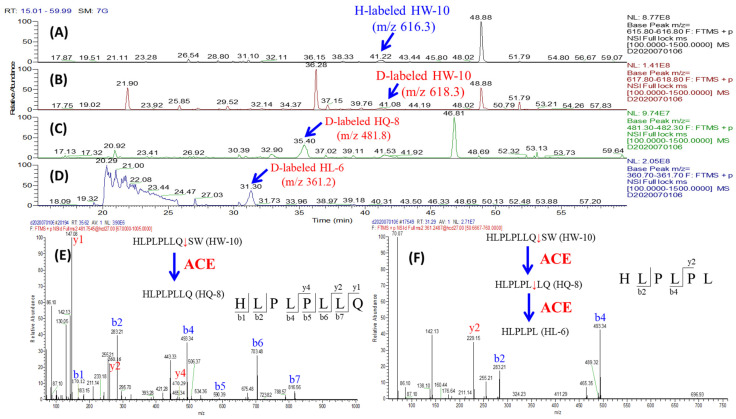
The identification of sequential hydrolysis of HLPLPLLQSW (HW-10). (**A**) The SIC of H-labeled HW-10. (**B**) The SIC of D-labeled HW-10. (**C**) The SIC of D-labeled HLPLPLLQ (HQ-8, the product of the first hydrolysis by ACE). (**D**) The SIC of HLPLPL (HL-6, the product of the second hydrolysis). (**E**) The MS/MS spectrum of D-labeled HQ-8. (**F**) The MS/MS spectrum of D-labeled HL-6.

To screen ACE’s exogenous substrates from milk hydrolysates, the milk proteins were digested using trypsin, α-chymotrypsin, pepsin, and a combination of these three gastrointestinal enzymes. The resulting hydrolysates were pooled and filtered using a 3 kDa MWCO ultrafiltration membrane to obtain a small peptide library. The peptide pool was equally divided into two groups. One group was directly labeled with hydrogen (H) atoms, and the other group was pre-incubated with ACE and then labeled with deuterium (D) atoms. Afterwards, the two groups were combined for LC-MS/MS analysis. Theoretically, if the peptide cannot be hydrolyzed by ACE, the D/H ratio of the isotope pair should be close to 1, which means that the peptide is not a substrate of ACE. The decreased D/H ratio (<1) of the peptides was due to their hydrolysis by ACE, suggesting that peptides with decreased or disappeared deuterium (D) labeled form may be substrates of ACE. Once a substrate candidate (D/H ratio < 1) has been identified, its corresponding product with a DM-D labeled form should also be observed, as shown in Figure 1C. The identified peptides that could meet the above two requirements were considered as substrate candidates for ACE. Using this approach, 31 substrate candidates were readily identified from 478 identified peptides. All of the identified substrate candidates are summarized in Table 1. Identification of substrate candidates is demonstrated by the following examples. The full LC-MS chromatogram of combined samples, H-labeled (control group) and D-labeled (experimental group), is shown in Figure 2A. The selective ion chromatograms (SIC) of H- and D-labeled AYFYPELFR (AR-9) can be extracted from Figure 2A, as Figure 2B,C, respectively. The abundance of D-labeled AYFYPELFR (AR-9) was much less than that of H-labeled AR-9 because most AR-9 was hydrolyzed by ACE in the experimental group. The relative abundance of AR-9 in the experimental and control groups was determined using the intensity ratio of D-labeled and H-labeled AR-9 to give a D/H ratio of 0.19 in the MS spectrum (Figure 2E). Meanwhile, AYFYPEL (AL-7), the hydrolysis product of AR-9, was simultaneously observed in the combined sample. The SIC and MS/MS spectrum of D-labeled AL-7 are shown in Figure 2D,F, respectively. Similarly, the identification of other substrate candidates is summarized in Figures S1–S30 in Supporting Information. Interestingly, some peptides can undergo sequential two-stage hydrolysis by ACE. For instance, the amount of HLPLPLLQSW (HW-10) in the ACE-treated group (Figure 3A) was dramatically reduced when compared with the control group (Figure 3B). Meanwhile, two hydrolysis products, HLPLPLLQ (HQ-8) (Figure 3C) and HLPLPL (HL-6) (Figure 3D), were also observed with only the D-labeled form instead of the H-labeled form, which indicated that HQ-8 was derived from HW-10, and HL-6 was generated from HQ-8. The formation and MS/MS spectra of HQ-8 and HL-6 were shown in Figure 3E and Figure 3F, respectively. The ACE’s stepwise hydrolysis towards its substrates is common in physiological conditions. For instance, ACE is able to hydrolyze bradykinin (RPPGFSPFR) to bradykinin (1–7) (RPPGFSP), and then the resulting bradykinin (1–7) is further hydrolyzed by ACE to form bradykinin (1–5) (RPPGF) in vivo; similarly, Sub P (RPKPQQFFGLM) will be cut to Sub P (1–9) (RPKPQQFFG) and Sub P (1–9) will be further converted to Sub P (1–7) (RPKPQQF) in the presence of ACE [21]. However, to the best of our knowledge, this is the first study reporting on ACE’s stepwise hydrolysis of exogenous substrates.

### 3.2. In Vitro Reactivity Confirmation of the ACE’s Substrate Candidates Using Synthetic Peptides

**Figure 4:**
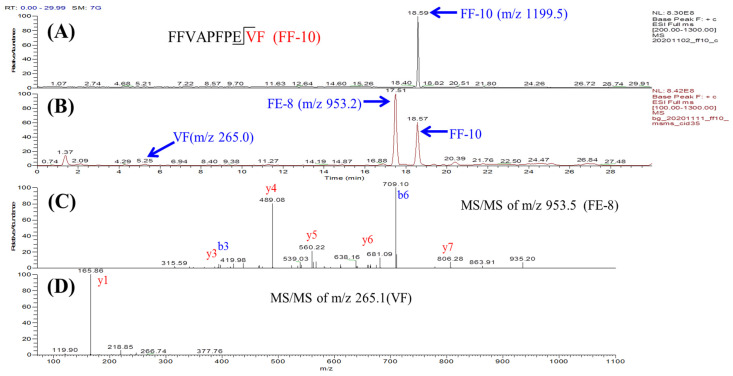
The reactivity confirmation of FFVAPFPEVF (FF-10). (**A**) The full chromatogram of FF-10. (**B**) The full chromatogram of FF-10 after ACE incubation for 3h. (**C**) The MS/MS spectrum of FE-8. (**D**) The MS/MS spectrum of VF.

**Figure 5:**
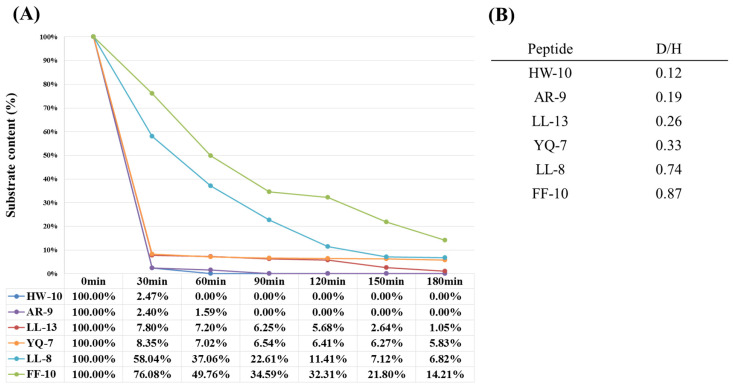
The reaction rate correlation of six peptides in hydrolysate and synthetic peptides. (**A**) The relative contents (%) of six synthetic peptides incubated with ACE at different times. (**B**) The relative contents (D/H ratios) of six peptides observed in the combination of ACE-treated and untreated samples (milk hydrolysates).

To verify the reactivities of ACE’s substrate candidates, synthetic peptides were incubated with ACE and the remaining peptides were monitored using LC-MS every 30 min. Six peptides with D/H ratios ranging from high to low D/H ratio were selected for in vitro reactivity confirmation. The selected peptides were FFVAPFPEVF (FF-10, D/H = 0.87), LHLPLPLL (LL-8, D/H = 0.74), YVPLGTQ (YQ-7, D/H = 0.33), LVYPFPGPIHNSL (LL-13, D/H = 0.26), AYFYPELFR (AR-9, D/H = 0.19), and HLPLPLLQSW (HW-10, D/H ratio = 0.12). After incubation with ACE at 37 °C for 3 h, these six synthetic peptides were hydrolyzed by ACE at different reaction rates. For example, FF-10 was partially hydrolyzed into its product FFVAPFPE (FE-8) and VF, as shown in Figure 4. After incubation with ACE, the abundance of FF-10 (Figure 4A) was decreased and accompanied with the formations of FE-8 and VF (Figure 4B) which were verified using their MS/MS spectra, as shown in Figure 4C and Figure 4D, respectively. The confirmation of the other five substrates was summarized in Figures S31–S35 in Supporting Information. The conversion yields of the six synthetic substrates hydrolyzed by ACE were further correlated with those quantified as D/H ratios of the corresponding peptides in hydrolysate, as shown in Figure 5. Figure 5A shows the relative remaining content of six substrates monitored by LC-MS every 30 min in the presence of ACE. The conversion yield is determined as the following order: HW-10 > AR-9 > LL-13 > YQ-7 > LL-8 > FF-10, of which the trend is perfectly consistent with the D/H ratios obtained in the combined sample of ACE-treated (D-labeled) and untreated (H-labeled) hydrolysates (Figure 5B). The result revealed that this peptidomics approach can efficiently screen ACE’s substrates in large-scale, and the resulting D/H ratios of identified substrates can be used as the evaluation metrics for their reactivities towards ACE.

### 3.3. The Sequence Conservation of ACE’s Exogenous Substrates

**Figure 6:**
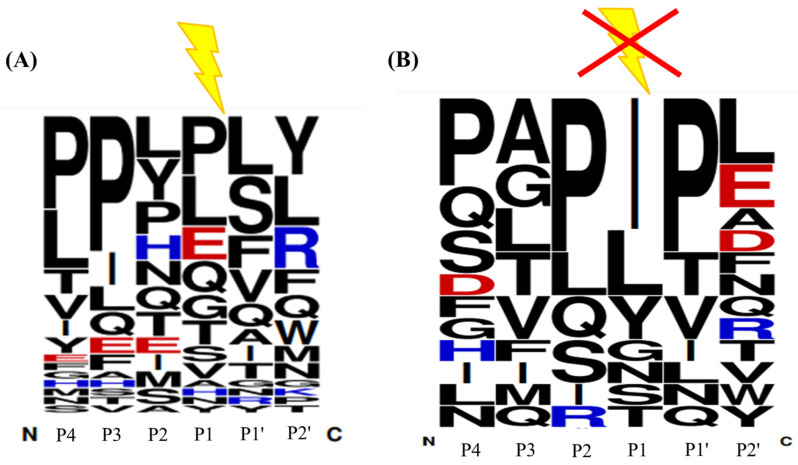
The sequence conservation of ACE’s exogenous substrates (**A**) and non-substrates (**B**) (analyzed using WebLogo). The substrates’ amide bonds between P1 and P1′ residues will be cleaved by ACE. The peptides (with D/H ratio ~ 1) are regarded as non-substrates and their amide bond between P1 and P1′ residues will not be cleaved by ACE. The font size of each letter (residue) represents the frequency appearance of each residue at a certain position. The order from the most frequent to the least frequent is arranged from the top to the bottom for each residue.

**Figure 7:**
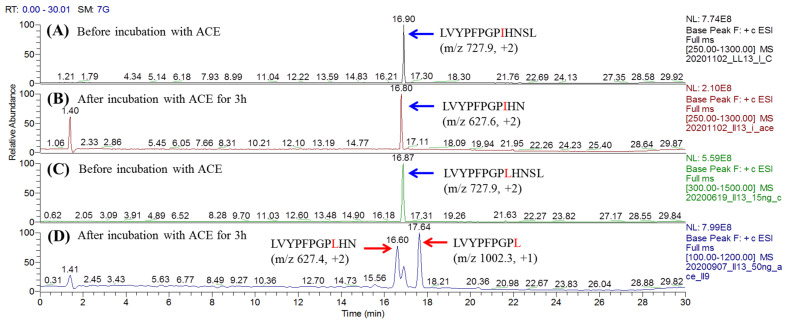
The effect of Ile (at P1 position) on ACE hydrolysis. LC-MS chromatograms of (**A**) LVYPFPGPIHNSL without ACE hydrolysis; (**B**) LVYPFPGPIHNSL after incubation with ACE for 3 h; (**C**) LVYPFPGPLHNSL without ACE hydrolysis; (**D**) LVYPFPGPLHNSL after incubation with ACE for 3 h.

To investigate the sequence conservation of the exogenous substrates, the identified substrates listed on Table 1 were analyzed using Weblogo (https://weblogo.berkeley.edu/) [19]. WebLogo is the software designed to analyze the frequency of residues that appeared at various positions in a sequence library. The font size of each letter (residue) represents the frequency appearance of each residue at a certain position. The order from the most frequent to the least frequent is arranged from the top to the bottom for each residue. The chemical properties of amino acid residues are classified by various colors, such as black presented for hydrophobic residues (A, V, L, I, P, W, F, and M), green for polar (G, S, T, Y, and C), red for acidic (D and E), pink for neutral (Q and N), and basic (K, R, and H) amino acids being blue. The Weblogo was performed on the C-terminal of six residues of thirty-one substrates and four first-stage products during the ACE sequential hydrolysis. The result indicated that Leu, Ser, and Phe are the top three residues most observed at the P1′ position; meanwhile, Pro and Leu are the most frequently observed residues at the P1 position, as shown in Figure 6A. This conclusion is similar to those observations reported previously. For example, Skidgel et al. found that the primary cleavage site (P1′ position) of Sub P (RPKPQQFFGLM) by ACE was Leu [12]. The P1′ position of ACE’s endogenous substrate bradykinin (1–7) (RPPGFSP) is Ser, while the P1′ of Ang (1–9) (DRVYIHPFH), bradykinin (RPPGFSPFR), and Sub P (1–9) (RPKPQQFFG) are Phe [21]. Similarly, the P1 position of some ACE’s endogenous substrates, such as Ang (1–9) (DRVYIHPFH) and bradykinin (RPPGFSPFR), are Pro [21]. Bersanetti et al. designed a series of hexapeptide Abz-GXXZXK(Dnp)-OH [Abz = o-aminobenzoic acid, K(Dnp) = Nε-2,4-dinitrophenyllysine, X = a random residue, and Z = one of the 19 natural amino acids except for Cys] as a substrate to study the residue specificity at P1 and P1′ for ACE. Their result indicated that the peptides containing Leu in the P1 position had higher C-domain ACE selectivity [22], which is similar to our finding. In this study, the sequences of identified non-substrates (with D/H ratio close to 1) were also analyzed using WebLogo for the prediction of residue that hinders ACE’s hydrolysis. As shown in Figure 6B, the non-substrates commonly contain Pro at the P1′ position, or Glu, Ala, or Asp at P2′, which revealed that ACE cannot easily cleave the peptide whose P1′ position is Pro or P2′ is Glu, Ala, or Asp. This finding is consistent with the specificity reported previously [23,24]. Two peptides NIPPLTQTPV (NV-10) and IASGEPTSTPTTE (IE-13), identified as non-substrates in this study, were synthesized and used to confirm the prediction. The result indicated that Pro at P1′ position (peptide NV-10) or Glu at P2′ (peptide IE-13) did avoid ACE’s hydrolysis after 3h incubation, as shown in Figures S36 and S37 in Supporting Information. Prolines with secondary amines show higher steric hindrance than regular primary amines. Therefore, the Pro residue at the P1′ position may block ACE hydrolysis. Notably, we also found that Ile at the P1 position will also block the ACE’s catalytical cleavage. This may be due to the steric hindrance or hydrophobicity of the aliphatic branched side chain of Ile at the P1 position that impedes the access of ACE. To validate this conclusion, two synthetic peptides LYQGPIVL and VRSPAQILQ (Ile at P1) were individually incubated with ACE for 3 h and the reaction was monitored using LC-MS/MS. These data show that they can survive from ACE’s hydrolysis, as shown in Figures S38 and S39. This result is similar to that reported by Forghani et al. [21], which showed that CRQNTLGHNTQTSIAQ (derived from *Stichopus horrens*) had only 7% peptide loss at 3 h of incubation with ACE. Moreover, this finding can also explain why the substrate LVYPFPGPIHNSL (LL-13) identified in this study did not undergo the second stage hydrolysis by ACE. After incubation with ACE for 3h, the resulting product LVYPFPGPIHN, containing Ile at P1 position, will therefore retard the second cleavage (Figure 7B). When the Ile (I) at LVYPFPGPIHNSL was replaced with Leu (L), the synthetic peptide LVYPFPGPLHNSL was readily hydrolyzed by ACE to give the first hydrolysis product LVYPFPGPLHN, and as expected, it was subsequently converted to LVYPFPGPL due to the lack of Ile at the P1 position, as shown in Figure 7D. To the best of our knowledge, this is the first finding that Ile at the P1 position will retard the release of C-terminal dipeptide catalyzed by ACE.

### 3.4. Determination of The ACE Inhibitory (ACEI) Effect and Types for the Identified Substrates

Three inhibition types including true inhibitor, real substrate, and prodrug can be classified based on the IC_50_ value after the preincubation experiment. The IC_50_ value of a true inhibitor towards ACE will not be affected by preincubation. A real substrate shows a higher IC_50_ value after preincubation due to transformation of the original ACEI peptide into an inactive or less active fragment; while, a pro-drug type shows improved ACEI activity due to the formation of a more active fragment generated by ACE [15]. Since the reactivity of the identified substrates to ACE has been demonstrated using synthetic peptides, the inhibitory activity of their resulting peptide products is of interest in realizing the role of the identified substrates in ACE inhibition. In this study, six substrates with high to low D/H ratios were further selected to be pre-incubated with ACE, and then subjected to the ACE inhibition assay using hipppuryl-L-histidyl-L-Leucine (HHL) as the substrate to characterize their role as a real substrate or a prodrug according to the previous protocol [25]. The IC_50_ values with and without ACE pre-incubation for the six substrates are determined using a nonlinear regression between ACE inhibition (%) and the logarithm of inhibitor concentrations, as shown in Figure S40 in Supporting Information, and the result was summarized in Table 2. Among these six substrates, the IC_50_ values of AR-9, HW-10, and LL-8 were increased after ACE pre-incubation, which revealed that their products showed lower ACE inhibition activity, and they could be classified as real-substrate type inhibitors. In contrast, LL-13, FF-10, and YQ-7 showed lower IC_50_ values after pre-incubation, indicating that they are pro-drug type inhibitors.

### 3.5. The Substrates’ Protective Effects on ACE-Mediated Hydrolysis of Angiotensin I

**Figure 8:**
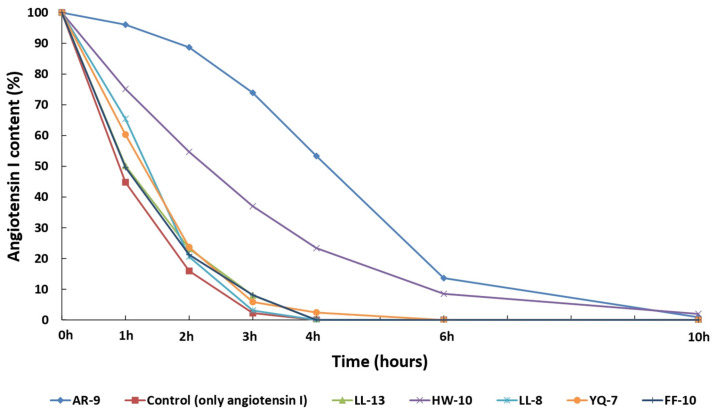
The protective effects of six exogenous substrates on ACE-mediated hydrolysis of angiotensin I. The Ang-I content was determined at different ACE hydrolysis times.

To evaluate the inhibitory effects of exogenous substrates against ACE mediated hydrolysis, six peptide substrates were co-incubated with the endogenous substrate Ang-I individually. The remaining abundance of synthetic Ang-I during the ACE hydrolysis was monitored using LC-MS, as shown in Figure S41. Without exogenous substrates, Ang-I was hydrolyzed almost completely within 3 h. Interestingly, 73.9% of Ang-I remained when it co-incubated with AR-9 for 3 h, which implied that AR-9 can protect Ang-I from ACE hydrolysis. The order of the protective effect of these peptides on Ang-I were AR-9 > HW-10 > FF-10 > LL-13 > YQ-7 > LL-8, as shown in Figure 8. These data indicated that the exogenous substrates could play the role of an inhibitor, which protects Ang-I from ACE hydrolysis. However, the exogenous substrates or inhibitors were screened in vitro in this study. Their in vivo activities against ACE should be further confirmed using animal studies.

## 4. Conclusions

In this study, an efficient and large-scale screening method of ACE’s exogenous substrates was demonstrated. Using this approach, the sequence consensus of ACE’s substrate was systematically explored and confirmed using synthetic peptides. Some of the exogenous substrates also showed ACE inhibitory activities which were further characterized as the substrate- or prodrug-type inhibitor. Moreover, the substrate AR-9 showed significant ACEI activity and protected Ang-I from ACE hydrolysis, which revealed its in vivo potential in ACE-targeted blood pressure management. In addition, we believe that this peptidomics approach is promising in the discovery of substrates or inhibitory peptides for other peptidases.

## Figures and Tables

**Table 1:** Identified substrates derived from milk hydrolysate.

Substrate	D/H Ratio	Product	Protein
AYFYPELFR	0.19	AYFYPEL	CASA1_BOVIN
FFVAPFPEVF	0.87	FFVAPFPE	
HQGLPQEVLNENLLR	0.76	HQGLPQEVLNENL	
VAPFPEVFG	0.83	VAPFPEV	
YVPLGTQ	0.33	YVPLG	
NAVPITPTLNR	0.61	NAVPITP	CASA2_BOVIN
		NAVPITPTL	
HQPHQPLPPTVM	0.40	HQPHQPLPPT	CASB_BOVIN
IPPLTQT	0.47	IPPLT	
MFPPQSVL	0.21	MFPPQS	
VVPPFLQPEVM	0.80	VVPPFLQPE	
YPVEPFTESQSL	0.76	YPVEPFTE	
		YPVEPFTESQ	
LVYPFPGPIHNSL	0.26	LVYPFPGPIHN	
INNQFLPYPYY	0.33	INNQFLPYP	CASK_BOVIN
SRYPSYGLN	0.81	SRYPSYG	
SAYPGQITSN	0.65	SAYPGQIT	TRY1_BOVIN
QLDAYPSGAW	0.47	QLDAYPSG	CASA1_BOVIN
FPQYLQY	0.12	FPQYL	CASA2_BOVIN
KVIPYVRY	0.66	KVIPYV	
FALPQYLK	0.94	FALPQY	
HLPLPLLQSW	0.12	HLPLPL	CASB_BOVIN
		HLPLPLLQ	
AVPYPQR	0.44	AVPYP	
DMPIQAF	0.55	DMPIQ	
DMPIQAFL	0.59	DMPIQA	
LHLPLPLL	0.74	LHLP	
		LHLPLP	
ALPMHIR	0.15	ALPMH	LACB_BOVIN
HPHPHLSF	0.15	HPHPHL	CASK_BOVIN
LRPVAAEIY	0.65	LRPVAAE	TRFL_BOVIN
TTMPLW	0.15	TTMP	CASA1_BOVIN
ALNEINQFY	0.84	ALNEINQ	CASA2_BOVIN
QVSLNSGY	0.56	QVSLNS	TRY1_BOVIN
SIVHPSY	0.18	SIVHP	

**Table 2:** ACE inhibitory (ACEI) effects and types for the six identified substrates.

Substrate	IC_50_ (μM) (Regular Assay)	IC_50_ (μM) (Pre-Incubation)	Type
AR-9	6.8 ± 0.23	25.3 ± 0.96	Real substrate
HW-10	10.6 ± 0.17	17.8 ± 0.65	
LL-8	12.3 ± 0.37	71.9 ± 2.19	
LL-13	38.93 ± 2.05	27.29 ± 2.16	Pro-drug
FF-10	104.4 ± 2.47	44.63 ± 3.06	
YQ-7	249.6 ± 7.9	189.3 ± 9.6	

## Data Availability

Not applicable.

## Supplementary Materials

The following supporting information can be downloaded at www.mdpi.com/article/10.3390/pharmaceutics15020425/s1, Figures S1–S30: The identification of other substrate candidates; Figures S31–S35: The confirmation of the other five substrates using synthetic peptides; Figure S36: The reactivity confirmation of NIPPLTQTPV (Pro at P1′ position); Figure S37: The reactivity confirmation of IASGEPTSTPTTE (Glu at P2′ position); Figure S38: The reactivity confirmation of LYQGPIVL (Ile at P1 position); Figure S39: The reactivity confirmation of VRSPAQILQ (Ile at P1 position); Figure S40: IC_50_ values of six peptides with and without ACE pre-incubation; Figure S41: The remaining abundance of synthetic Ang-I during the ACE hydrolysis monitored using LC-MS at various pre-incubation times.
