# Supplementary material for: An Efficient Peptidomics Screening for Exogenous Substrates and Inhibitory Peptides of the Dipeptidase ACE from Milk Hydrolysate
Source: Pharmaceutics. 2023 Jan 27;15(2):425. doi: 10.3390/pharmaceutics15020425 (PMC9965520; doi:10.3390/pharmaceutics15020425)
Supplement: Supplementary file 1 [file pharmaceutics-15-00425-s001.zip › pharmaceutics-2107318-supplementary.pdf]

Supporting Information

Figure S1

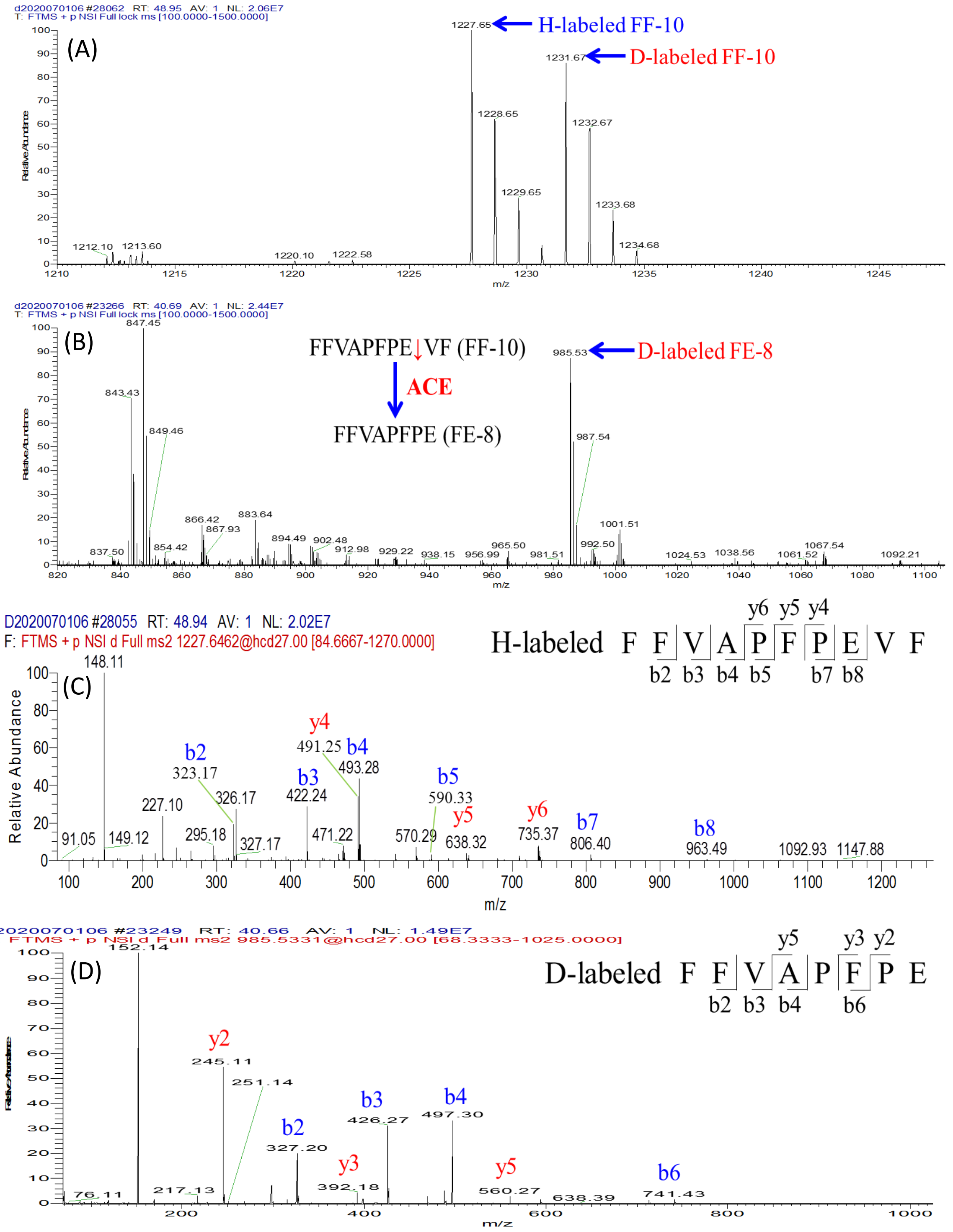

Figure S1. LC-MS/MS identification of ACE exogenous substrate FFVAPFPEVF (FF-10) and its product. (A) MS spectrum of D- and H-labeled FF-10; (B) MS spectrum of the product FFVAPFPE (FE-8); (C) MS/MS spectrum of FF-10 (m/z 1227.6); (D) MS/MS of FE-8 (m/z 985.5).

Figure S2.

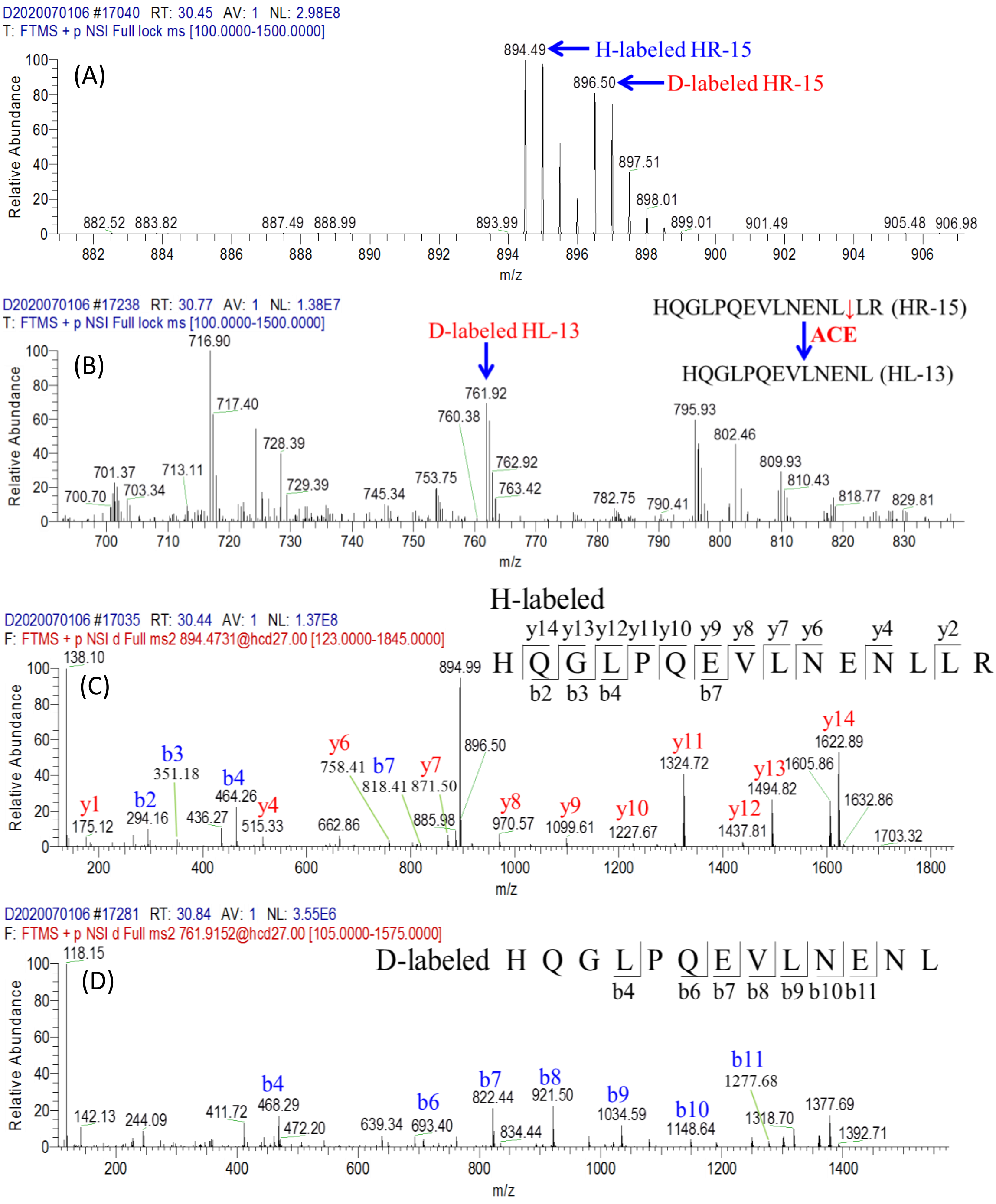

Figure S2. LC-MS/MS identification of ACE exogenous substrate HQGLPQEVLNENLLR (HR-15) and its product. (A) MS spectrum of D- and H-labeled HR-15; (B) MS spectrum of the product HQGLPQEVLNENL (HL-13); (C) MS/MS spectrum of HR-15 (m/z 894.4); (D) MS/MS of HL-13 (m/z 761.9).

Figure S3

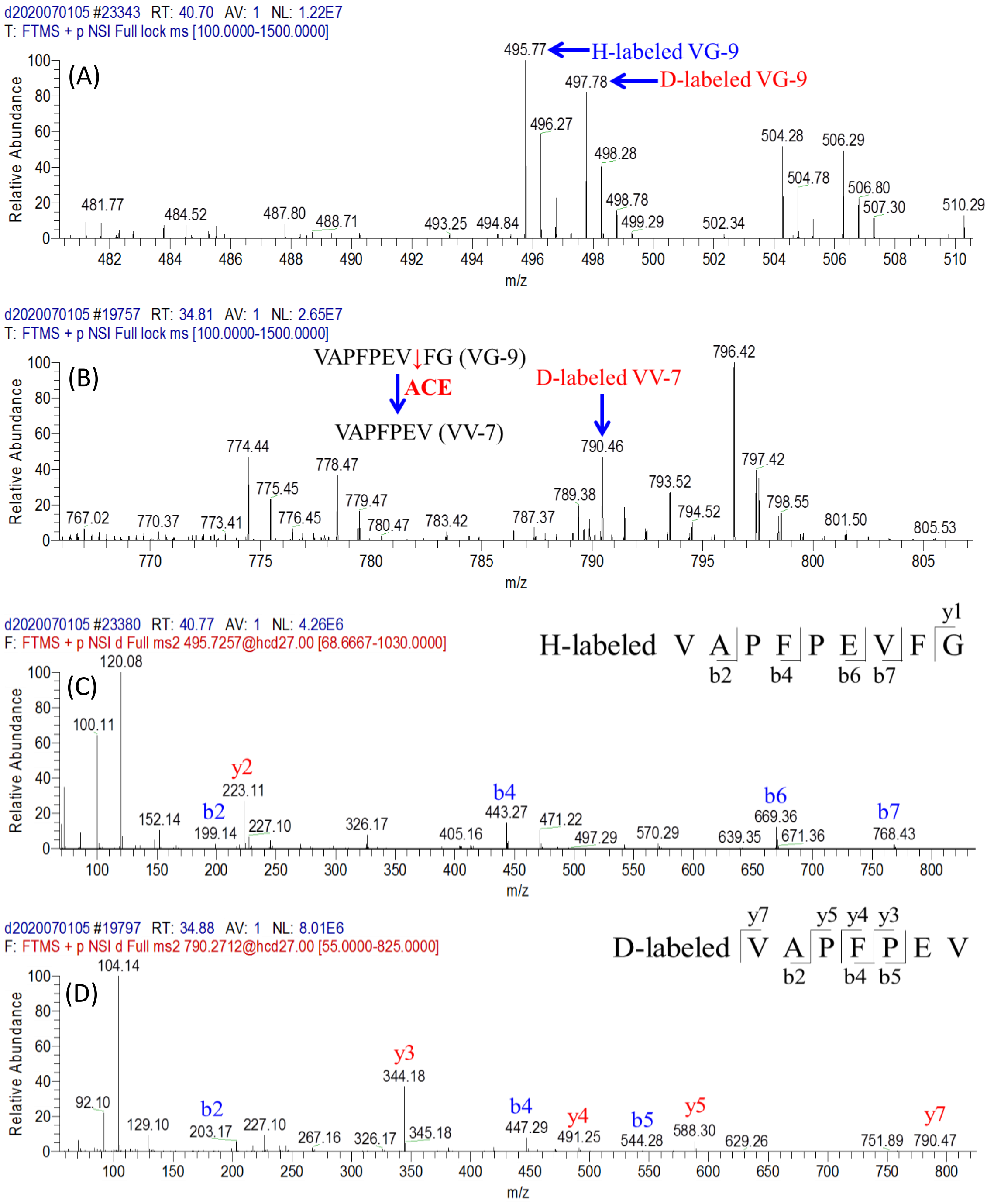

Figure S3. LC-MS/MS identification of ACE exogenous substrate VAPFPEVFG (VG-9) and its product. (A) MS spectrum of D- and H-labeled VG-9; (B) MS spectrum of the product VAPFPEV (VV-7); (C) MS/MS spectrum of VG-9 (m/z 495.7, +2); (D) MS/MS of VV-7 (m/z 790.2).

Figure S4

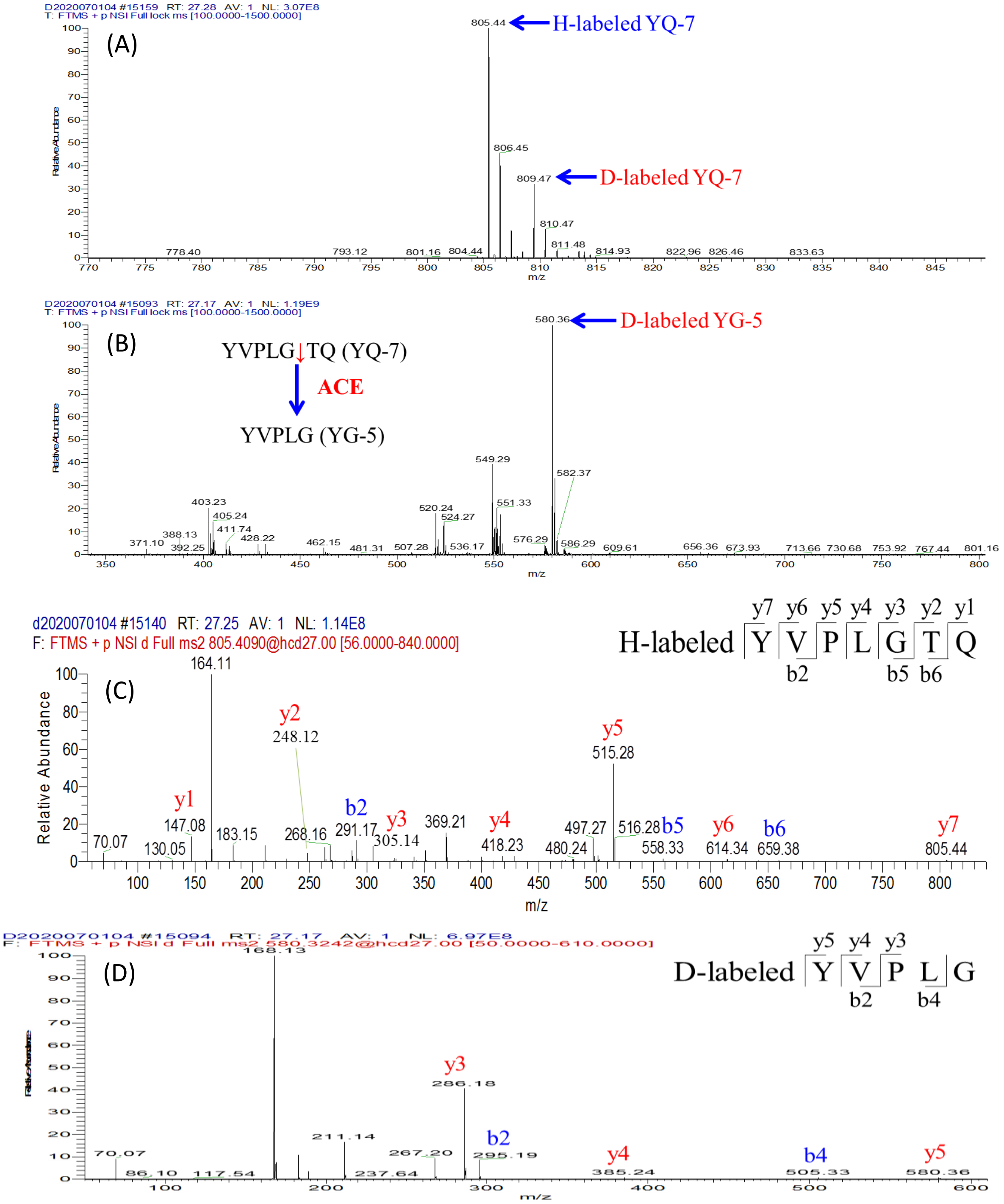

Figure S4. LC-MS/MS identification of ACE exogenous substrate YVPLGTQ (YQ-7) and its product. (A) MS spectrum of D- and H-labeled YQ-7; (B) MS spectrum of the product YVPLG (YG-5); (C) MS/MS spectrum of YQ-7 (m/z 805.4); (D) MS/MS of YG-5 (m/z 580.3).

Figure S5-1

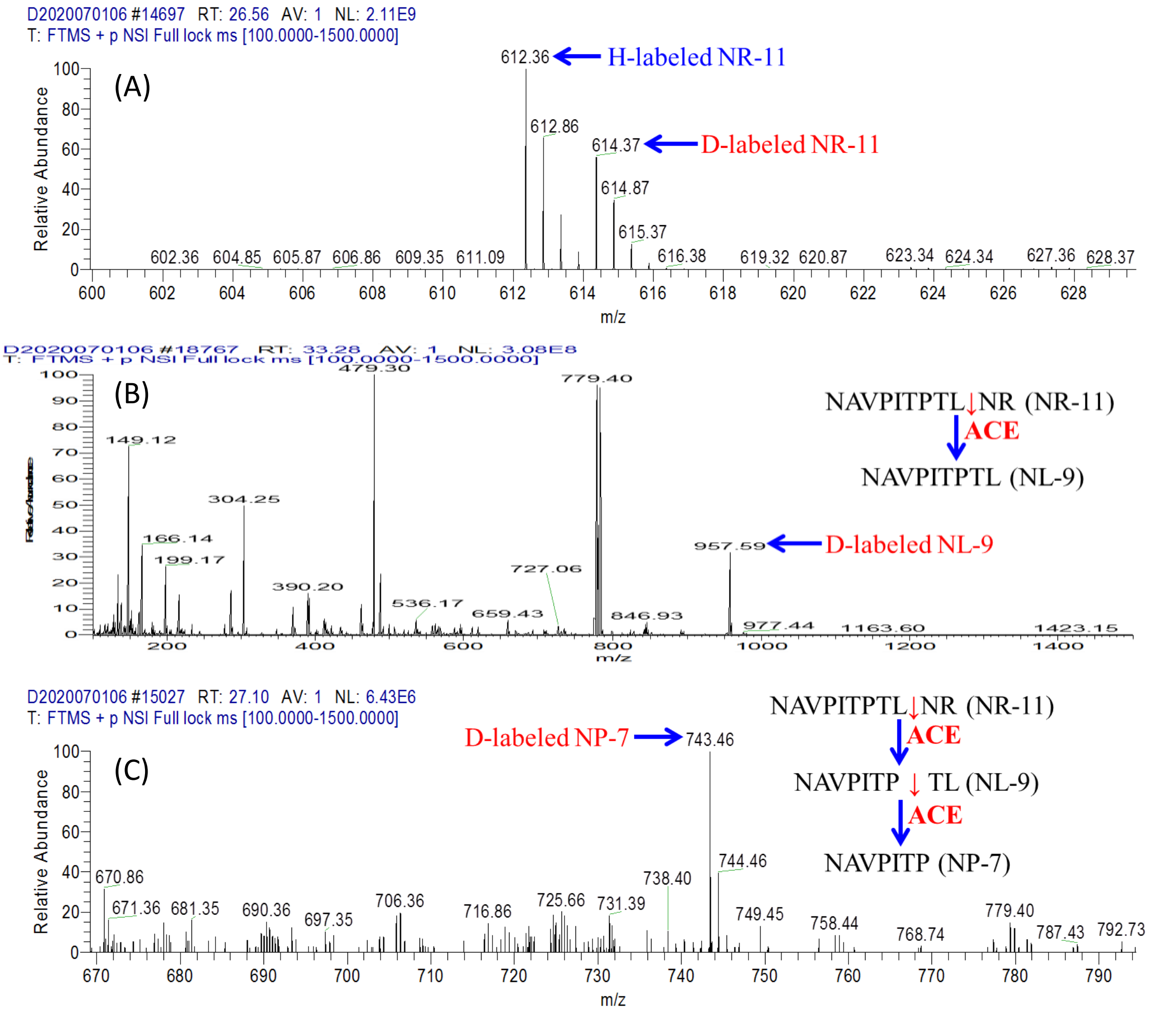

Figure S5-1. LC-MS/MS identification of ACE exogenous substrate NAVPITPTLNR (NR-11) and its product. (A) MS spectrum of D- and H-labeled NR-11; (B) MS spectrum of the product NAVPITPTL (NL-9); (C) MS spectrum of NAVPITP (NP-7).

Figure S5-2

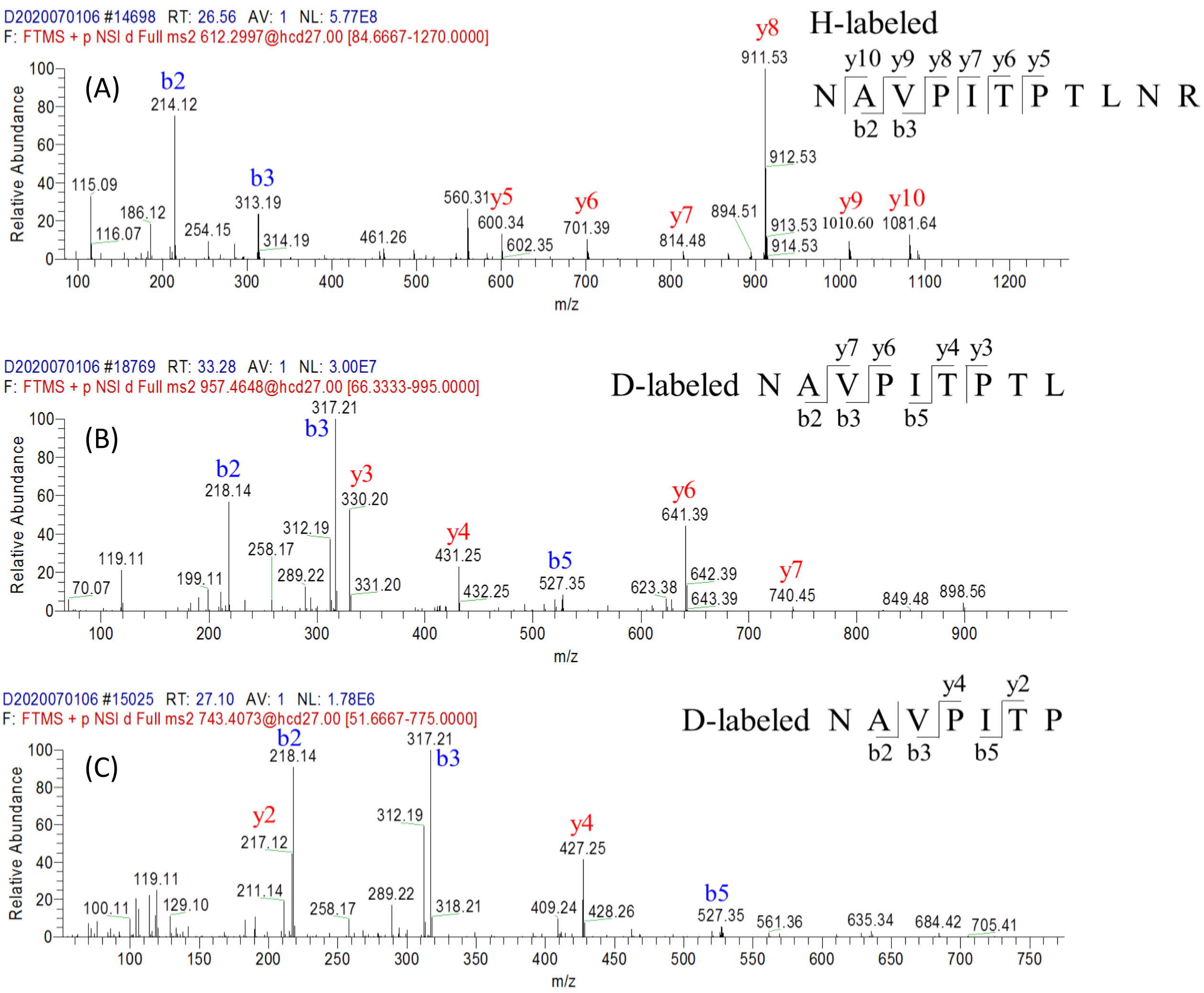

Figure S5-2. LC-MS/MS identification of ACE exogenous substrate NAVPITPTLNR (NR-11) and its product. (A) MS/MS spectrum of H-labeled NR-11 (m/z 612.3); (B) MS/MS spectrum of the product NL-9 (m/z 957.5); (C) MS/MS spectrum of NP-7 (m/z 743.4).

Figure S6

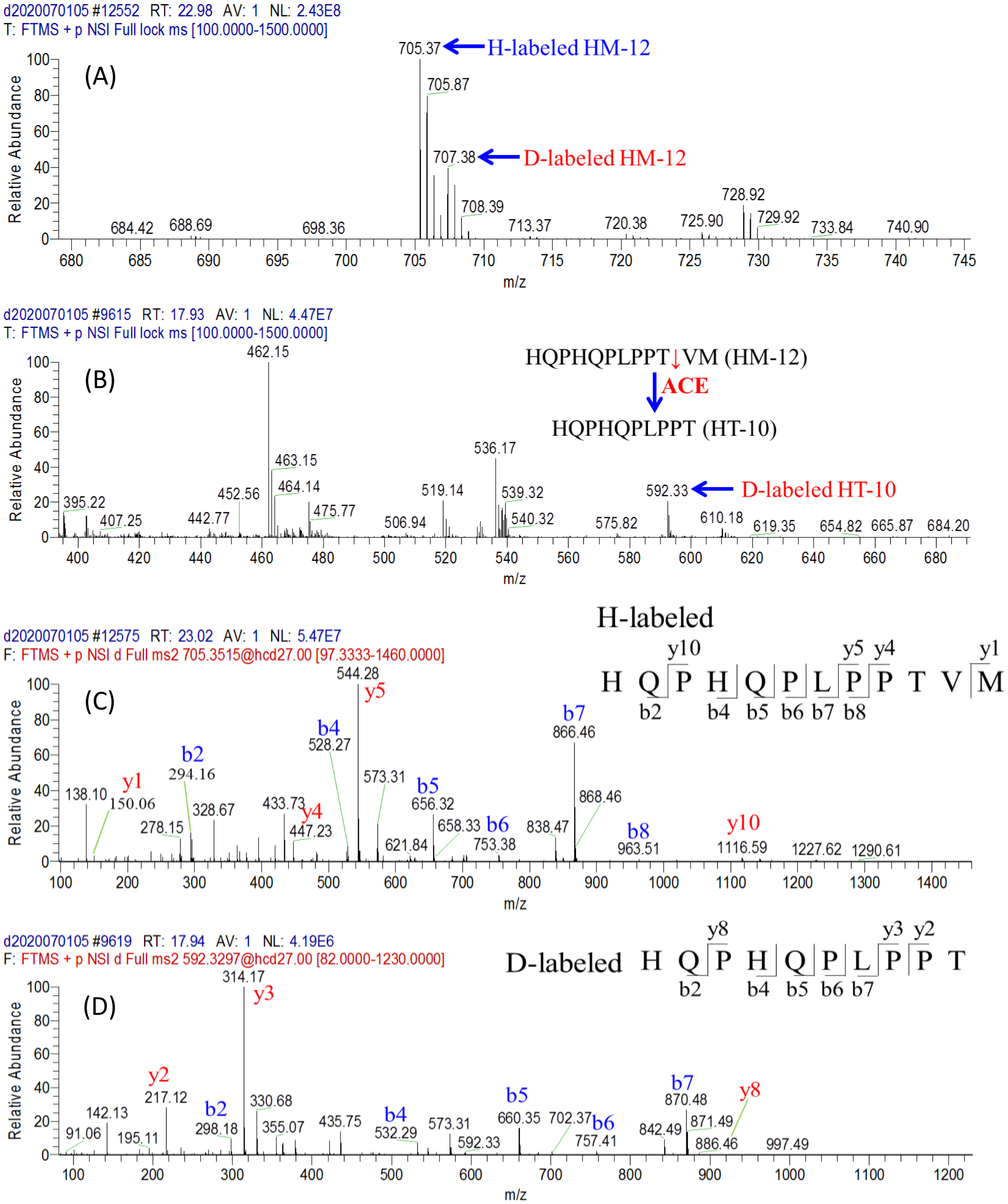

Figure S6. LC-MS/MS identification of ACE exogenous substrate HQPHQPLPPTVM (HM-12) and its product. (A) MS spectrum of D- and H-labeled HM-12; (B) MS spectrum of the product HQPHQPLPPT (HT-10); (C) MS/MS spectrum of HM-12 (m/z 705.3); (D) MS/MS of HT-10 (m/z 592.3).

Figure S7

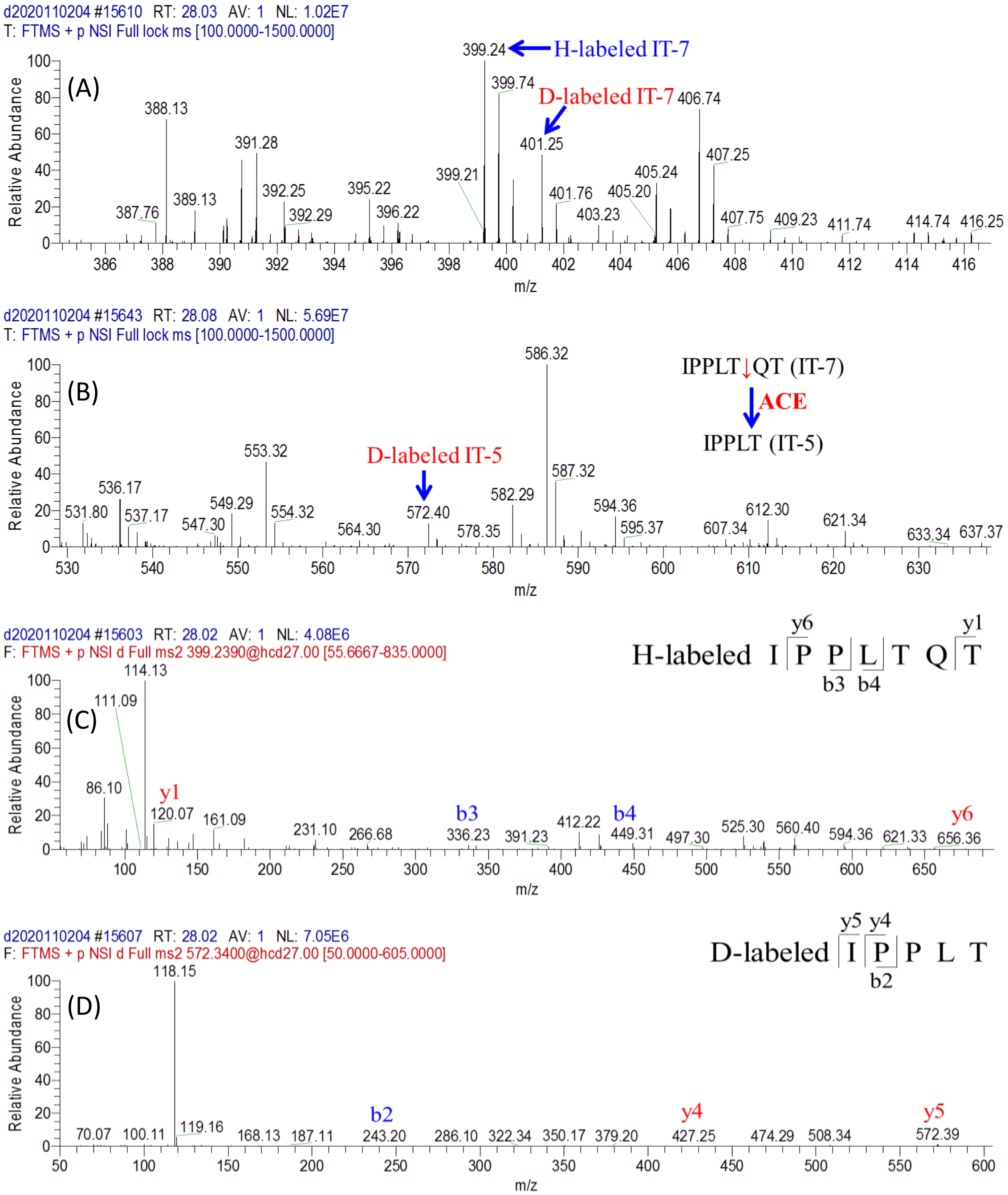

Figure S7. LC-MS/MS identification of ACE exogenous substrate IPPLTQT (IT-7) and its product. (A) MS spectrum of D- and H-labeled IT-7; (B) MS spectrum of the product IPPLT (IT-5); (C) MS/MS spectrum of IT-7 (m/z 399.2); (D) MS/MS of HT-5 (m/z 572.3).

Figure S8

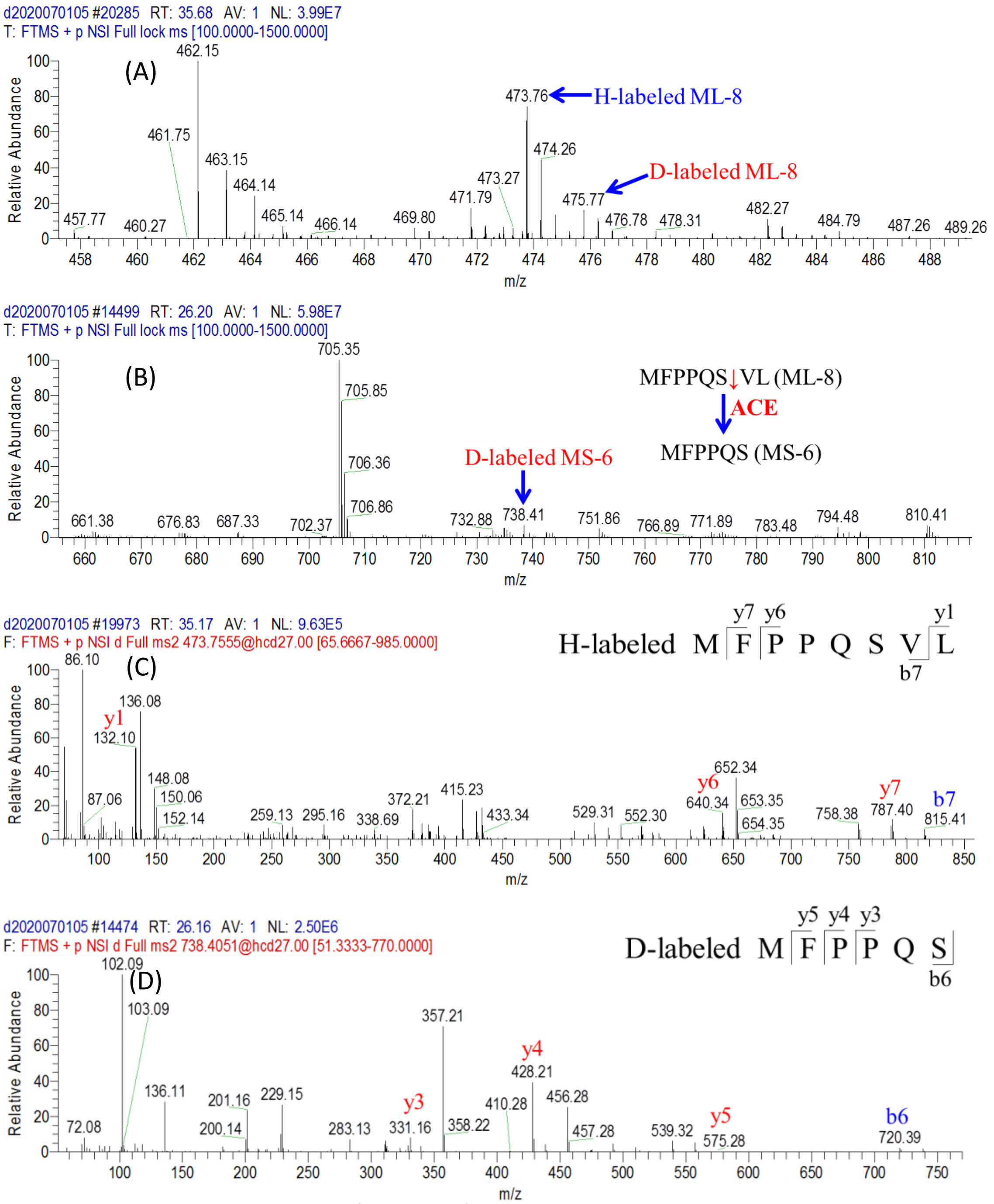

Figure S8. LC-MS/MS identification of ACE exogenous substrate MFPPQSVL (ML-8) and its product. (A) MS spectrum of D- and H-labeled ML-8; (B) MS spectrum of the product MFPPQS (MS-6); (C) MS/MS spectrum of ML-8 (m/z 473.7, +2); (D) MS/MS of MS-6 (m/z 738.4).

Figure S9

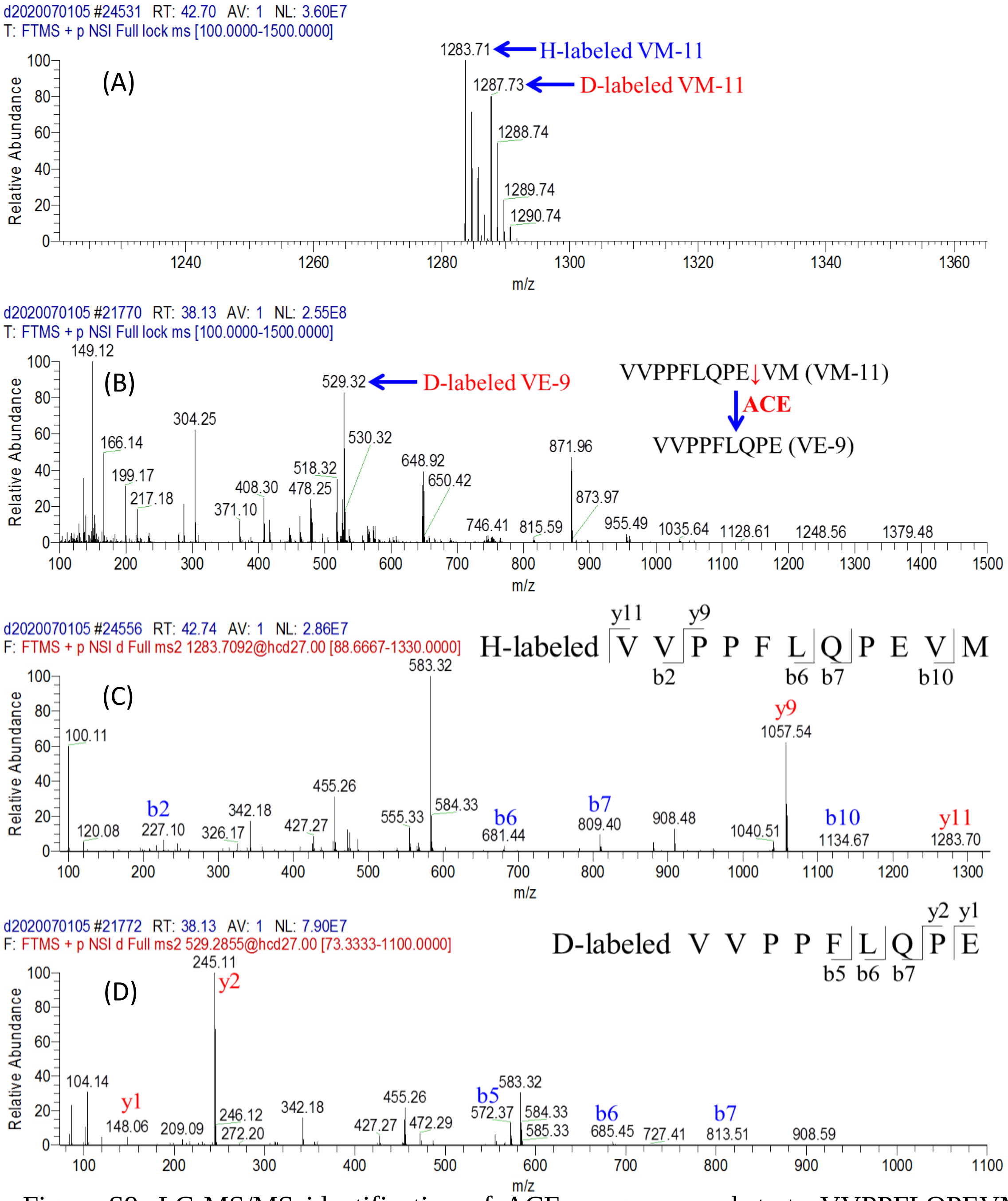

Figure S9. LC-MS/MS identification of ACE exogenous substrate VVPPFLQPEVM (VM-11) and its product. (A) MS spectrum of D- and H-labeled VM-11; (B) MS spectrum of the product VVPPFLQPE (VE-9); (C) MS/MS spectrum of VM-11 (m/z 1283.7); (D) MS/MS of VE-9 (m/z 529.2).

Figure S10-1

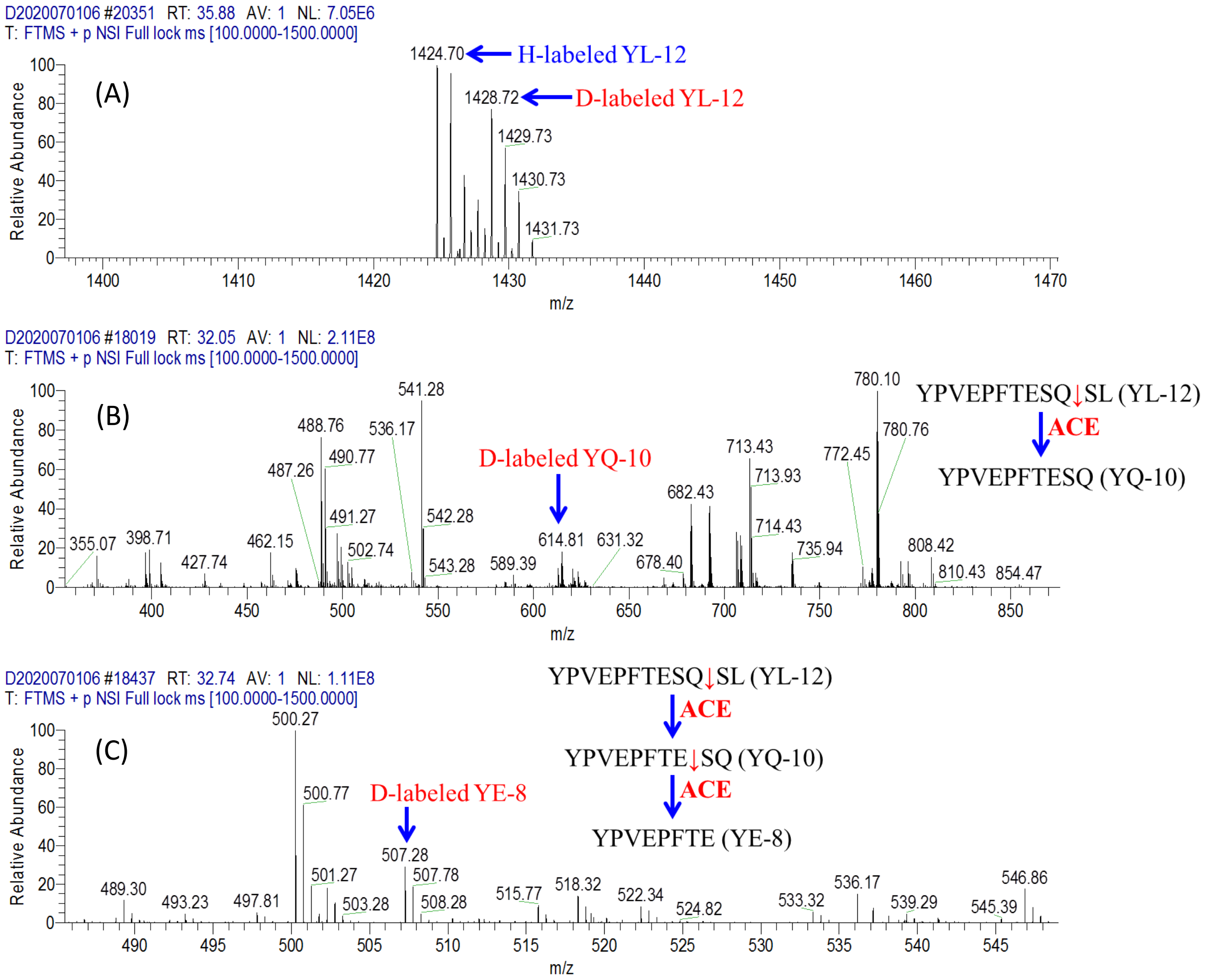

Figure S10-1. LC-MS/MS identification of ACE exogenous substrate YPVEPFTESQSL (YL-12) and its product. (A) MS spectrum of D- and H-labeled YL-12; (B) MS spectrum of the product YPVEPFTESQ (YQ-10); (C) MS spectrum of YPVEPFTE (YE-8).

Figure 10S-2

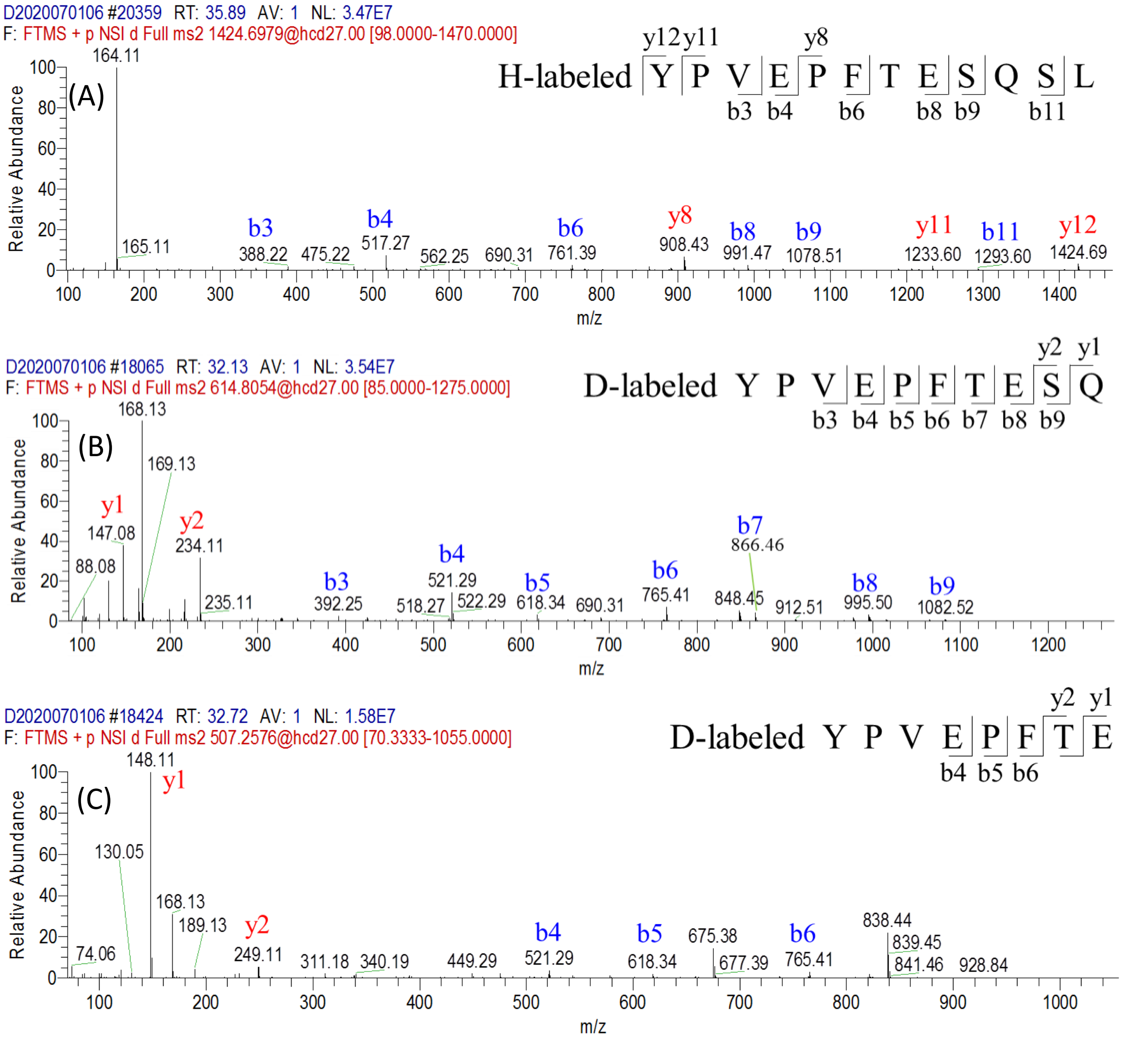

Figure S10-2. LC-MS/MS identification of ACE exogenous substrate YPVEPFTESQSL (YL-12) and its product. (A) MS/MS spectrum of YL-12 (m/z 1424.7); (B) MS/MS spectrum of the product YQ-10 (m/z 614.8); (C) MS/MS spectrum of YE-8 (m/z 507.2).

Figure S11

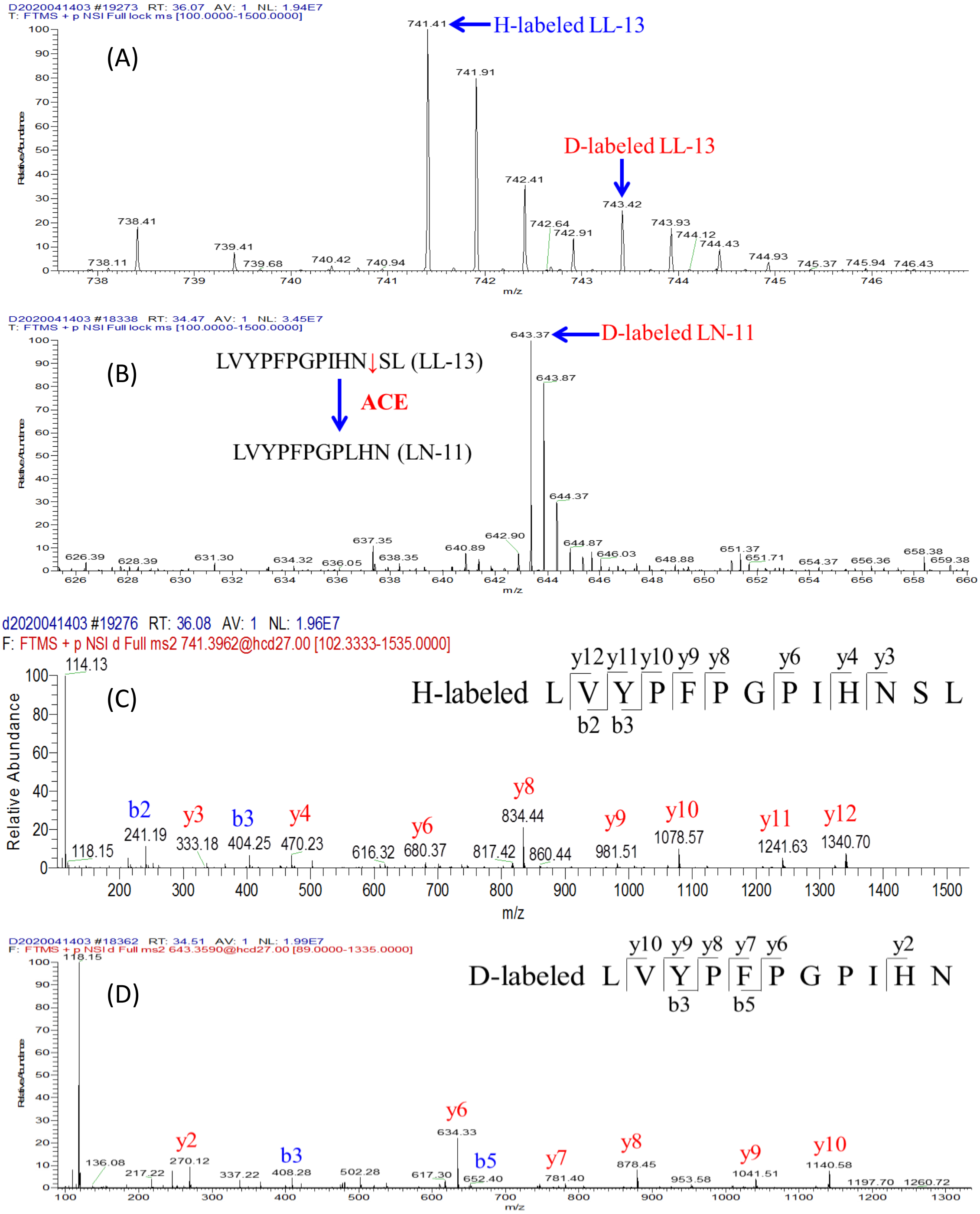

Figure S11. LC-MS/MS identification of ACE exogenous substrate LVYPFPGPIHNSL (LL-13) and its product. (A) MS spectrum of D- and H-labeled LL-13; (B) MS spectrum of the product LVYPFPGPIHN (LN-11); (C) MS/MS spectrum of LL-13 (m/z 741.4); (D) MS/MS of LN-11 (m/z 643.3).

Figure S12

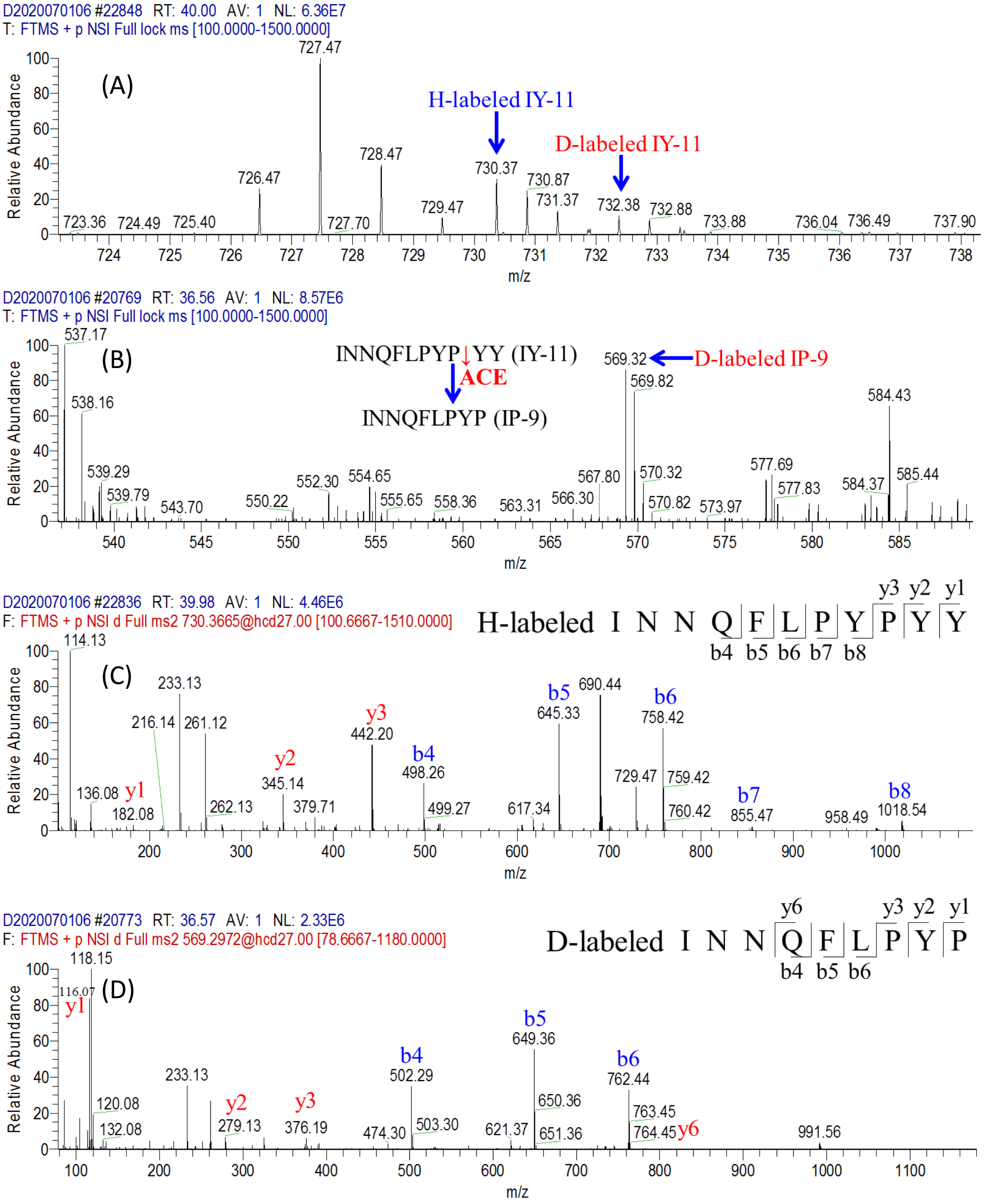

Figure S12. LC-MS/MS identification of ACE exogenous substrate INNQFLPYPYY (IY-11) and its product. (A) MS spectrum of D- and H-labeled IY-11; (B) MS spectrum of the product INNQFLPYP (IP-9); (C) MS/MS spectrum of IY-11 (m/z 730.3); (D) MS/MS of IP-9 (m/z 569.3).

Figure S13

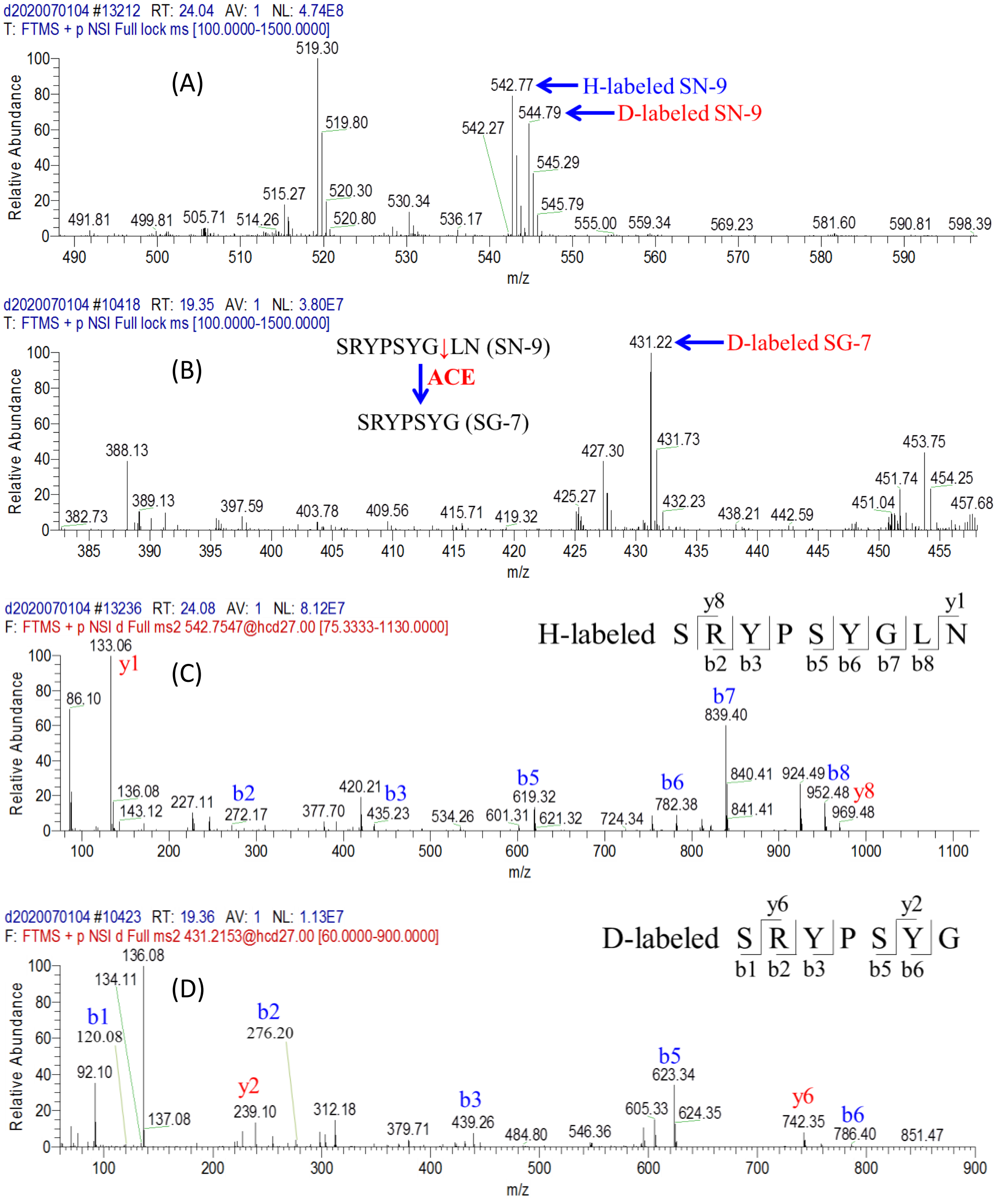

Figure S13. LC-MS/MS identification of ACE exogenous substrate SRYPSYGLN (SN-9) and its product. (A) MS spectrum of D- and H-labeled SN-9; (B) MS spectrum of the product SRYPSYG (SG-7); (C) MS/MS spectrum of SN-9 (m/z 542.7); (D) MS/MS of SG-7 (m/z 431.2).

Figure S14

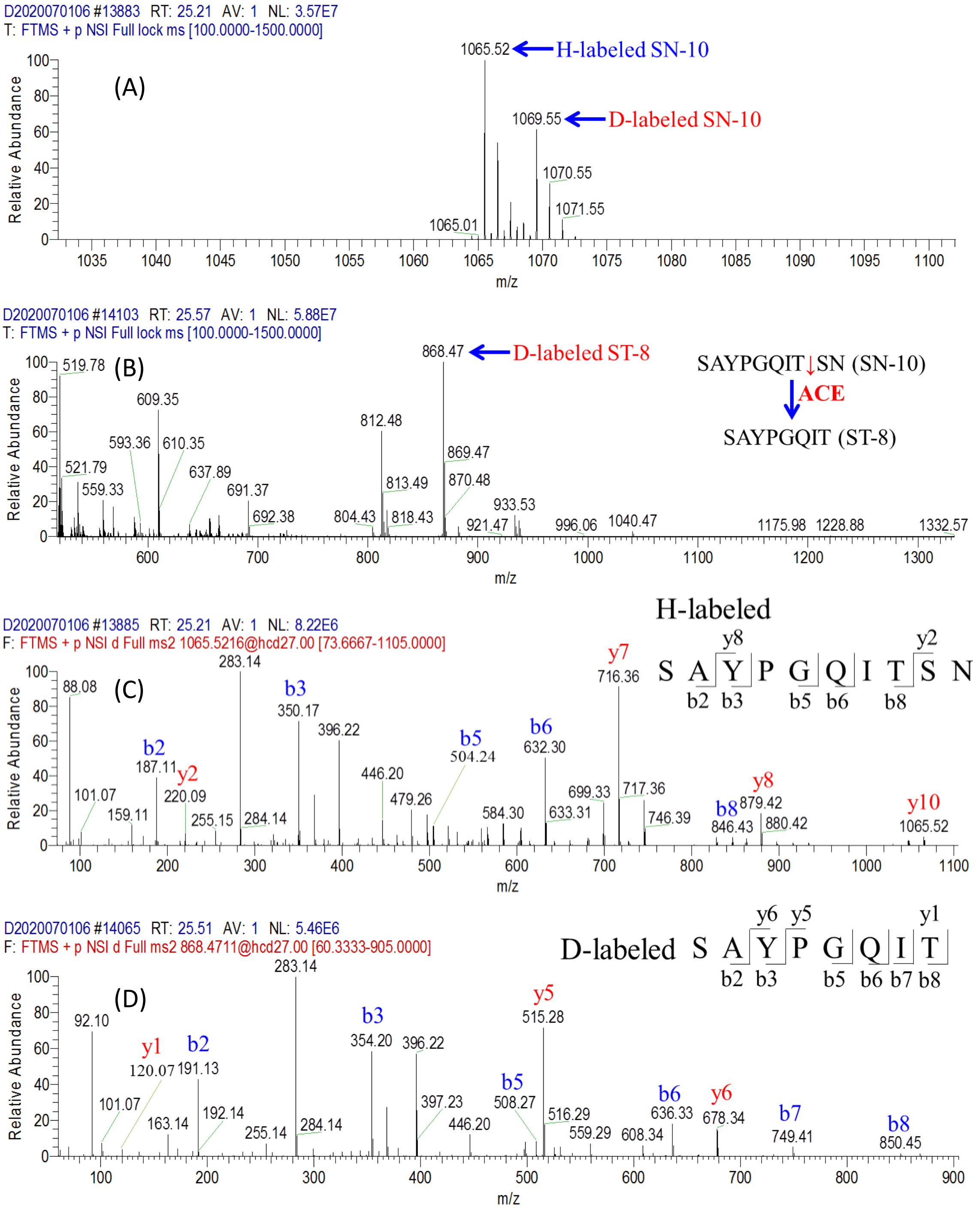

Figure S14. LC-MS/MS identification of ACE exogenous substrate SAYPGQITSN (SN-10) and its product. (A) MS spectrum of D- and H-labeled SN-10; (B) MS spectrum of the product SAYPGQIT (ST-8); (C) MS/MS spectrum of SN-10 (m/z 1065.5); (D) MS/MS of ST-8 (m/z 868.4).

Figure S15

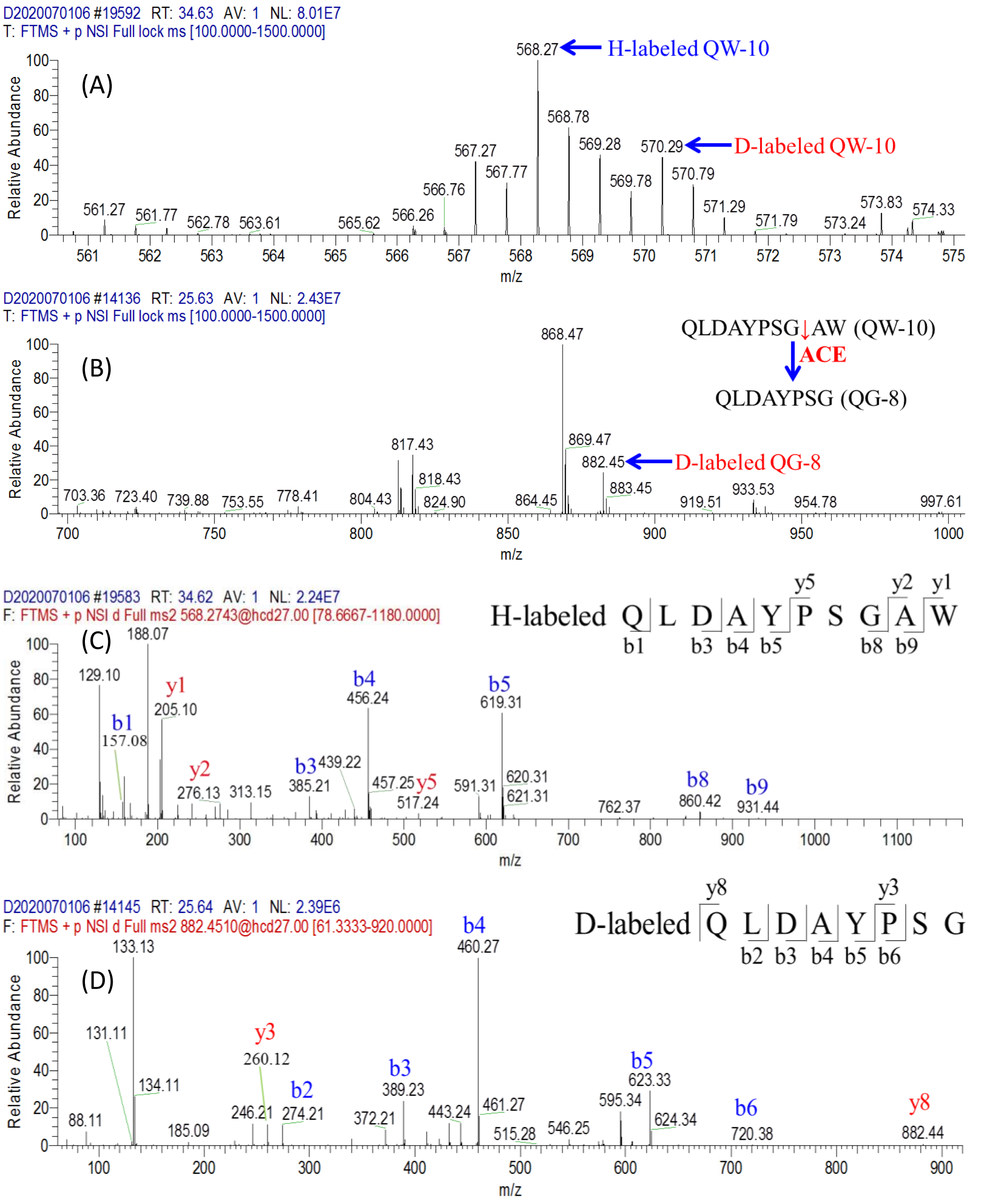

Figure S15. LC-MS/MS identification of ACE exogenous substrate QLDAYPSGAW (QW-10) and its product. (A) MS spectrum of D- and H-labeled QW-10; (B) MS spectrum of the product QLDAYPSG (QG-8); (C) MS/MS spectrum of QW-10 (m/z 568.2); (D) MS/MS of QG-8 (m/z 882.4).

Figure S16

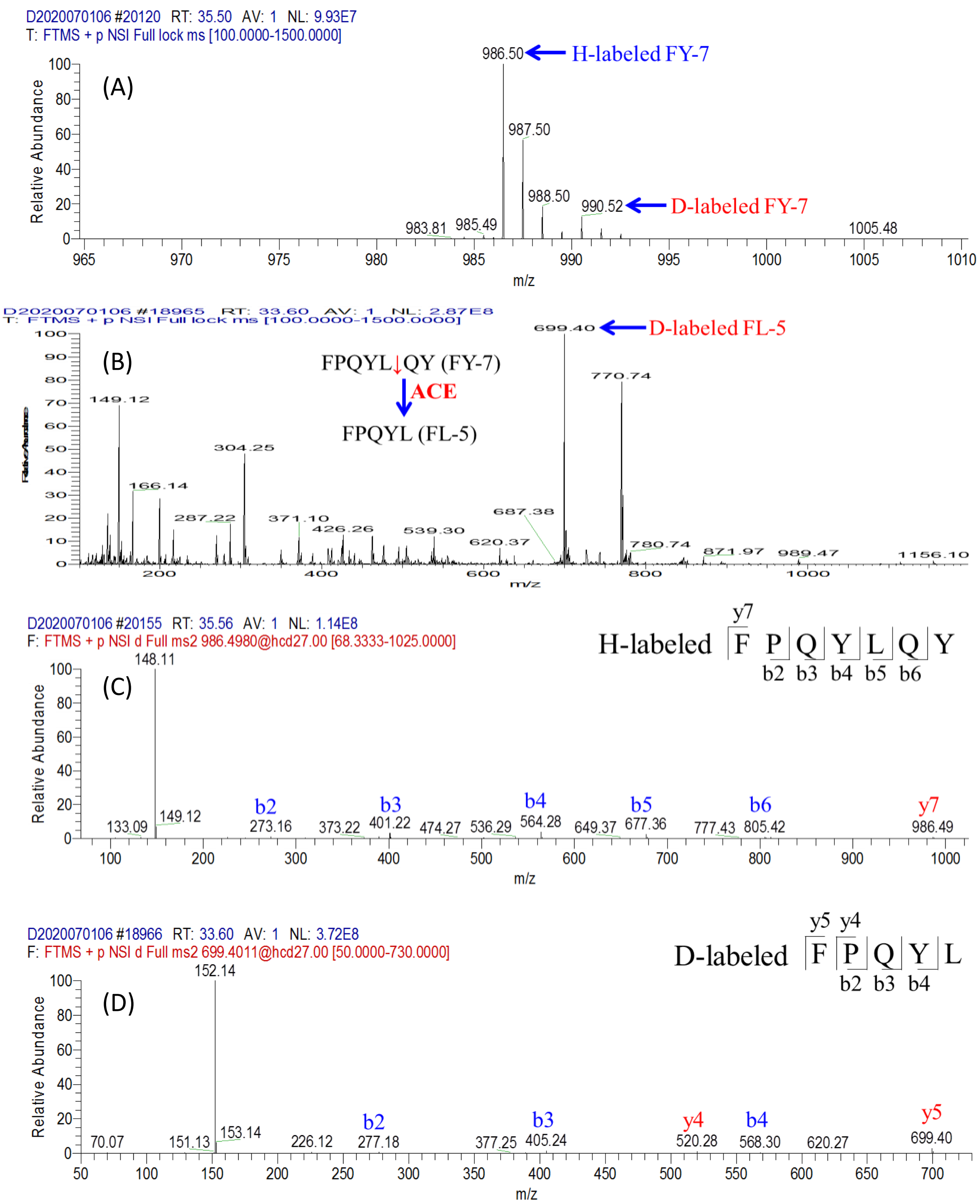

Figure S16. LC-MS/MS identification of ACE exogenous substrate FPQYLQY (FY-7) and its product. (A) MS spectrum of D- and H-labeled FY-7; (B) MS spectrum of the product FPQYL (FL-5); (C) MS/MS spectrum of FY-7 (m/z 986.4); (D) MS/MS of FL-5 (m/z 699.4).

Figure S17

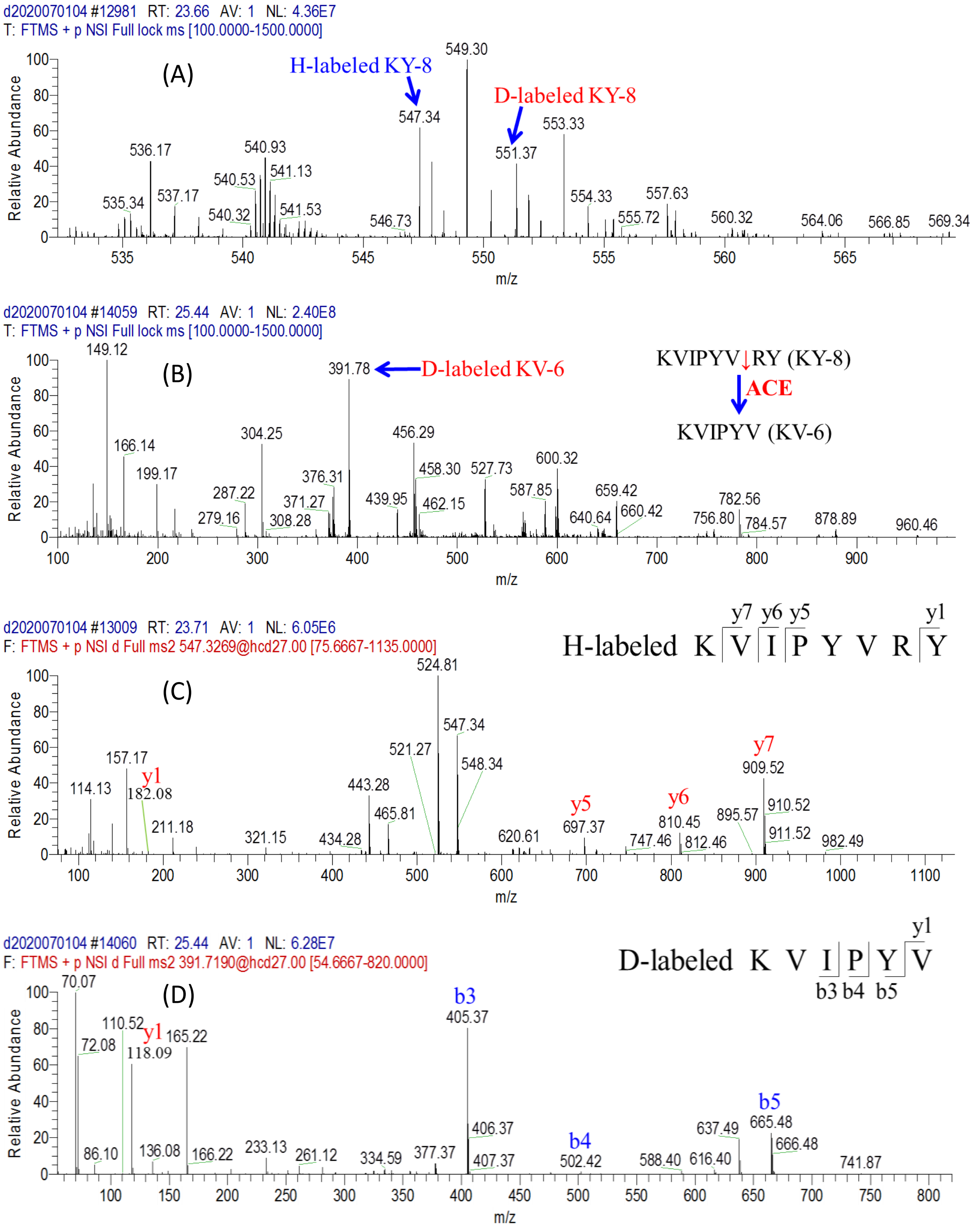

Figure S17. LC-MS/MS identification of ACE exogenous substrate KVIPYVRY (KY-8) and its product. (A) MS spectrum of D- and H-labeled KY-8; (B) MS spectrum of the product KVIPYV (KV-6); (C) MS/MS spectrum of FY-8 (m/z 547.3); (D) MS/MS of KV-6 (m/z 391.7).

Figure S18

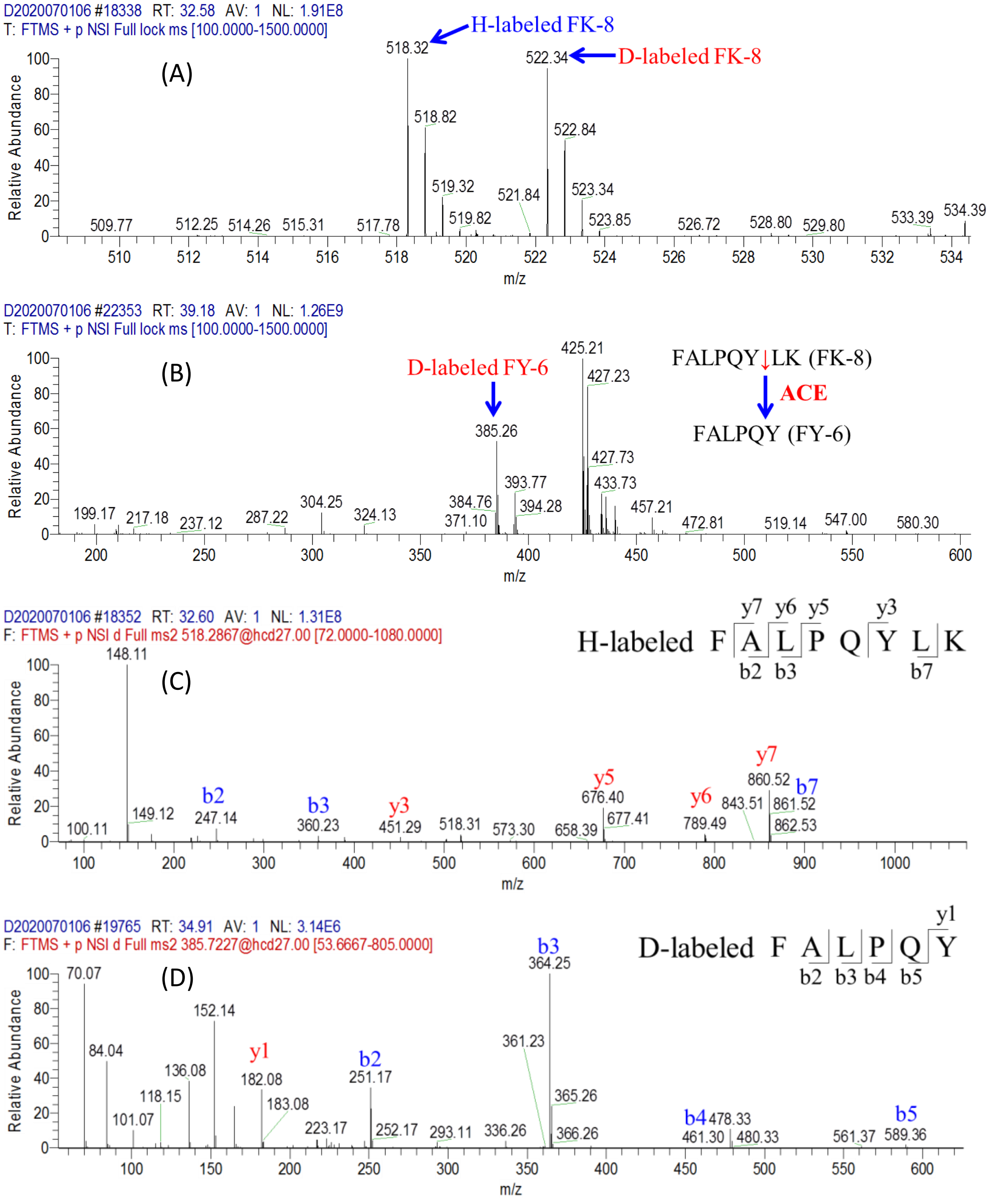

Figure S18. LC-MS/MS identification of ACE exogenous substrate FALPQYLK (FK-8) and its product. (A) MS spectrum of D- and H-labeled FK-8; (B) MS spectrum of the product FALPQY (FY-6); (C) MS/MS spectrum of FK-8 (m/z 518.2); (D) MS/MS of FY-6 (m/z 385.7).

Figure S19-1

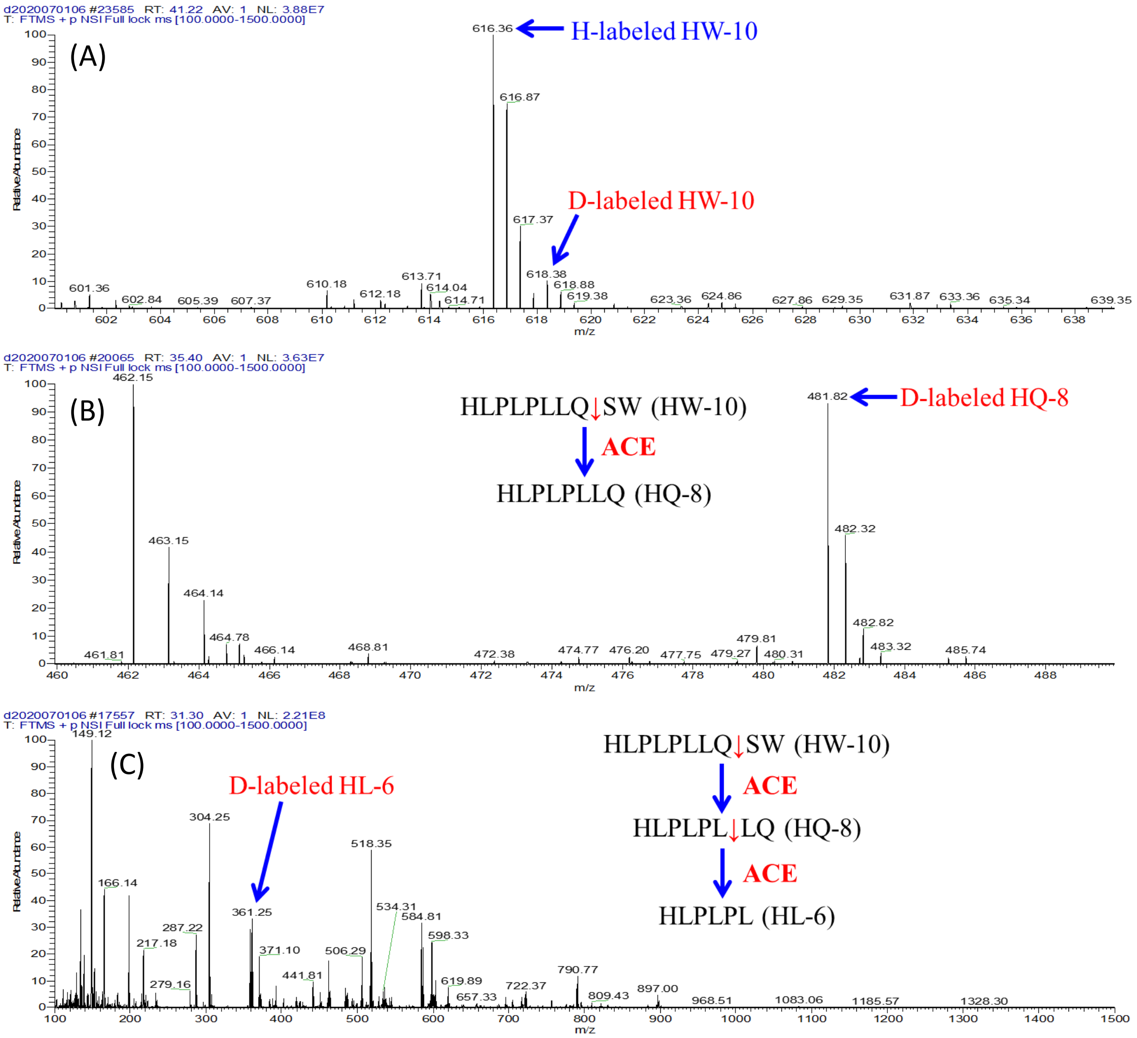

Figure S19-1. LC-MS/MS identification of ACE exogenous substrate HLPLPLLQSW (HW-10) and its product. (A) MS spectrum of D- and H-labeled HW-10; (B) MS spectrum of the product HLPLPLLQ (HQ-8); (C) MS spectrum of HLPLPL (HL-6).

Figure S19-2

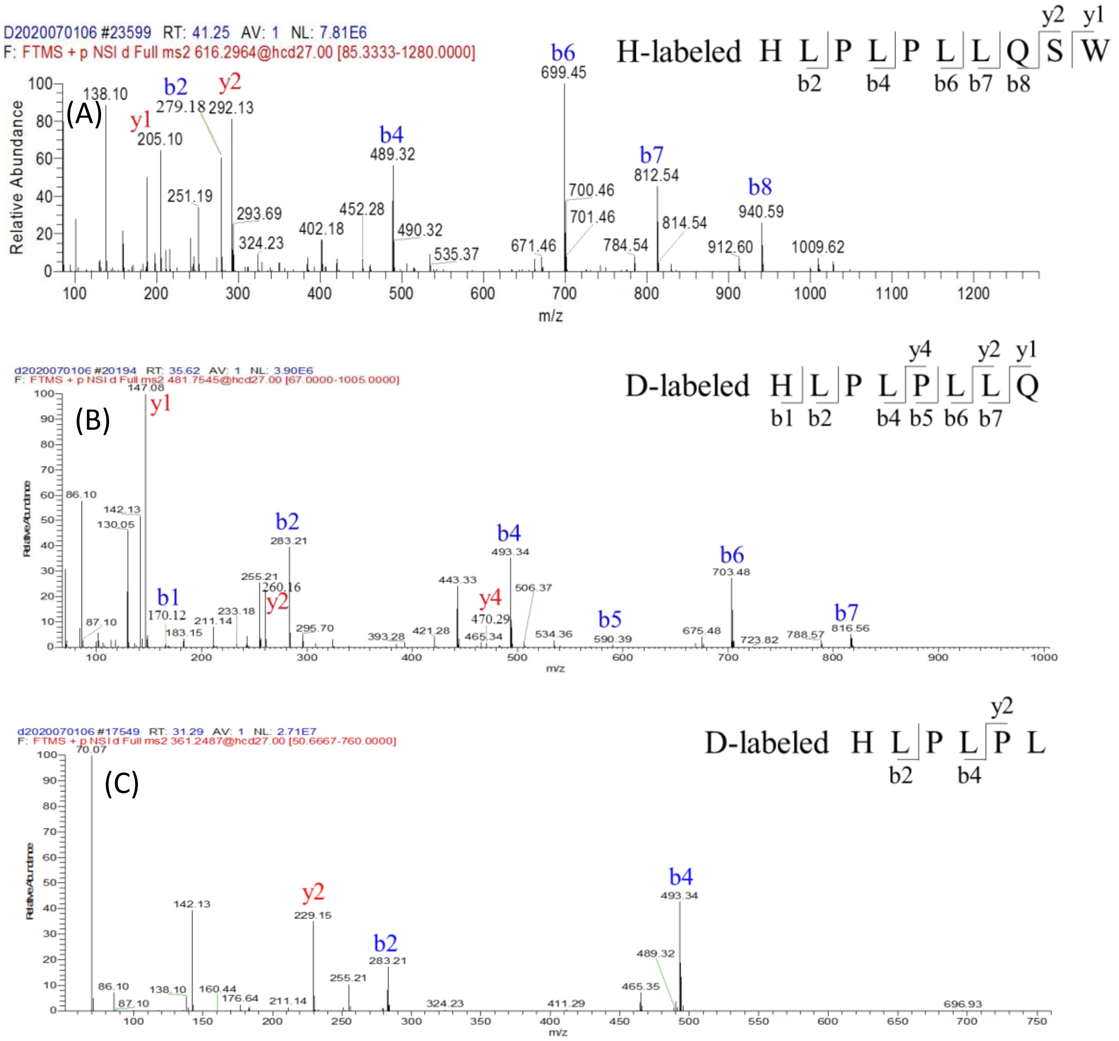

Figure S19-2. LC-MS/MS identification of ACE exogenous substrate HLPLPLLQSW (HW-10) and its products. (A) MS spectrum of H-labeled HW-10 (m/z 616.2); (B) MS/MS spectrum of the product HQ-8 (m/z 481.7); (C) MS/MS spectrum of HL-6 (m/z 361.2).

Figure S20

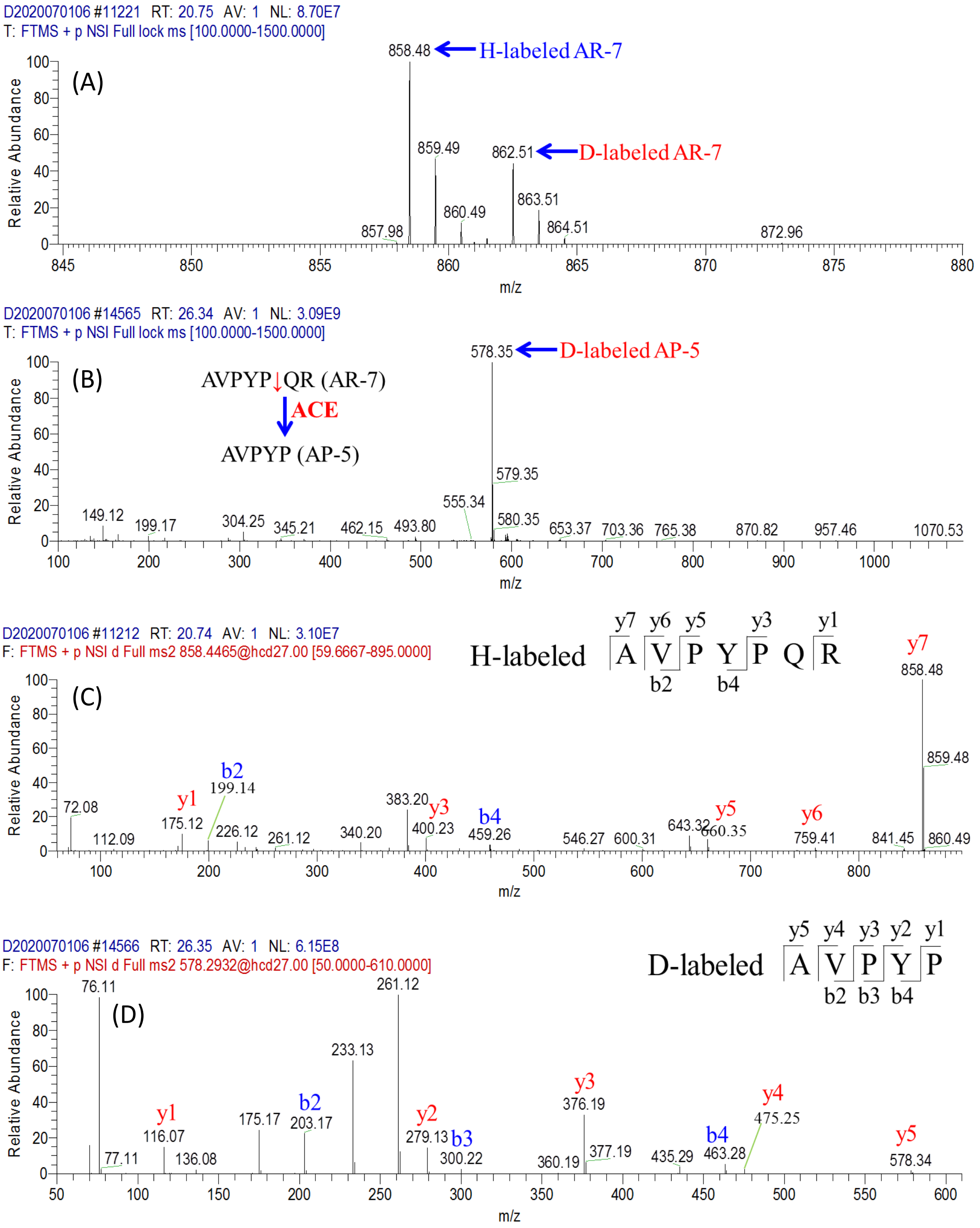

Figure S20. LC-MS/MS identification of ACE exogenous substrate AVPYPQR (AR-7) and its product. (A) MS spectrum of D- and H-labeled AR-7; (B) MS spectrum of the product AVPYP (AP-5); (C) MS/MS spectrum of AR-7 (m/z 858.4); (D) MS/MS of AP-5 (m/z 578.2).

Figure S21

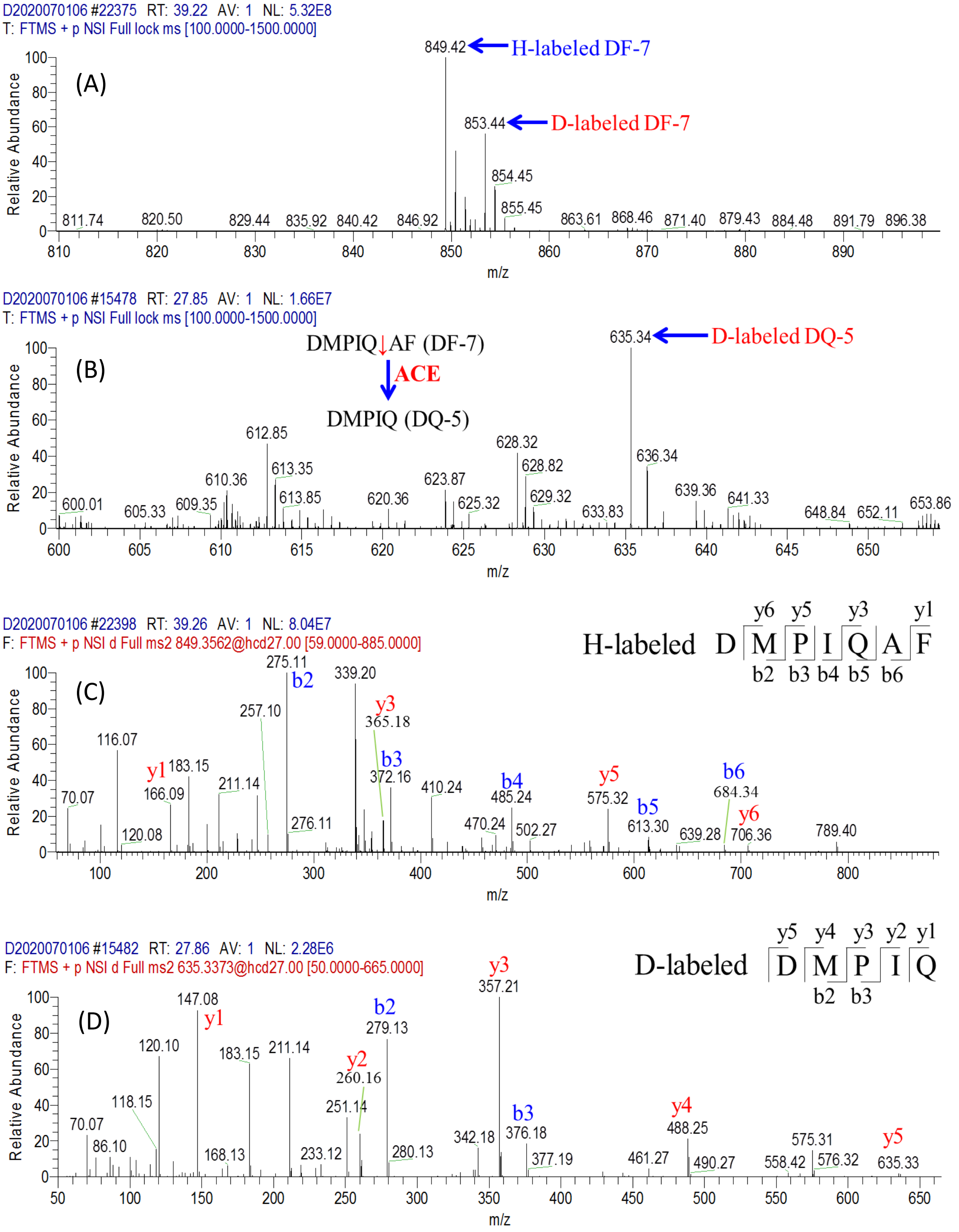

Figure S21. LC-MS/MS identification of ACE exogenous substrate DMPIQAF (DF-7) and its product. (A) MS spectrum of D- and H-labeled DF-7; (B) MS spectrum of the product DMPIQ (DQ-5); (C) MS/MS spectrum of DF-7 (m/z 849.3); (D) MS/MS of DQ-5 (m/z 635.3).

Figure S22

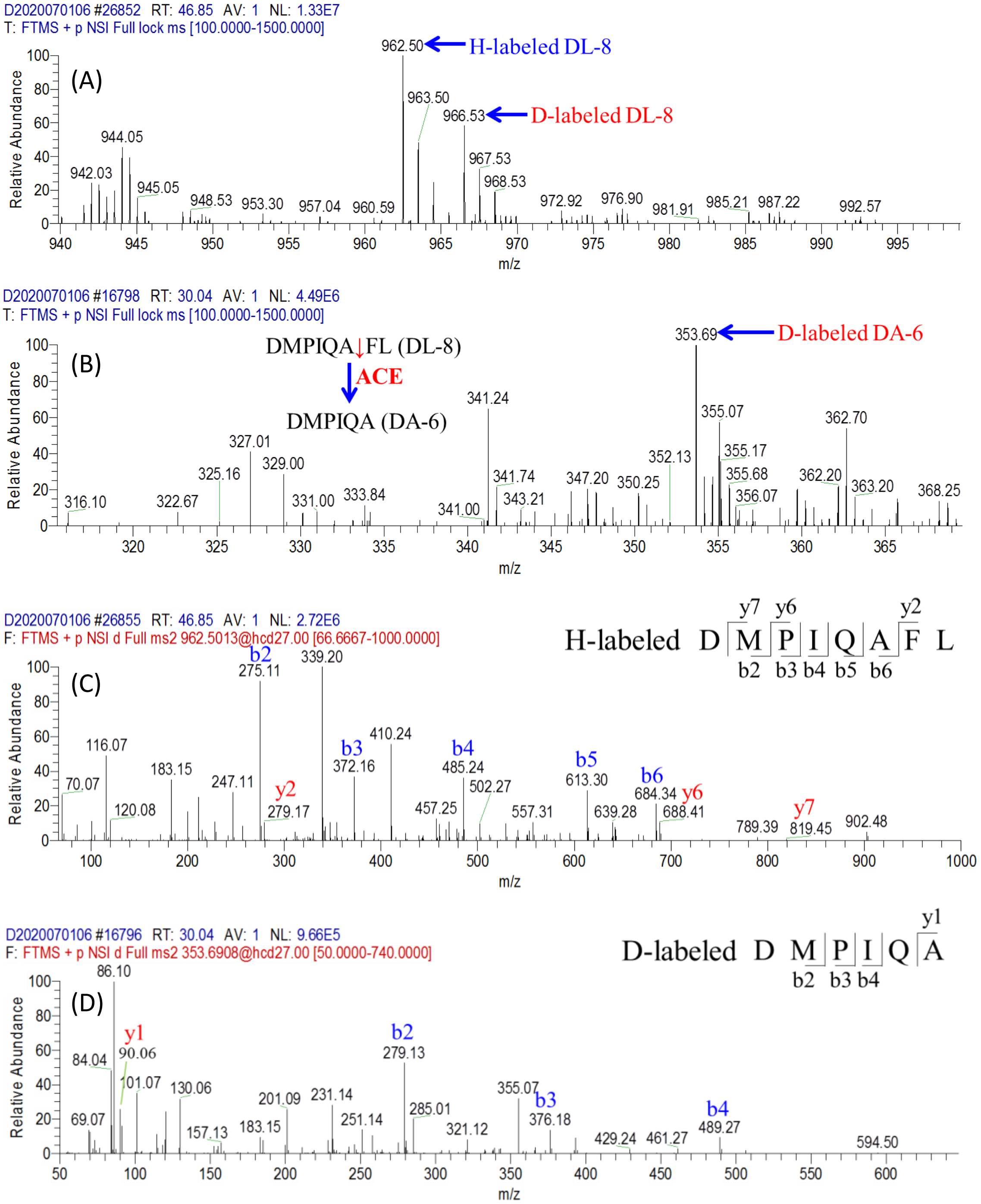

Figure S22. LC-MS/MS identification of ACE exogenous substrate DMPIQAFL (DL-8) and its product. (A) MS spectrum of D- and H-labeled DL-8; (B) MS spectrum of the product DMPIQA (DA-6); (C) MS/MS spectrum of DL-8 (m/z 962.5); (D) MS/MS of DA-6 (m/z 353.6).

Figure S23-1

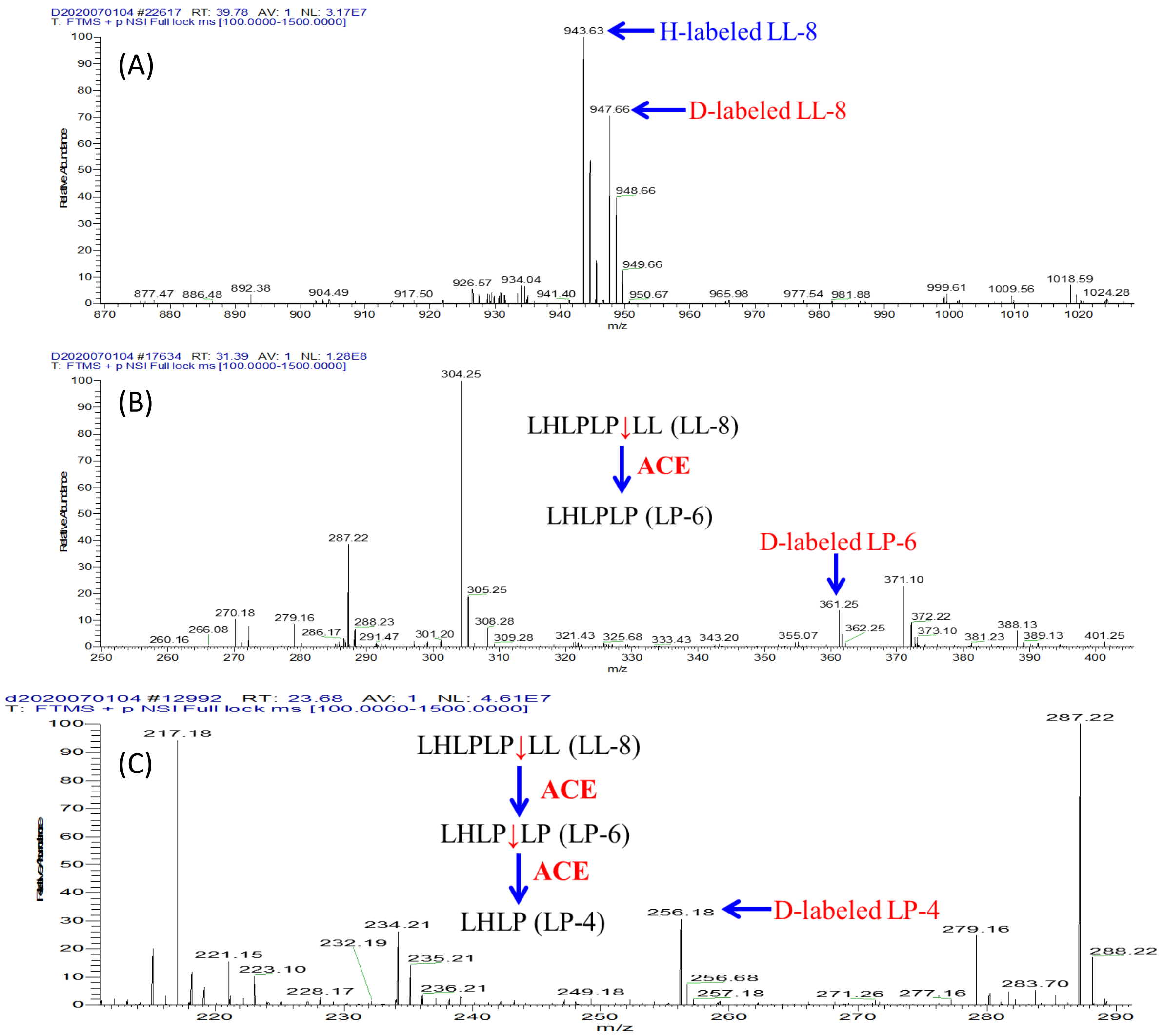

Figure S23-1. LC-MS/MS identification of ACE exogenous substrate LHLPLPLL (LL-8) and its products. (A) MS spectrum of D- and H-labeled LL-8; (B) MS spectrum of the product LHLPLP (LP-6); (C) MS spectrum of LHLP (LP-4).

Figure S23-2

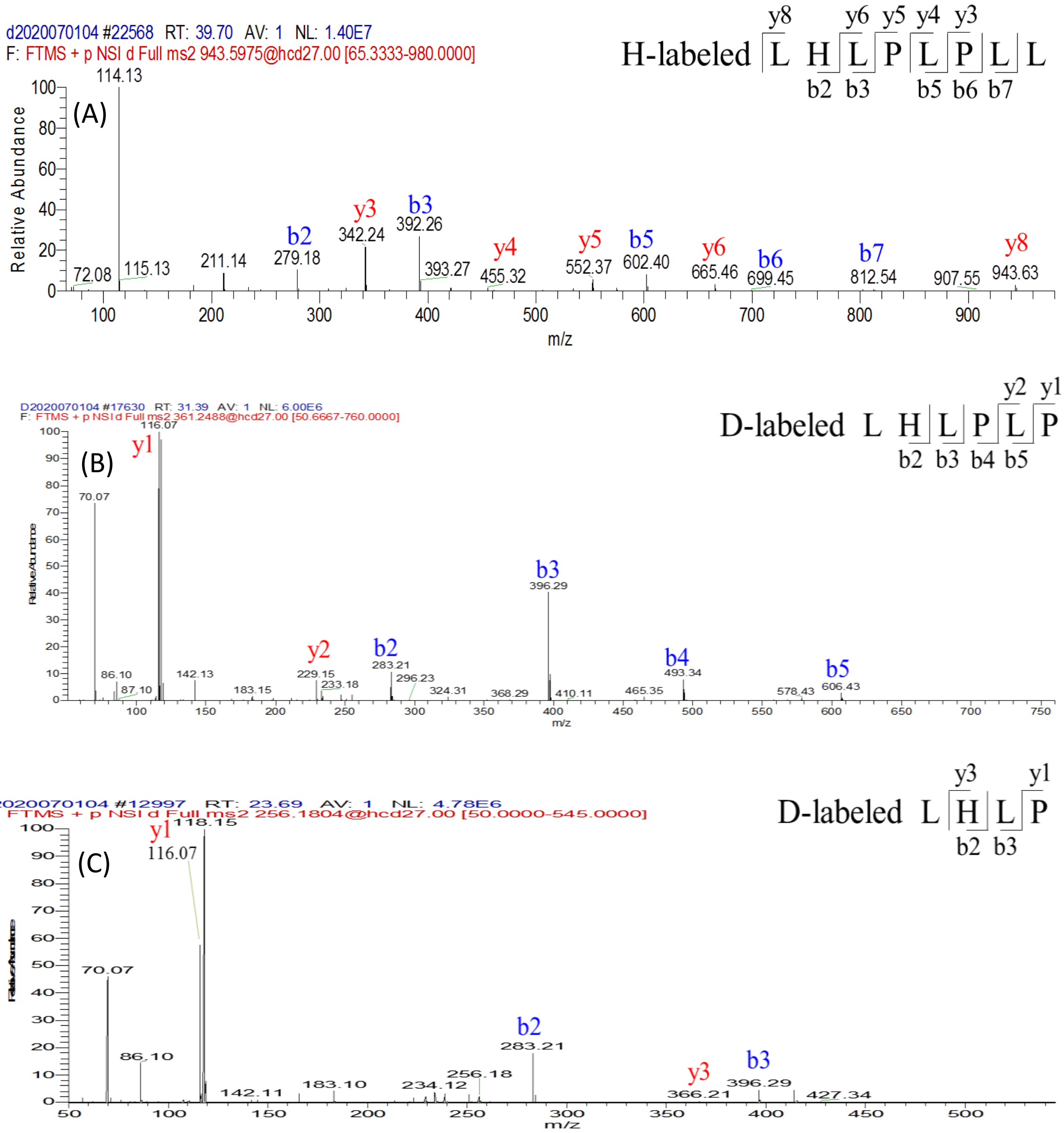

Figure S23-2. LC-MS/MS identification of ACE exogenous substrate LHLPLPLL (LL-8) and its products. (A) MS/MS spectrum of H-labeled LL-8 (m/z 943.5); (B) MS/MS spectrum of the product LP-6 (m/z 361.2); (C) MS spectrum of LP-4 (m/z 256.1).

Figure S24

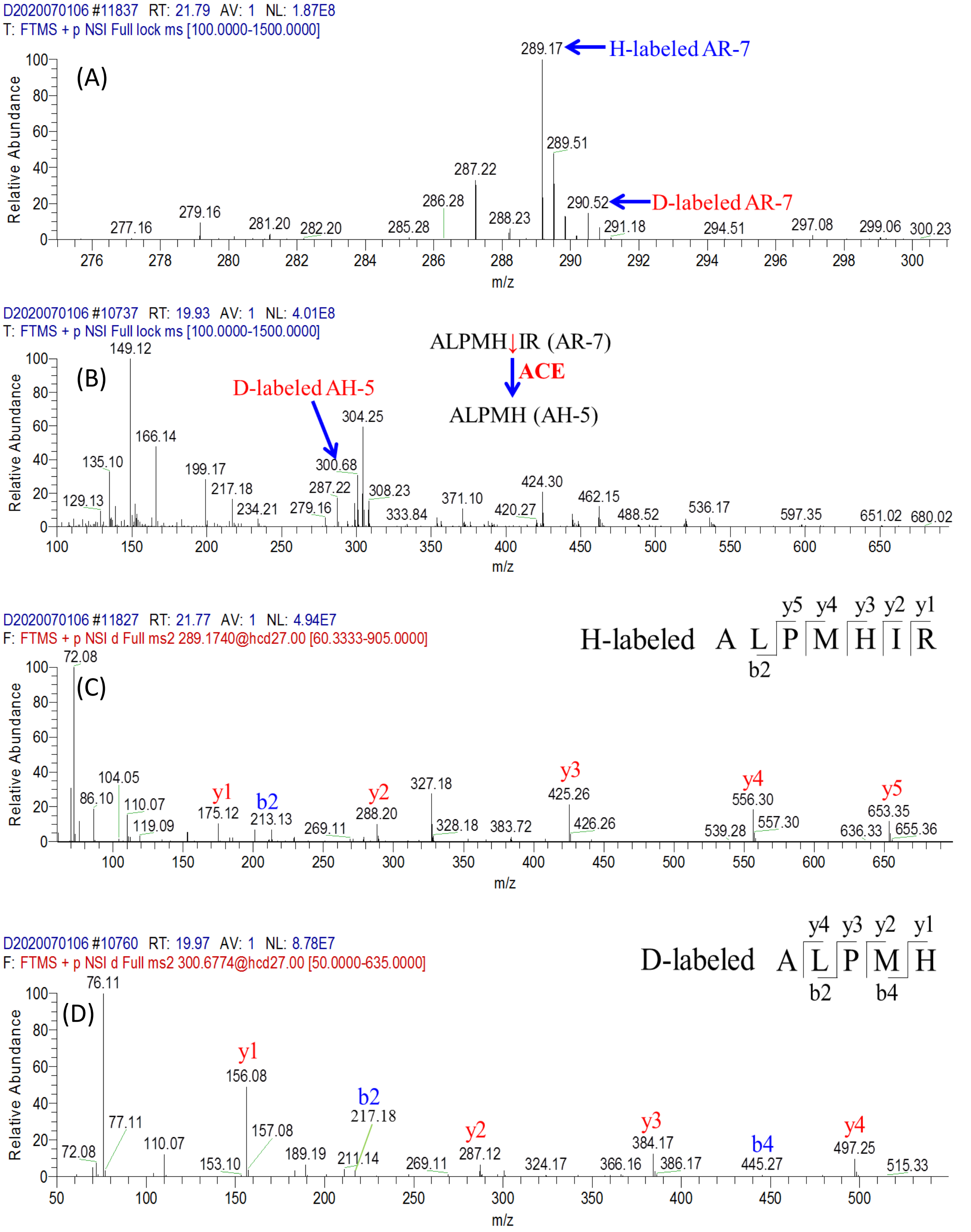

Figure S24. LC-MS/MS identification of ACE exogenous substrate ALPMHIR (AR-7) and its product. (A) MS spectrum of D- and H-labeled AR-7; (B) MS spectrum of the product ALPMH (AH-5); (C) MS/MS spectrum of AR-7 (m/z 289.1); (D) MS/MS of AH-5 (m/z 300.6).

Figure S25

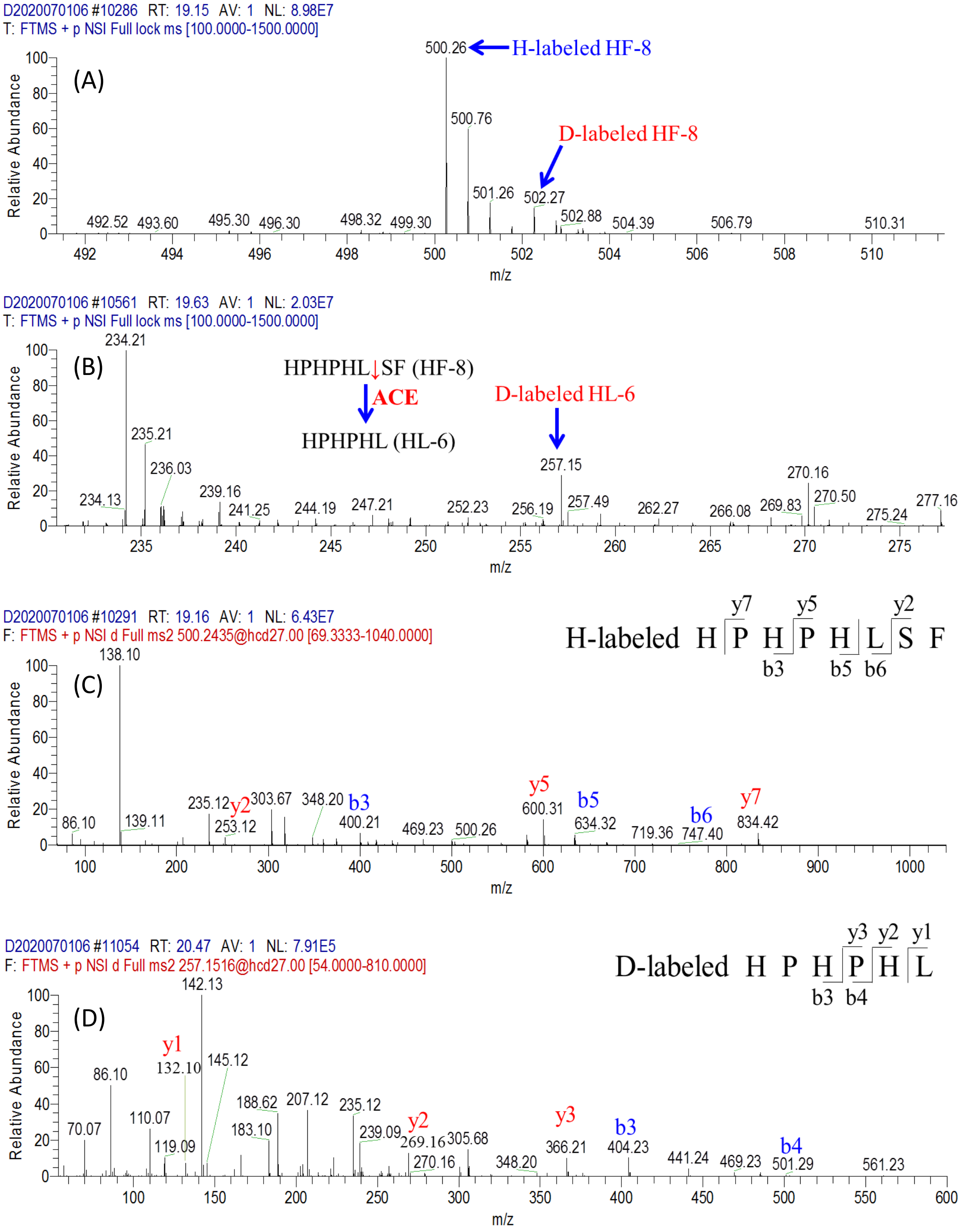

Figure S25. LC-MS/MS identification of ACE exogenous substrate HPHPHLSF (HF-8) and its product. (A) MS spectrum of D- and H-labeled HF-8; (B) MS spectrum of the product HPHPHL (HL-6); (C) MS/MS spectrum of HF-8 (m/z 500.2); (D) MS/MS of HL-6 (m/z 257.1).

Figure S26

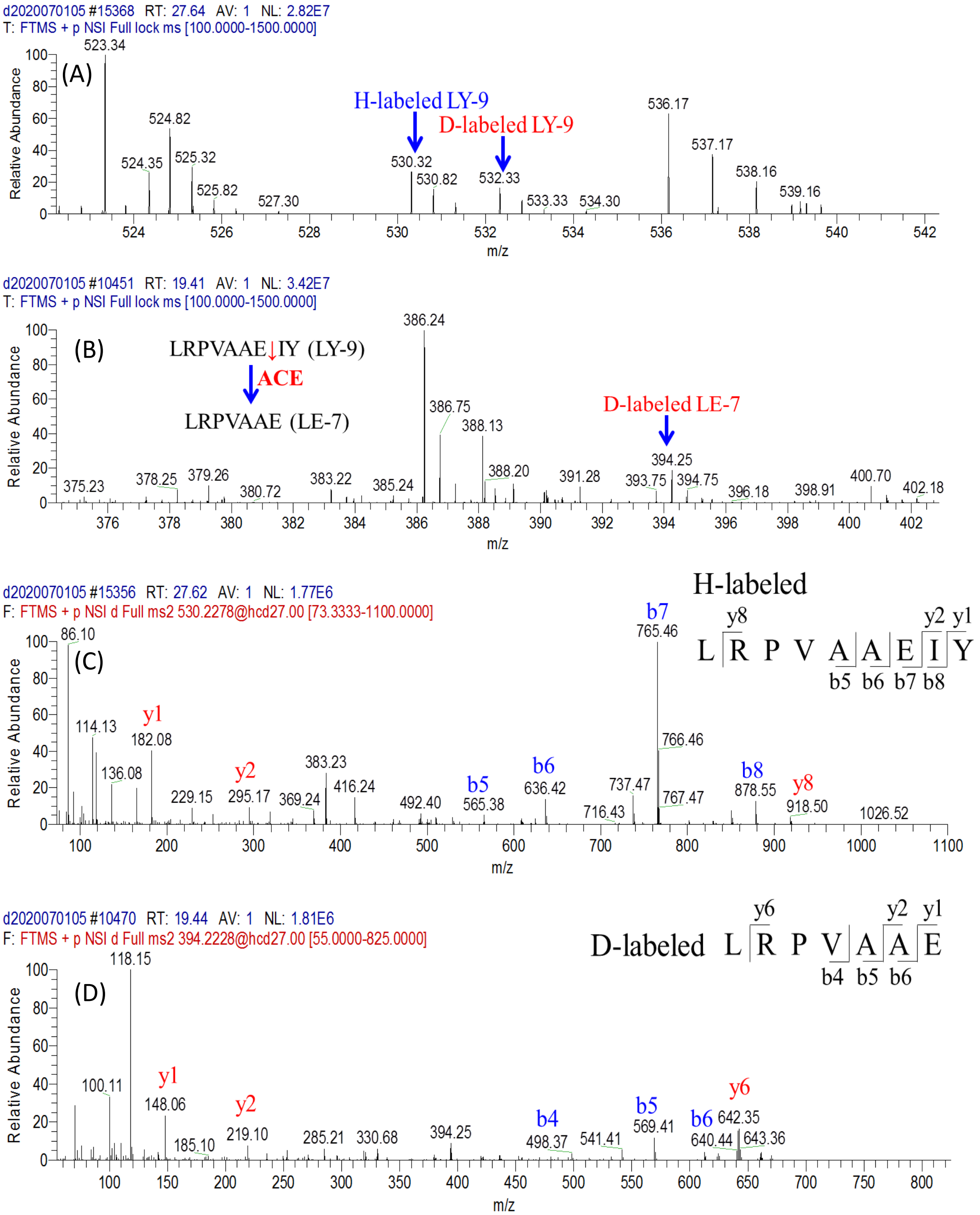

Figure S26. LC-MS/MS identification of ACE exogenous substrate LRPVAAEIY (LY-9) and its product. (A) MS spectrum of D- and H-labeled LY-9; (B) MS spectrum of the product LRPVAAE (LE-7); (C) MS/MS spectrum of LY-9 (m/z 530.2); (D) MS/MS of LE-7 (m/z 394.2).

Figure S27

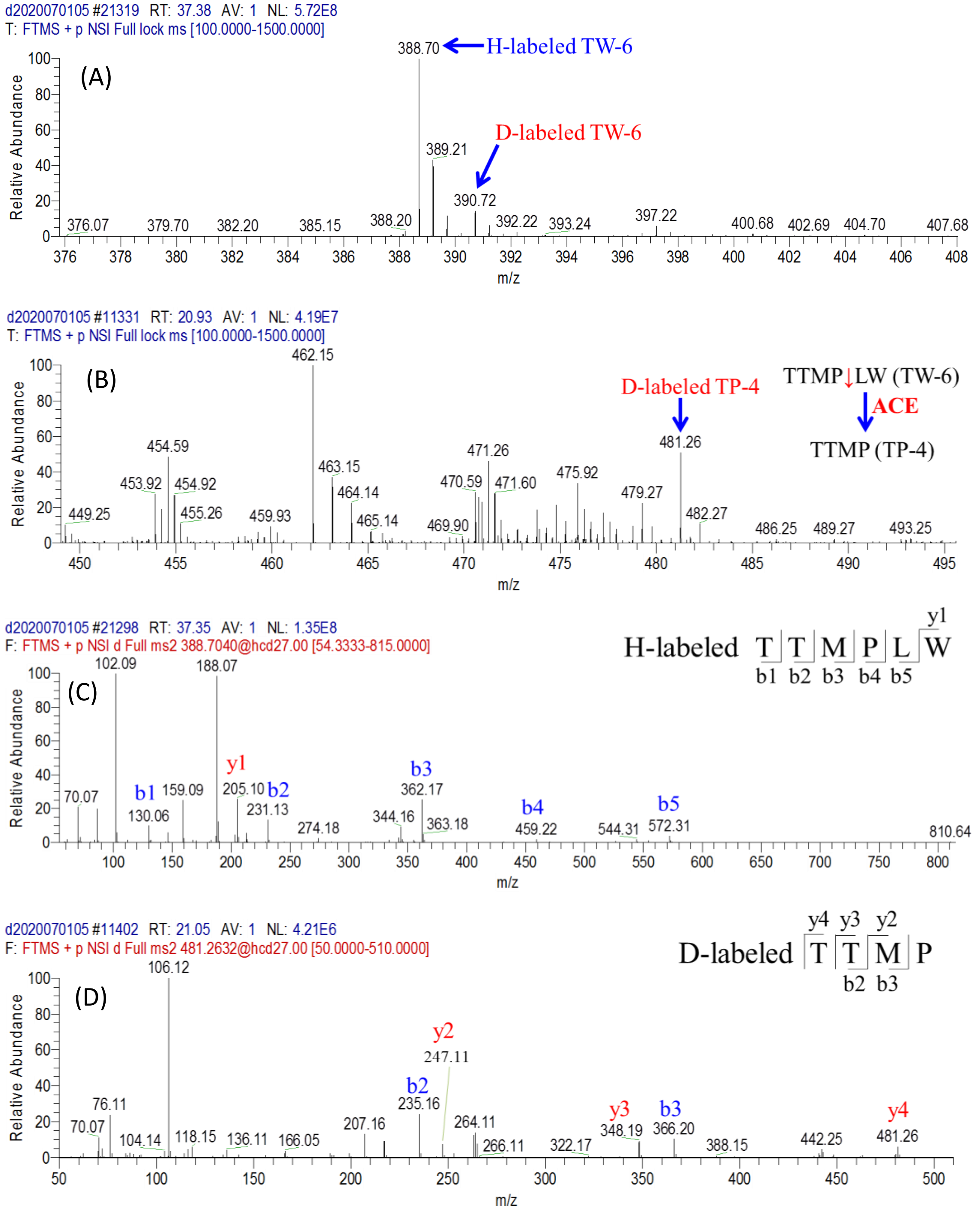

Figure S27. LC-MS/MS identification of ACE exogenous substrate TTMPLW (TW-6) and its product. (A) MS spectrum of D- and H-labeled TW-6; (B) MS spectrum of the product TTMP (TP-4); (C) MS/MS spectrum of TW-6 (m/z 388.7); (D) MS/MS of TP-4 (m/z 481.2).

Figure S28

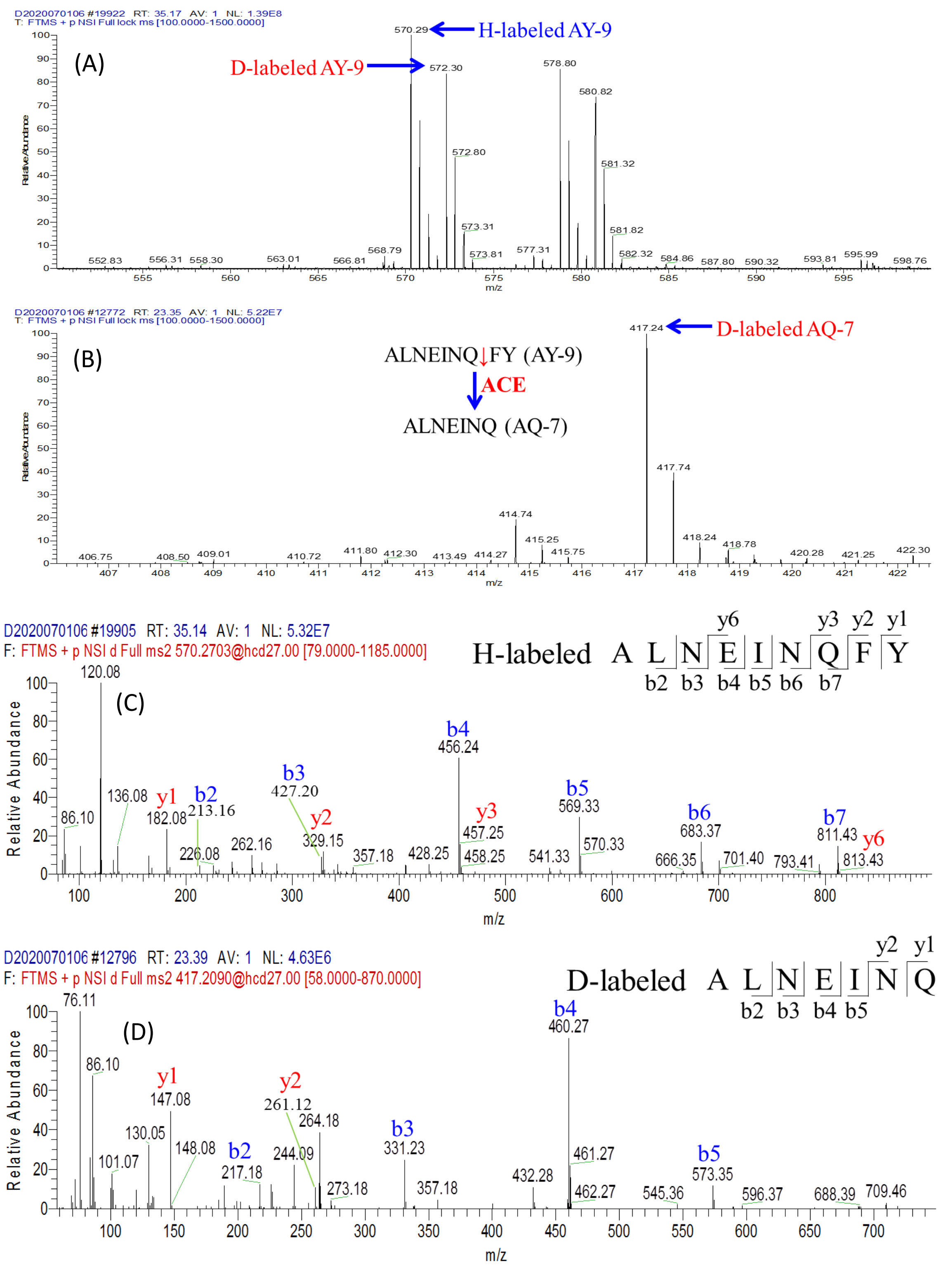

Figure S28. LC-MS/MS identification of ACE exogenous substrate ALNEINQFY (AY-9) and its product. (A) MS spectrum of D- and H-labeled AY-9 (B) MS spectrum of the product ALNEINQ (AQ-7); (C) MS/MS spectrum of AY-9 (m/z 570.2); (D) MS/MS of AQ-7 (m/z 417.2).

Figure S29

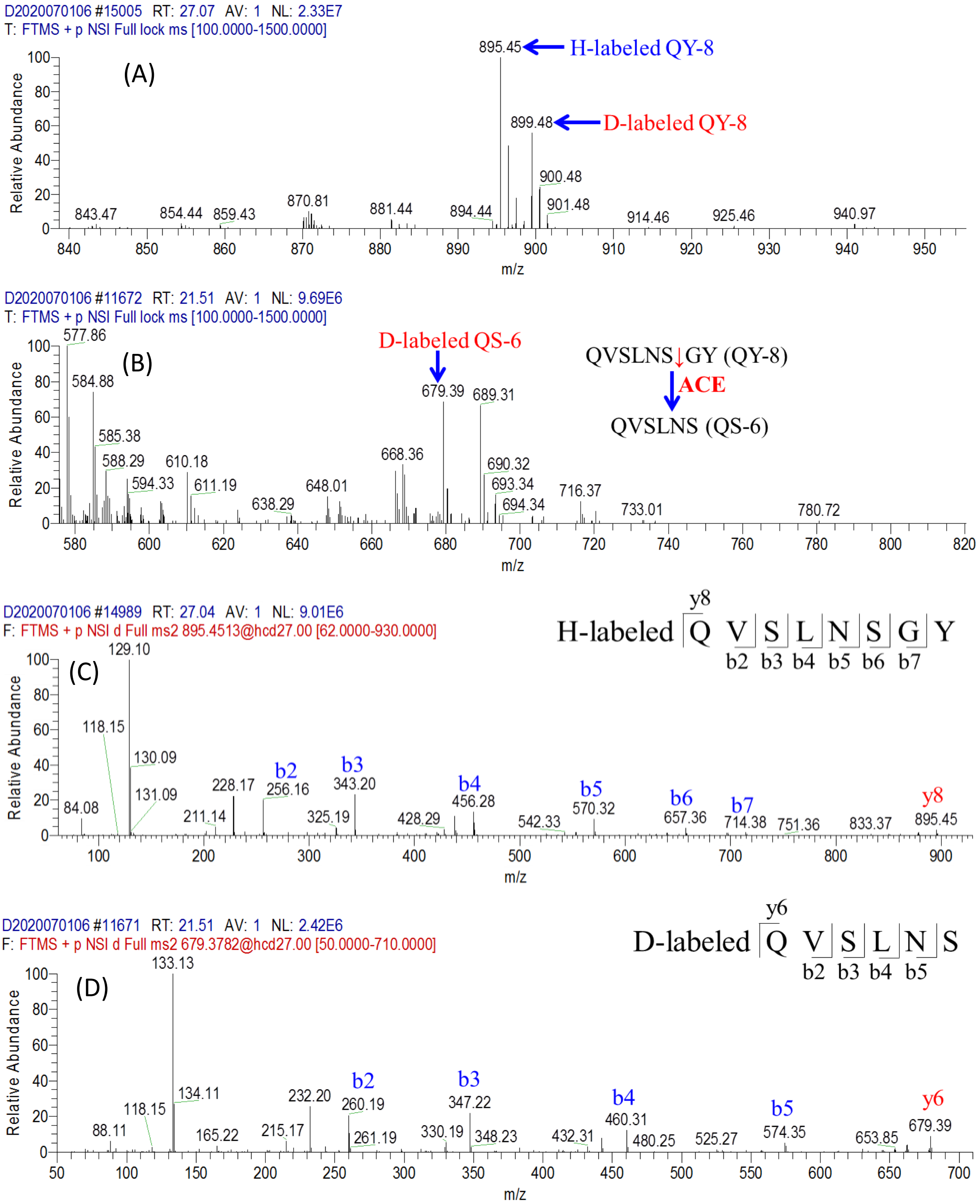

Figure S29. LC-MS/MS identification of ACE exogenous substrate QVSLNSGY (QY-8) and its product. (A) MS spectrum of D- and H-labeled QY-8 (B) MS spectrum of the product QVSLNS (QS-6); (C) MS/MS spectrum of QY-8 (m/z 895.4); (D) MS/MS of QS-6 (m/z 679.3).

Figure S30

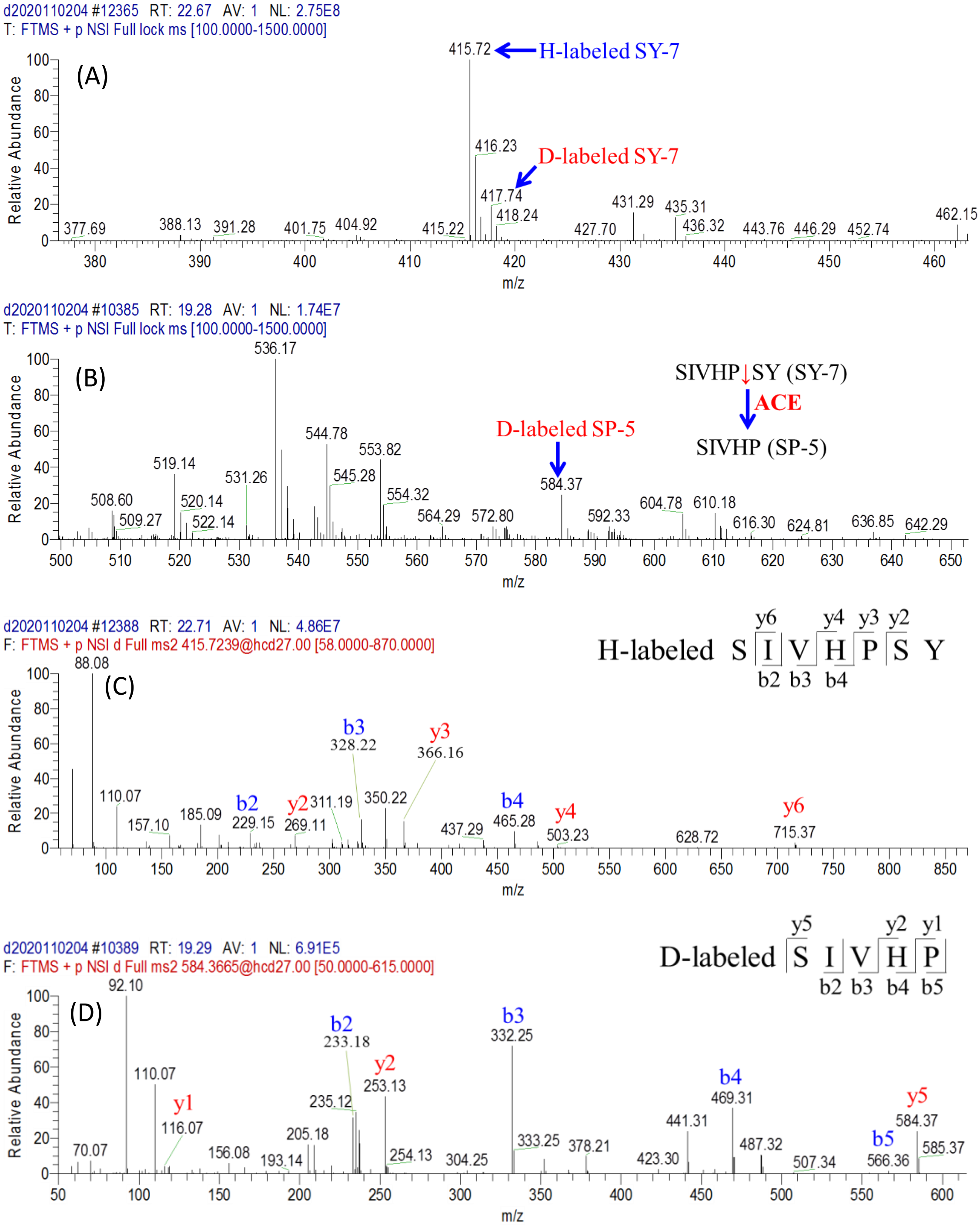

Figure S30. LC-MS/MS identification of ACE exogenous substrate SIVHPSY (SY-7) and its product. (A) MS spectrum of D- and H-labeled SY-7 (B) MS spectrum of the product SIVHP (SP-5); (C) MS/MS spectrum of SY-7 (m/z 415.7); (D) MS/MS of SP-5 (m/z 584.3).

Figure S31

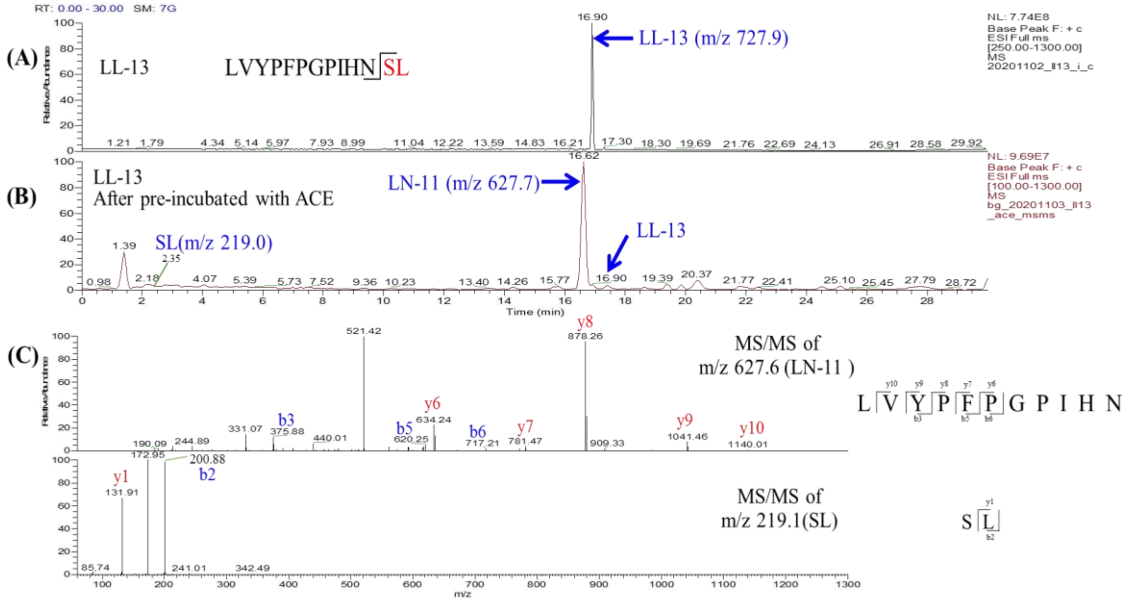

Figure S31. The reactivity confirmation of LVYPFPGPIHNSL (LL-13). (A) The full chromatogram of LL-13 (without ACE hydrolysis). (B) The full chromatogram of LL-13 after ACE incubation for 3h. (C) The MS/MS spectrum of LVYPFPGPIHN (LN-11) and SL.

Figure S32

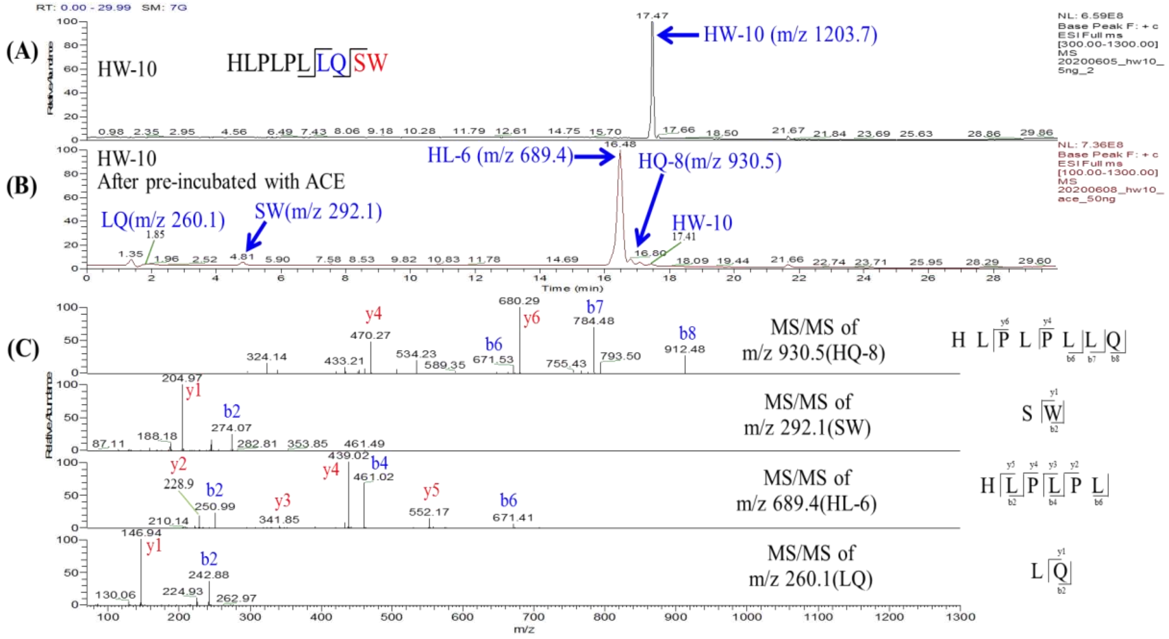

Figure S32. The reactivity confirmation of HLPLPLLQSW (HW-10). (A) The full chromatogram of HW-10 (without ACE hydrolysis). (B) The full chromatogram of HW-10 after ACE incubation for 3h. (C) The MS/MS spectra of HW-10’s products.

Figure S33

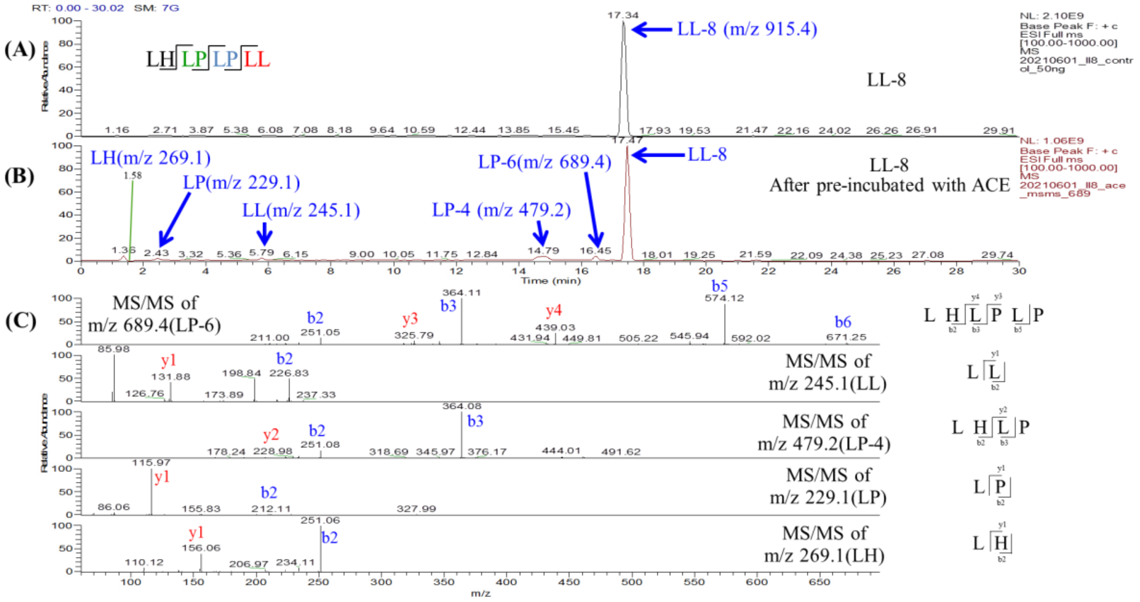

Figure S33. The reactivity confirmation of LHLPLPLL (LL-8). (A) The full chromatogram of LL-8 (without ACE hydrolysis). (B) The full chromatogram of LL-8 after ACE incubation for 3h. (C) The MS/MS spectra of LL-8's products.

Figure S34

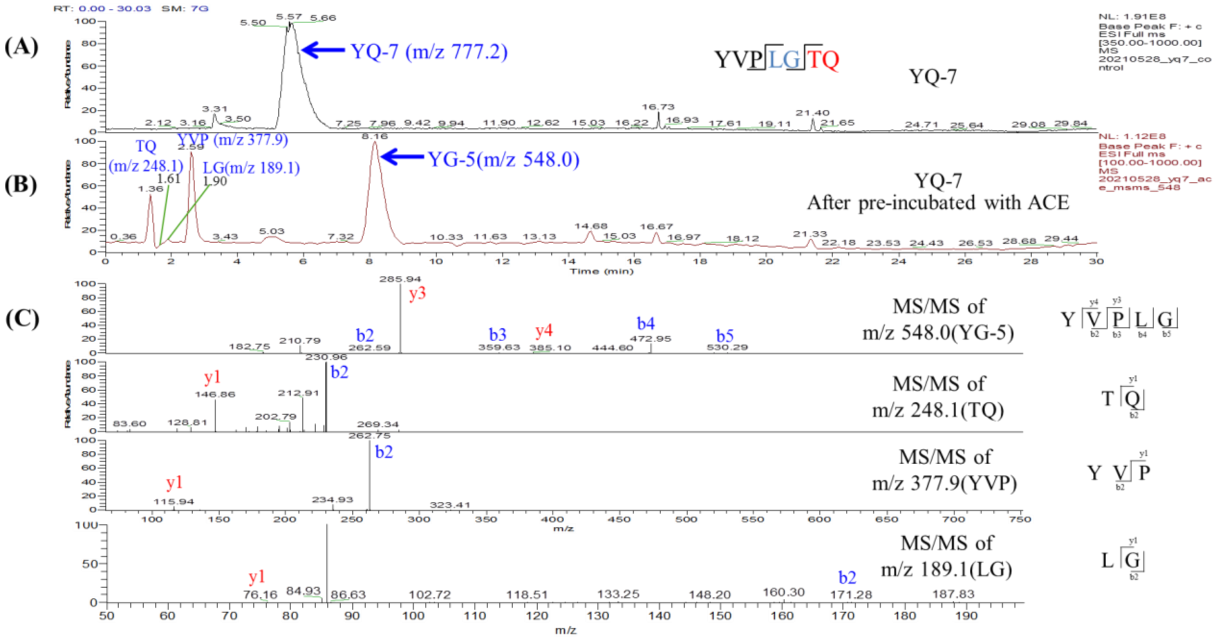

Figure S34. The reactivity confirmation of YVPLGTQ (YQ-7). (A) The full chromatogram of YQ-7 (without ACE hydrolysis). (B) The full chromatogram of YQ-7 after ACE incubation for 3h. (C) The MS/MS spectra of YQ-7's products.

Figure S35

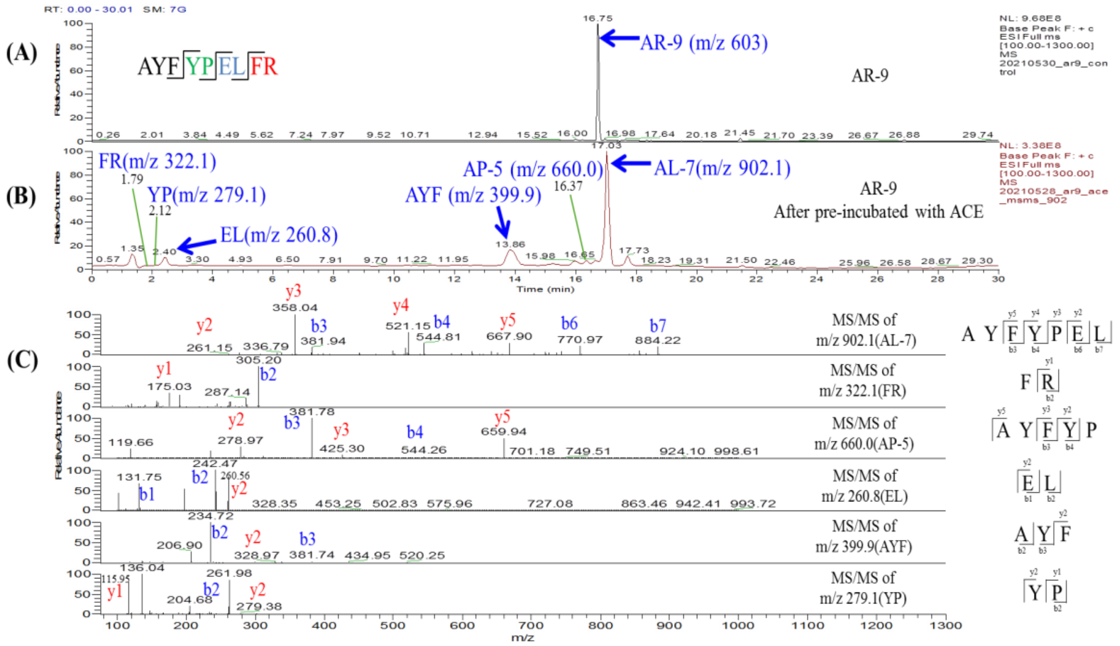

Figure S35. The reactivity confirmation of AYFYPELFR (AR-9). (A) The full chromatogram of AR-9 (without ACE hydrolysis). (B) The full chromatogram of AR-9 after ACE incubation for 3h. (C) The MS/MS spectra of AR-9's products.

Figure S36

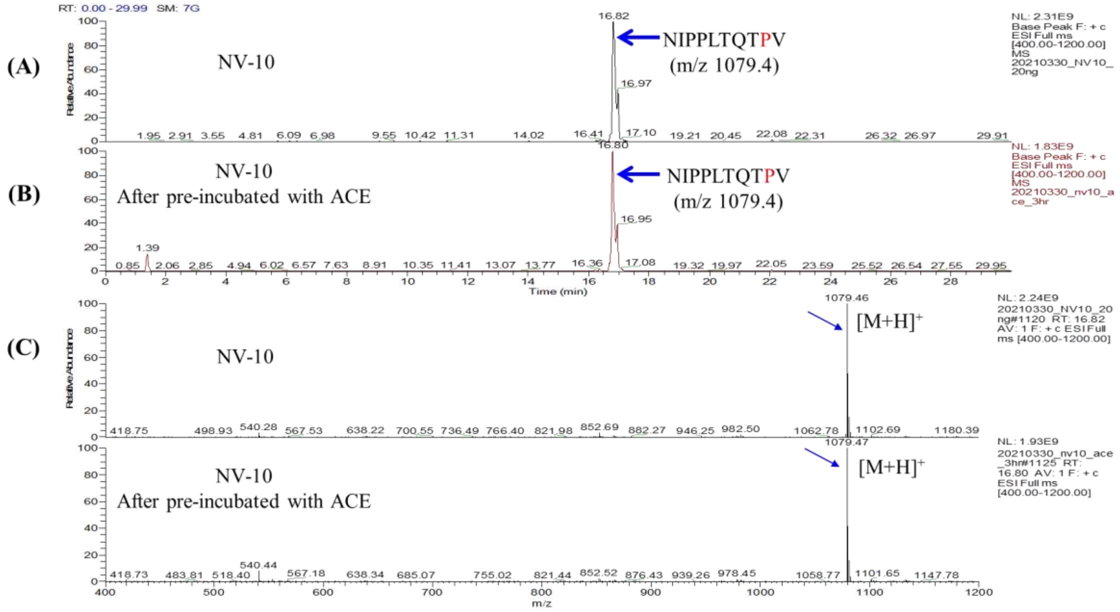

Figure S36. The reactivity confirmation of NIPPLTQTPV (NV-10) (Pro at P1' position). (A) The full chromatogram of NV-10 (without ACE hydrolysis). (B) The full chromatogram of NV-10 after ACE incubation for 3h (No any detectable product was observed). (C) The MS spectra of (A) and (B).

Figure S37

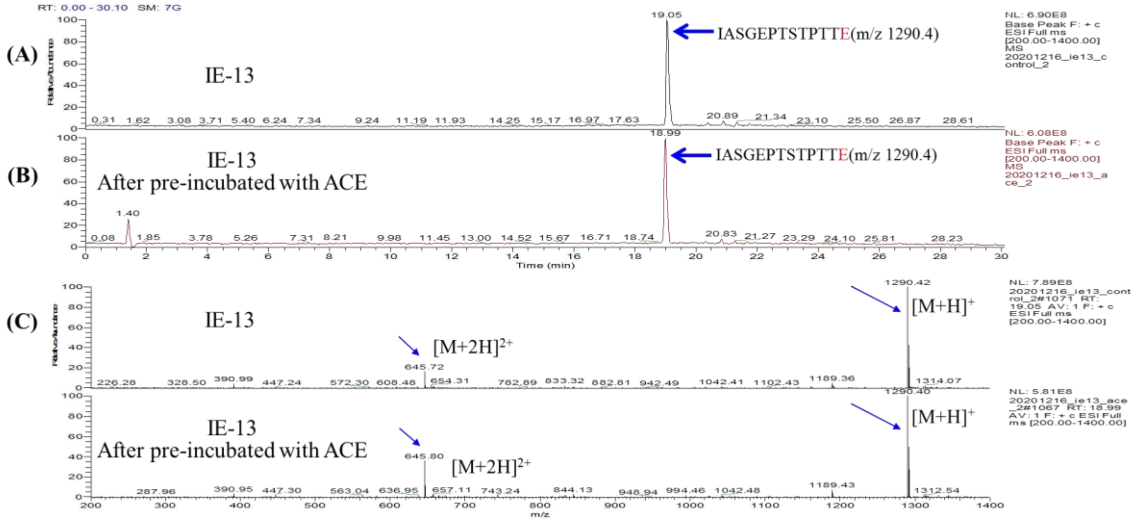

Figure S37. The reactivity confirmation of IASGEPTSTPTTE (IE-13) (Glu at P2’ position). (A) The full chromatogram of IE-13 (without ACE hydrolysis). (B) The full chromatogram of IE-13 after ACE incubation for 3h (No any detectable product was observed). (C) The MS spectra of (A) and (B).

F0gure S38

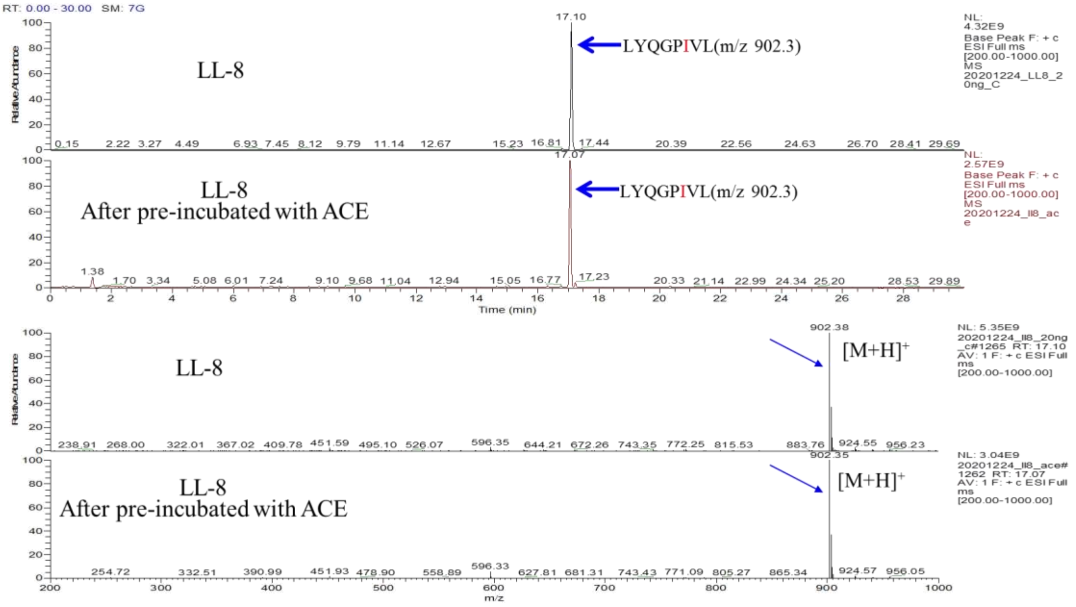

Figure S38. The reactivity confirmation of LYQGPIVL (LL-8) (Ile at P1 position). (A) The full chromatogram of LL-8 (without ACE hydrolysis). (B) The full chromatogram of LL-8 after ACE incubation for 3h (No any detectable product was observed). (C) The MS spectra of (A) and (B).

Figure S39

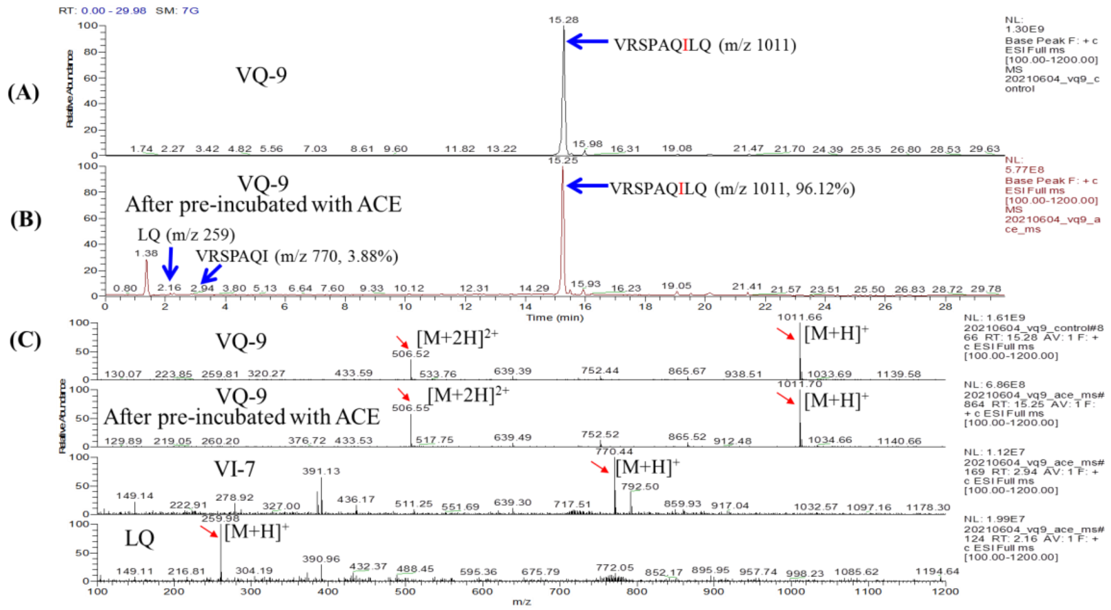

Figure S39. The reactivity confirmation of VRSPAQILQ (VQ-9) (Ile at P1 position). (A) The full chromatogram of VQ-9 (without ACE hydrolysis). (B) The full chromatogram of VQ-9 after ACE incubation for 3h (Trace amount of product was observed). (C) The MS spectra of (A), (B) and trace amount of products.

Figure S40

(A)

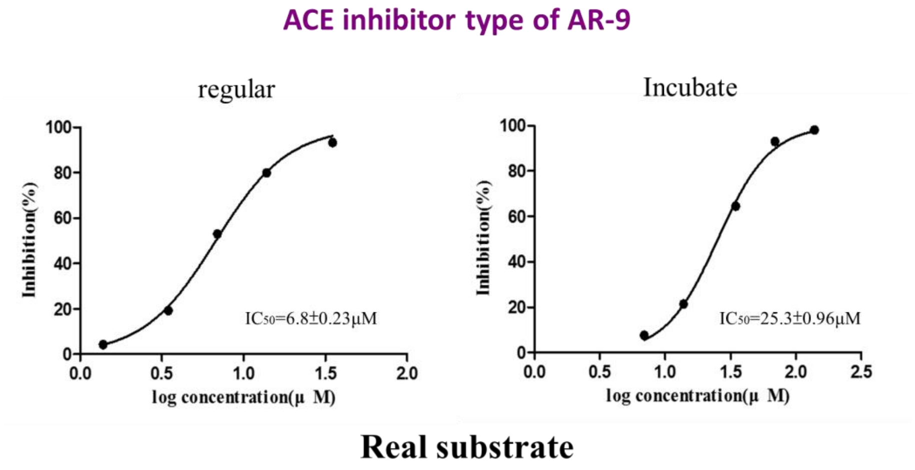

(B)

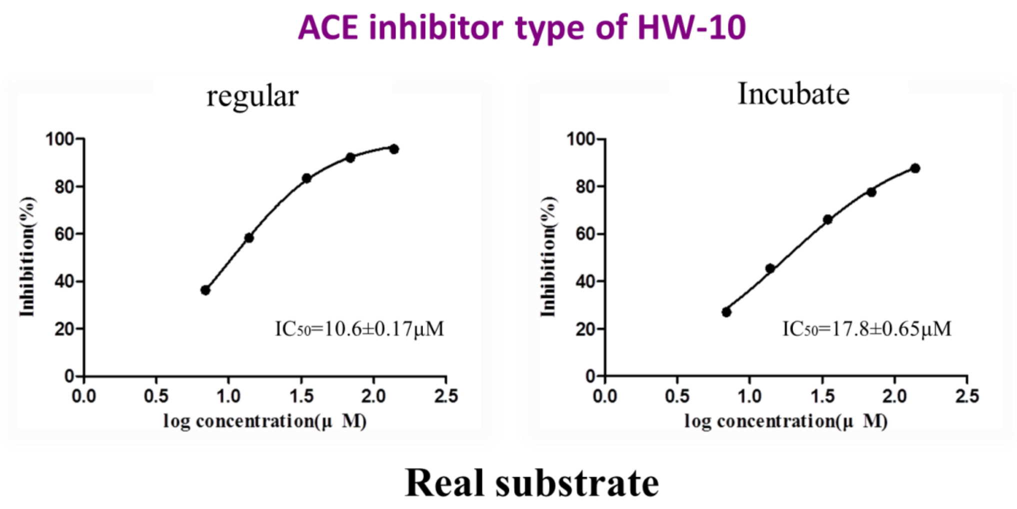

(C)

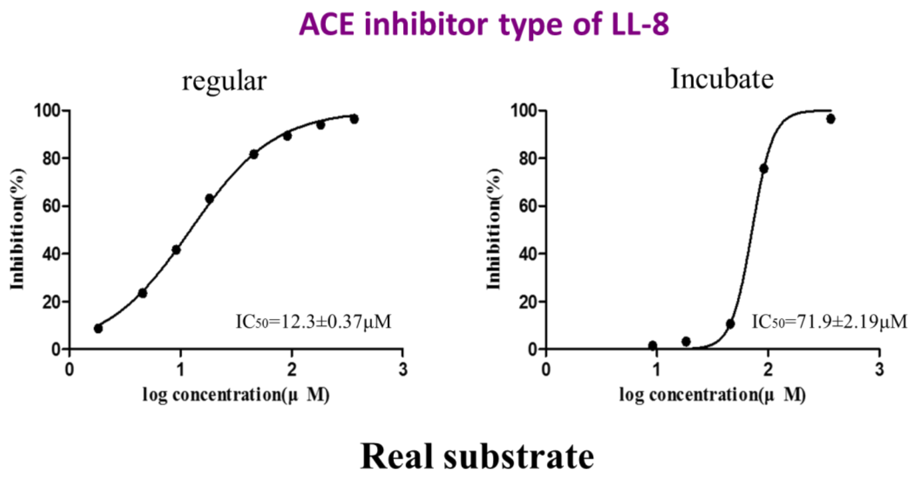

(D)

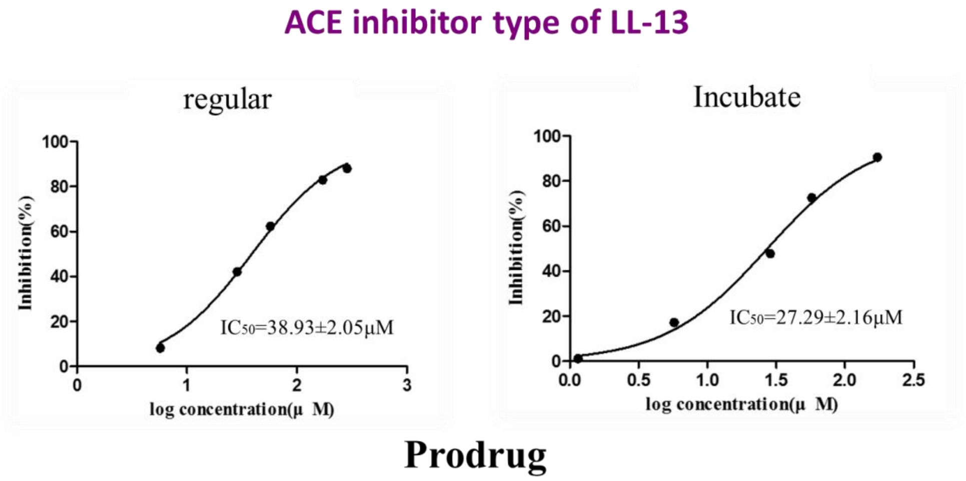

(E)

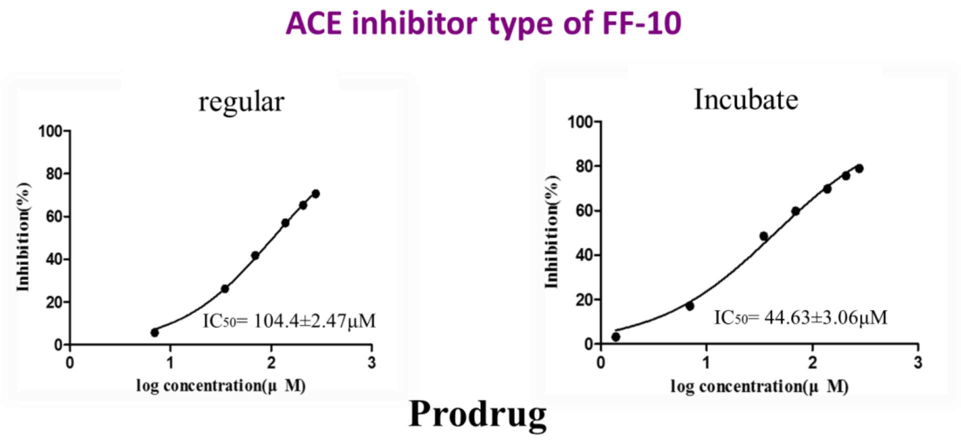

(F)

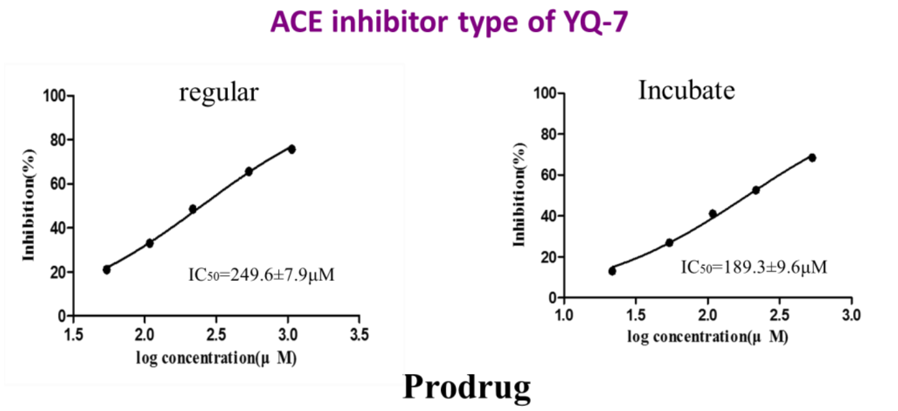

Figure S40. IC_{50} values of six peptides with and without ACE preincubation. (A) AR-9. (B) HW-10. (C) LL-8. (D) LL-13. (E) FF-10. (F) YQ-7.

Figure S41

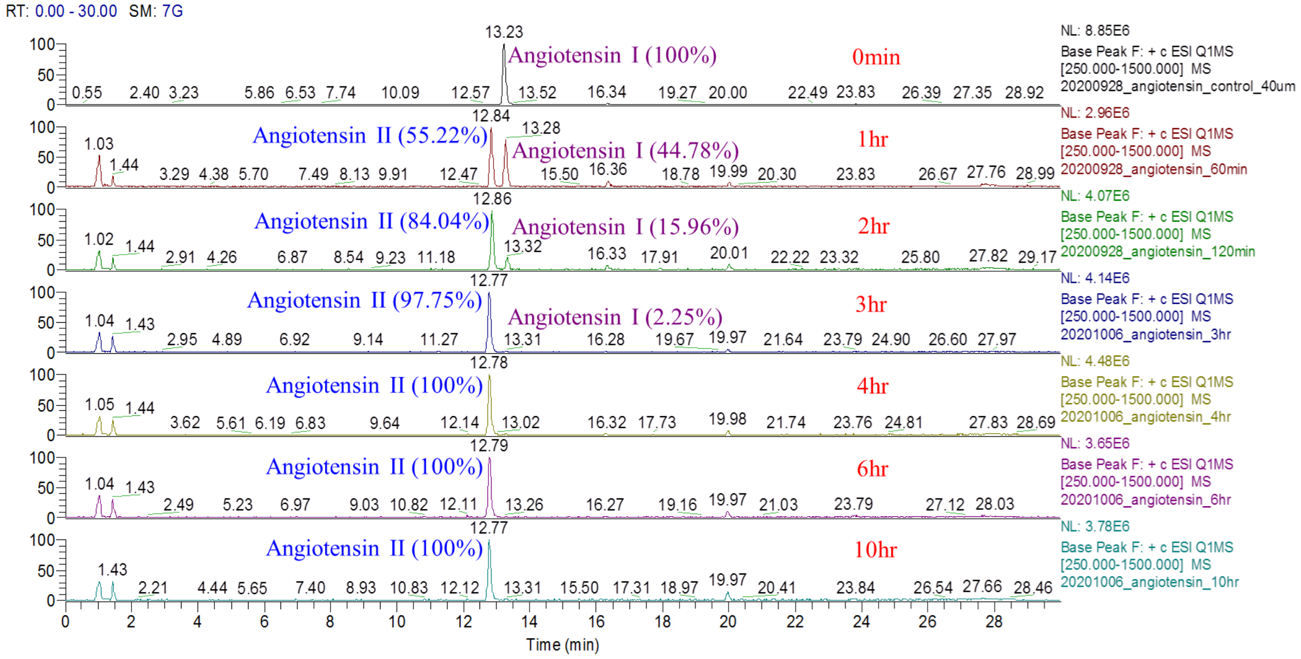

Figure S41. The remained abundance of synthetic Ang-I during the ACE hydrolysis monitored using LC-MS at various pre-incubation times.
